# Mapping of Dynamic Transcriptome Changes Associated With Silica-Triggered Autoimmune Pathogenesis in the Lupus-Prone NZBWF1 Mouse

**DOI:** 10.3389/fimmu.2019.00632

**Published:** 2019-03-29

**Authors:** Melissa A. Bates, Abby D. Benninghoff, Kristen N. Gilley, Andrij Holian, Jack R. Harkema, James J. Pestka

**Affiliations:** ^1^Department of Food Science and Human Nutrition, Michigan State University, East Lansing, MI, United States; ^2^Institute for Integrative Toxicology, Michigan State University, East Lansing, MI, United States; ^3^Department of Animal, Dairy and Veterinary Sciences and the School of Veterinary Medicine, Utah State University, Logan, UT, United States; ^4^Department of Biomedical and Pharmaceutical Sciences, Center for Environmental Health Sciences, University of Montana, Missoula, MT, United States; ^5^Department of Pathobiology and Diagnostic Investigation, Michigan State University, East Lansing, MI, United States; ^6^Department of Microbiology and Molecular Genetics, Michigan State University, East Lansing, MI, United States

**Keywords:** autoimmunity, NanoString, lung, systemic lupus erythematosus, silica, transcriptome

## Abstract

Crystalline silica (cSiO_2_) is a widely recognized environmental trigger of autoimmune disease. In the lupus-prone female NZBWF1 mouse, airway exposure to cSiO_2_ triggers pulmonary ectopic lymphoid neogenesis, systemic autoantibody elevation, and glomerulonephritis. Here we tested the hypothesis that upregulation of adaptive immune function genes in the lung precedes cSiO_2_-triggering of autoimmune disease in this model. The study include three groups of mice, as follows: (1) necropsied 1 d after a single intranasal instillation of 1 mg cSiO_2_ or vehicle, (2) necropsied 1 d after four weekly single instillations of 1 mg cSiO_2_ or vehicle, or (3) necropsied 1, 5, 9, or 13 weeks after four weekly single instillations of 1 mg cSiO_2_ or vehicle. NanoString nCounter analysis revealed modest transcriptional changes associated with innate and adaptive immune response as early as 1 d after a single cSiO_2_ instillation. These responses were greatly expanded after four weekly cSiO_2_ instillations. Concurrent with ectopic lymphoid neogenesis, dramatic increases in mRNAs associated with chemokine release, cytokine production, sustained interferon activity, complement activation, and adhesion molecules were observed. As disease progressed, expression of these genes persisted and was further amplified. Consistent with autoimmune pathogenesis, the time between 5 and 9 weeks post-instillation reflected an important transition period where considerable immune gene upregulation in the lung was observed. Upon termination of the chronic study (13 weeks), cSiO_2_-induced changes in transcriptome signatures were similarly robust in kidney as compared to the lung, but more modest in spleen. Transcriptomic signatures in lung and kidney were indicative of infiltration and/or expansion of neutrophils, macrophages, dendritic cells, B cells, and T cells that corresponded with accelerated autoimmune pathogenesis. Taken together, airway exposure to cSiO_2_ elicited aberrant mRNA signatures for both innate and adaptive immunity that were consistent with establishment of the lung as the central autoimmune nexus for launching systemic autoimmunity and ultimately, kidney injury.

## Introduction

Systemic lupus erythematosus (lupus) is a devastating autoimmune disease (AD) with a multifaceted etiology and widely variable disease manifestations. The initiating event in lupus is loss of tolerance to self-antigens which elicits production of autoreactive antibodies and formation of circulating immune complexes [reviewed in Pons-Estel et al. (1)]. These complexes deposit in tissues where they promote infiltration and activation of circulating mononuclear cells. Importantly, deposition of these immune complexes in the kidney results in glomerulonephritis that can advance to end-stage renal failure—a major cause of death in lupus patients.

Ectopic lymphoid structures (ELS) are hallmarks of AD that reflect the interface between unresolved inflammation and loss of self-tolerance. Unlike secondary lymphoid organs, ELS are induced at sites of unresolved inflammation, and thus, not found in pre-programmed places of the body. Their *de novo* formation facilitates accelerated initiation of an adaptive immune response by promoting antigen presentation and rapid activation of naïve B- and T-cells to remediate the offending agent at the site of inflammation (2–4). When organized within follicular dendritic cell (FDC) networks, ELS contain functional germinal centers that yield autoantibody-secreting plasma cells and promote AD.

Lupus and other ADs are strongly associated with an individual's genome. However, low concordance rates among monozygotic twins indicate that heredity is not the sole disease determinant (5). Recent studies suggest that the exposome (i.e., cumulative lifetime environmental exposures) is an understudied contributor to AD heterogeneity (6). Crystalline silica (cSiO_2_) is a widely recognized environmental trigger of autoimmunity. Over 2.3 million Americans are employed in occupations with high potential exposure to airborne cSiO_2_ particles, such as construction, stone cutting, foundries, and hydraulic fracturing (7). Epidemiological studies have established an etiological link between occupational exposure to cSiO_2_ and ADs, including lupus, rheumatoid arthritis, Sjögren's syndrome, scleroderma, and systemic vasculitis (8–12).

In lupus-prone female NZBWF1 mice, intranasal cSiO_2_ instillation triggered autoimmunity and glomerulonephritis 3 months earlier than vehicle-instilled controls (13, 14). Extensive inflammatory perivascular and peribronchial leukocyte infiltrates were evident in the lungs that contained numerous B cells and T cells corresponding to ectopic lymphoid neogenesis. Concomitant with pulmonary inflammation and ELS formation, bronchoalveolar lavage fluid (BALF) from cSiO_2_-exposed mice contained large numbers of neutrophils and macrophages as well as elevated concentrations of IgG, autoantibodies, IL-1β, IL-6, TNF-α, and B cell activating factor (BAFF). The latter responses were similarly reflected in the plasma and, therefore, indicate concomitant systemic autoimmunity. Collectively, these observations suggest that ELS in the lung might be central autoimmune triggering by cSiO_2_.

Recently, we characterized ELS development and autoimmunity over time in NZBWF1 mice that were intranasally instilled with cSiO_2_ weekly for 4 weeks and then sacrificed 1, 5, 9, or 13 weeks later (15). By week 1, inflammation comprising of B and T cells was observed in lungs of cSiO_2_-treated mice; these responses were continually amplified at 5, 9, and 13 weeks. Marked FDC networking appeared at 9 and 13 weeks PI. IgG^+^ plasma cells suggestive of mature germinal centers were evident at 13 weeks. Anti-dsDNA IgG in bronchial lavage fluid and plasma increased over the course of the experiment. cSiO_2_-induced glomerulonephritis with concomitant B-cell accumulation in the renal cortex was evident at 13 weeks PI. Accordingly, cSiO_2_ sequentially induced ectopic lymphoid neogenesis, germinal center development, systemic autoantibody elevation, and resultant glomerulonephritis in this unique preclinical model of environment-triggered lupus.

Little is known about how acute or chronic cSiO_2_ exposures impact global immune gene expression. To address this, we tested the hypothesis that upregulation of adaptive immune function genes in the lung precedes cSiO_2_-triggering of autoimmune disease in the NZBWF1 mouse. The NanoString nCounter platform is a targeted multiplex approach that enables measurement of up to 800 genes in a single reaction with high sensitivity and linearity across a broad dynamic range (16–18). Based on direct digital detection of mRNA molecules utilizing target-specific, color-coded probe pairs, this method bridges the gap between RNAseq and targeted RT-PCR expression profiling. Herein, we employed the nCounter platform to: (1) relate temporal changes in immune-related transcriptome signatures in the lung following acute and short-term repeated intranasal cSiO_2_ exposure to ELS development and autoimmune pathogenesis in female NZBWF1 mice and (2) compare the terminal transcriptome signatures in the lung to those in the spleen and kidney.

## Materials and Methods

### Animals and Diets

Experimental protocols were approved by the Institutional Animal Care and Use Committee at Michigan State University in accordance with the National Institutes of Health guidelines (AUF #01/15-021-00). Female 6-week-old lupus-prone NZBWF1 mice were provided by Jackson Laboratories (Bar Harbor, ME) and randomized into experimental groups. Mice were housed four per cage and fed AIN-93G diet (Dyets Inc., Bethlehem, PA) as described previously (13, 15). Mice had free access to food and water and were kept at constant temperature (21–24°C) and humidity (40–55%) under a 12/h light/dark cycle.

### cSiO_2_

cSiO_2_ (Min-U-Sil-5, 1.5–2.0 μm average particle size, Pennsylvania Sand Glass Corporation, Pittsburgh, PA) was acid washed and dried before addition of sterile phosphate buffered saline (PBS). Stock suspensions were prepared fresh in PBS prior to exposure and suspensions sonicated and vortexed for 1 min before intranasal instillation.

### Experimental Design

**Figure 1:**
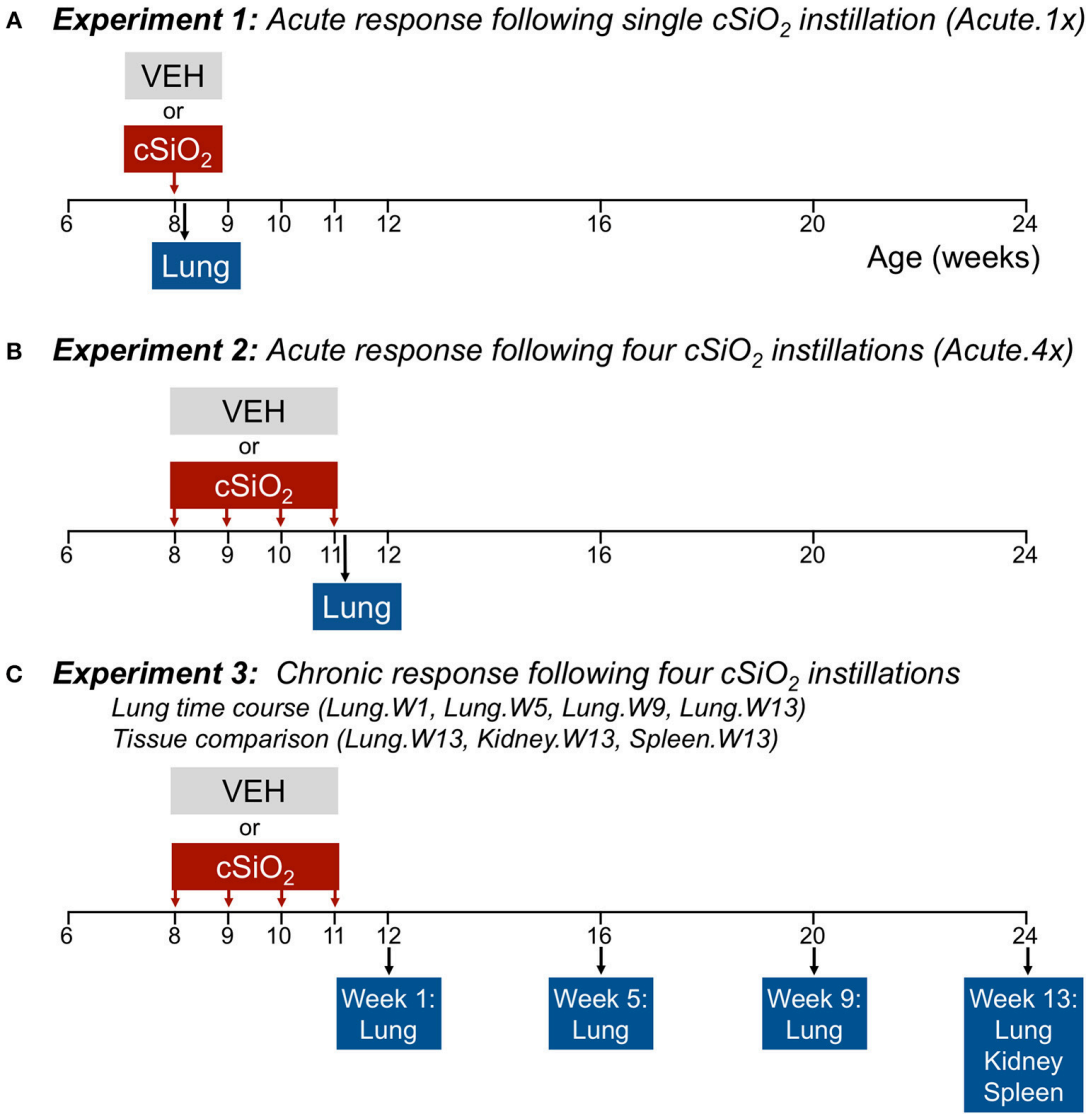
Design of experiments. At 8 weeks of age, female NZBWF1 mice were dosed intranasally with 25 μl PBS (VEH) or 25 μl PBS containing 1.0 mg cSiO_2_ once (**A**; experiment 1) or weekly for 4 weeks (**B,C**; experiments 2 and 3). Cohorts (*n* = 7–8) of mice were euthanized and necropsied 1 day (experiment 1 and 2) following the only/final instillation or 12, 16, 20, or 24 weeks of age corresponding to 1, 5, 9, or 13 weeks post the final instillation (experiment 3). Tissues obtained for nCounter digital transcript counting (NanoString PanCancer Immune Profiling gene set) are indicated above.

Figure 1 depicts the experimental design for this study. To model acute response to a single dose of cSiO_2_, (Acute.1x) groups of 8-week-old mice (*n* = 8/gp) were anesthetized with 4% isoflurane and intranasally instilled with 1.0 mg cSiO_2_ in 25 μl PBS or 25 μl PBS vehicle (VEH) as described previously (13). To capture the acute response to short-term repeated exposure to cSiO_2_, a second cohort of mice received 1.0 mg cSiO_2_ or VEH once weekly for 4 weeks (Acute.4x). Each cohort was sacrificed 24 h after final instillation. The caudal lung lobe was removed, held in RNAlater (Thermo Fisher Scientific, Wilmington, DE) overnight at 4°C, and then stored at −80°C for RNA multiplexing.

To visualize how cSiO_2_ impacts autoimmune gene expression over the long term, we used tissues from our recently published investigation that focused on the histopathological changes over time in cSiO_2_-treated NZBWF1 mice (15). In that study, beginning at 8 weeks of age, groups of mice on were exposed to VEH or cSiO_2_ weekly for 4 weeks. Thereafter, cohorts of mice were sacrificed over time at 1, 5, 9, and 13 weeks post final cSiO_2_ exposure. One mouse in the 5 week VEH group died of unknown causes during the course of the experiment; it did not show the typical signs of sickness including loss of body weight before death. Tissues were collected at stored in RNAlater (ThermoFisher Scientific). Lungs were analyzed at 1 (Lung.W1), 5 (Lung.W5), 9 (Lung.W9), and 13 (Lung.W13) weeks post cSiO_2_ exposure, and spleen (Spleen.W13) and kidney (Kidney.W13) at 13 weeks. These time points were selected to coincide with pathological changes previously described in this model following cSiO_2_ exposure prior to and during onset of glomerulonephritis (13–15).

### Gene Expression Analysis With NanoString nCounter

Total RNA was extracted from lung, spleen, and kidney with TriReagent (Sigma Aldrich, St. Louis, MO) per manufacturer's instructions. Resultant RNA was further purified and genomic DNA removed by RNeasy Mini Kit with DNase treatment (Qiagen, Valencia, CA). RNA was dissolved in nuclease-free water and quantified using a NanoDrop-1000 (Thermo Fisher). Samples were then analyzed for RNA integrity using a LabChip Gx Analyzer (Caliper Life Sciences, Waltham, MA). Samples with RIN values >7.0 were included for gene expression analysis.

The NanoString nCounter (NanoString Technologies, Inc., Seattle WA) was used to assess the effects of cSiO_2_ exposure on acute and chronic changes in immune gene expression. This method was selected over other targeted multiplex approaches because it (i) requires minimal sample preparation, (ii) does not require cDNA conversion or target amplification—both major sources of variation in conventional qRT-PCR approaches, (iii) has robust user software for gene analysis, and (iv) has been repeatedly demonstrated to correlate well other microarray platforms (19–21). Briefly, RNA (*n* = 7–8/gp) was analyzed using the nCounter Mouse PanCancer Immune Profiling Panel (catalog # 115000142), which includes 770 immune-related genes, 40 housekeeping genes, and 6 positive controls. Assays were performed and quantified on the nCounter MAX system, sample preparation station, and digital analyzer (NanoString Technologies) according to the manufacturer's instructions. Briefly, reporter and capture probes were hybridized to target analytes for 16 h at 65°C. After hybridization, samples were washed to remove excess probes. Then, the purified target-probe complexes were aligned and immobilized onto the nCounter cartridge, and the transcripts were counted via detection of the fluorescent barcodes within the reporter probe. Probe annotations are provided in Supplementary File 1.

Raw gene expression data were analyzed using NanoString's software nSolver v3.0.22 with the Advanced Analysis Module v2.0. Background subtraction was performed using the eight included negative controls included with the module. Genes with counts below a threshold of 2σ of the mean background signal were excluded from subsequent analysis (Supplementary Figures 1–3). Data normalization was performed on background-subtracted samples using internal positive controls and selected housekeeping genes that were identified with the geNorm algorithm (https://genorm.cmgg.be/).

Ratios of transcript count data were generated for cSiO_2_-treated mice vs. VEH control as follows: acute response in lung tissue of cSiO_2_-treated (single dose) mice vs. dosing-matched vehicle controls 1 day post instillation (Acute.1x); acute response in lung tissue of cSiO_2_-treated (four weekly doses) mice vs. dosing-matched control 1 day post instillation (Acute.4x); chronic response in lung tissue of cSiO_2_-treated (four weekly doses) mice vs. dosing- and time-matched vehicle controls 1, 5, 9, or 13 weeks post instillation (Lung.W1, Lung.W5, Lung.W9, Lung.W13, respectively); chronic response in kidney tissue of cSiO_2_-treated (four weekly doses) mice vs. dosing- and time-matched vehicle controls 13 weeks post instillation (Kidney.W13); chronic response in spleen tissue of cSiO_2_-treated (four weekly doses) mice vs. dosing- and time-matched vehicle controls 13 weeks post instillation (Spleen.W13). Ratios were then log_2_ transformed for downstream analysis.

### Data Analysis

Differential gene expression analyses were performed using nSolver, which employs several multivariate linear regression models to identify significant genes (mixture negative binomial, simplified negative binomial, or log-linear model) as outlined in Supplementary Figure 4. Resulting *p* values were adjusted using the Benjamini-Hochberg (BH) method to control the false discovery rate. Statistically significant, differentially expressed genes were defined as those with expression levels corresponding to a log_2_ ratio >1 or < −1 and BH *q* value < 0.05 for cSiO_2_ treatments compared to the appropriate vehicle control group (Supplementary Figure 5). Outputs from nSolver differential expression analysis are provided in Supplementary File 2. Venn diagrams of significant differentially expressed genes in cSiO_2_ groups were generated using BioVenn (22) or Venny v2.1 (23).

To assess impact of cSiO_2_ treatment on annotated gene sets, global and directed significance scores were calculated for each pathway. The global significance score for each gene set was calculated as the square root of the mean squared *t*-statistic of genes, as determined by the differential gene expression analyses. The global score estimates the cumulative evidence for the differential expression of genes in a pathway. A directed significance score was also calculated by taking into account the sign of the *t*-statistics. Directed significance scores near zero may have many highly regulated genes, but no apparent tendency for those genes to be over- or under-expressed collectively. Pathway scores were used to summarize data from a pathway's genes into a single score. Pathway scores were calculated as the first principal component of the pathway genes' normalized expression and standardized by Z scaling. ClustVis (24) was used to perform unsupervised hierarchical cluster analyses (HCC) and principal components analyses (PCA) using log_2_ transcript count data. Supplementary Files 3–4 provide summary tables for all significance and pathway *Z* scores.

### Immune Cell Profiling

A significant discovery feature of the NanoString platform is cell profiling, which uses marker genes expressed stably and specifically in immune cell types to estimate relative abundance in sample groups measured as the average log-scale expression of their characteristic genes (25). Note that this analysis does not provide information on the absolute number of immune cells in a sample. Cell types and genes compatible with the PanCancer Immune Profiling Panel included leukocytes (*Ptprc*), B cells (*Ms4a1, Tnfrsf17*), T cells (*Cd3d, Cd3e, Cd3g, Cd6, Sh2d1a*), Th1 cells (*Tbx21*), T reg (*Foxp3*), CD8 T cells (*Cd8a, Cd8b1*), exhausted CD8 cells (*Cd244, Eomes, Lag3*), cytotoxic cells (*Ctsw, Gzma, Gzmb, Klrb1, Klrd1, Klrk1, Prf1*), dendritic cells (*Ccl2, Cd209e, Hsd11b1*), macrophages (*Cd163, Cd68, Cd84*), mast cells (*Ms4a2*), neutrophils (*Csf3r, Fcgr4*), and NK cells (*Ncr1, Xcl1*). The nCounter Advanced Analysis module reports *p* value confidence thresholds for reporting for cell types with multiple markers, though gene sets with high *p*-values may be useful if use of the biomarker genes is supported by prior studies. For this study, we report results for the immune cell types listed above, including those with threshold *p* values >0.05 as those data may be useful in future exploratory work. Supplementary File 5 provides the log_2_ scores for cell type profiling.

### Network Analysis

Network analyses for interactions among significant genes were performed using STRING database version 10.5 (http://string-db.org/), which curates both experimental and predicted gene interactions. Only interactions among significant genes identified by the nSolver data analysis were considered with the confidence level for associations set at ≥0.7. Clusters were identified using the Markov Cluster (MCL) algorithm with inflation parameter of 1.5. Networks generated by STRING were visualized with Cytoscape v3.0, with nodes representing significant genes and edge width indicating the combined interaction score. Supplementary File 6 provides data for STRING-db networks and the predicted clusters.

### Correlation Analysis

Spearman rank correlations were performed to examine overall patterns in the gene expression profiles compared to histopathological lesions in lung tissues and markers of immune cells. Correlation analysis was performed using *cor* and *corrplot* functions in R (www.R-project.org). Spearman ρ values were calculated using individual sample pathway *Z* scores and phenotype data: histopathology scores (lymphoid aggregates, ectopic lymphoid structures, alveolar proteinosis, alveolitis, type 2 cell hyperplasia, and mucus cell metaplasia) or percent positive staining (CD3, CD45R, and CD21/35) for lung tissues obtained from mice at 1, 5, 9, and 13 weeks post instillation (15). A significant correlation was inferred when ρ > 0.5 or < −0.5 and *p* < 0.05.

## Results

### Acute mRNA Responses Following Single and Short Term cSiO_2_ Exposure Indicate Innate and Adaptive Immune Activation

**Figure 2:**
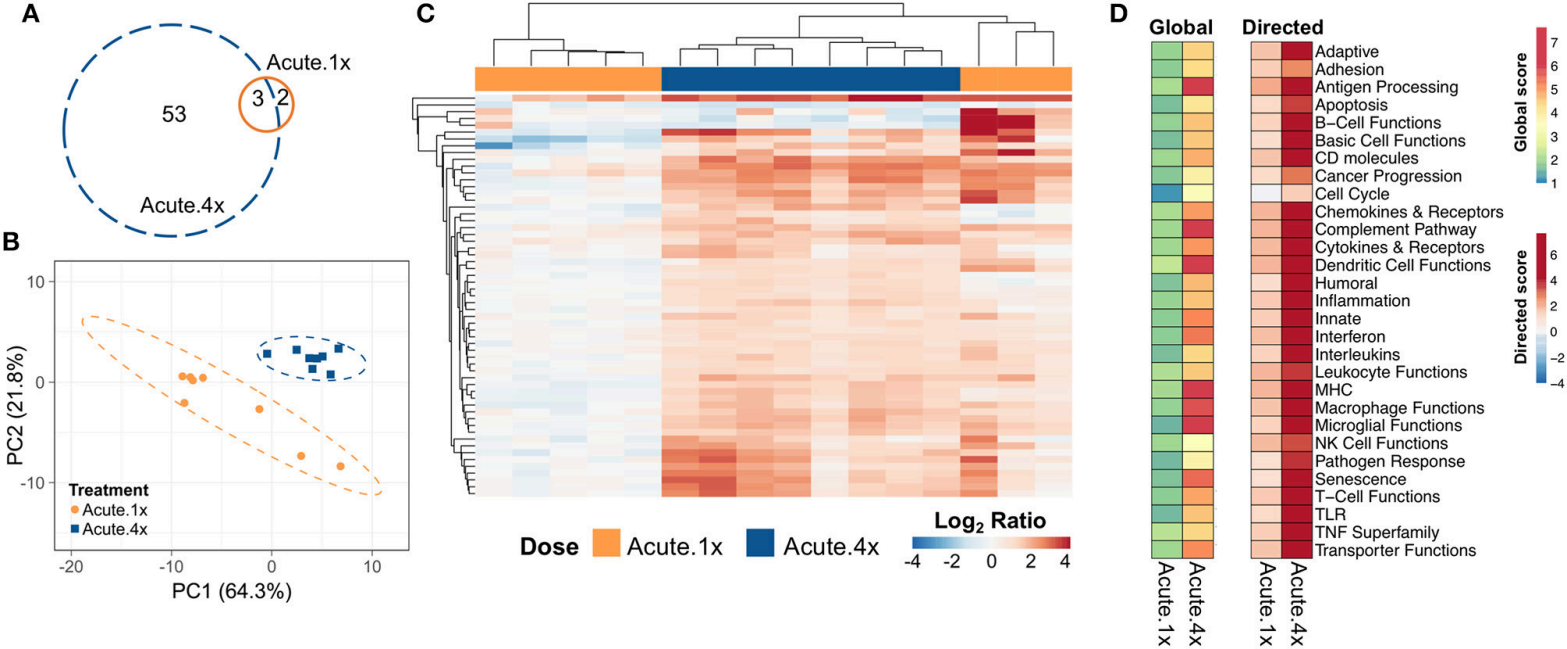
Acute transcriptional response of immune-associated genes in lung tissues of mice that received either a single or four weekly instillations of cSiO_2_. Mice received a single dose (Acute.1x) or four repeated weekly doses (Acute.4x) of cSiO_2_ via intranasal instillation, and gene expression was determined in lung tissues obtained 1 day after the last dose. **(A)** Venn diagram depicting the number of genes identified as differentially expressed in lung tissue of mice that received a single cSiO_2_ instillation (orange line) or four weekly cSiO_2_ doses (blue dashed line) as compared to dosing-matched vehicle controls. **(B)** Principal components analysis of differentially expressed genes (BH *q* < 0.05, log_2_ ratio >1 or < −1) in lung tissues of mice exposed to cSiO_2_ once or four times compared to dosing-matched vehicle controls. PC1 and PC2 are shown with the 95% confidence interval bands (dashed ellipses). **(C)** Unsupervised, bidirectional hierarchical cluster analysis of lung immune pathway transcriptome data using the Euclidean distance method with average linkage. All genes differentially expressed in either the Acute.1x (orange) or Acute.4x (blue) treatment groups were included in the heatmap, which is colored by the log_2_ ratio of transcript counts calculated with respect to average expression in dosing-matched vehicle controls. **(D)** Heatmaps depicting either global or directed significance scores for immune-related pathways (see Materials and Methods for details on calculation of significance scores).

Five genes were differentially expressed in lung tissue 24 h after a single intranasal instillation of cSiO_2_ (Acute.1x) whereas 56 genes were upregulated 24 h after four weekly cSiO_2_ installations (Acute.4x) (*p* < 0.05) (Figure 2A, see Supplementary Tables 1, 2 for a full list of genes). Principal component analysis (PCA) plots (Figure 2B) indicated that distinct gene expression profiles distinguished the immediate immune response to a single exposure of cSiO_2_ as compared to that after repeated cSiO_2_ exposures.

Hierarchical clustering of the 56 genes differentially expressed in either exposure regimen revealed that the changes were more pronounced following four exposures to cSiO_2_ than those elicited by a single exposure to cSiO_2_ (Figure 2C). In general, these changes were associated with increased expression as relatively few genes were repressed. Clustering analysis also indicated that three out of eight mice exposed only once to cSiO_2_ were strong responders whose transcriptomes mirrored the more severe responses observed all eight mice following four repeated cSiO_2_ exposures.

To identify which immunological pathways were significantly altered and were upregulated or enhanced by cSiO_2_ exposure, global and directed significance scores, respectively, were calculated as described above (Figure 2D). The resultant significance scores show broad scale activation of immune pathways. As indicated in heat maps of global and directed significance scores, the involvement of several immune pathways began after a single dose of cSiO_2_ and was amplified upon repeated exposures. Importantly, the vast majority of immune pathways had potentiated expression, as opposed to attenuation, following a single or multiple exposures to cSiO_2_. Network analyses of differentially expressed genes in lungs of mice with multiple cSiO_2_ exposures revealed transcript groupings broadly associated with interferon (IFN) signaling, chemokine and cytokines, innate and adaptive immune response (Supplementary Figure 6).

**Figure 3:**
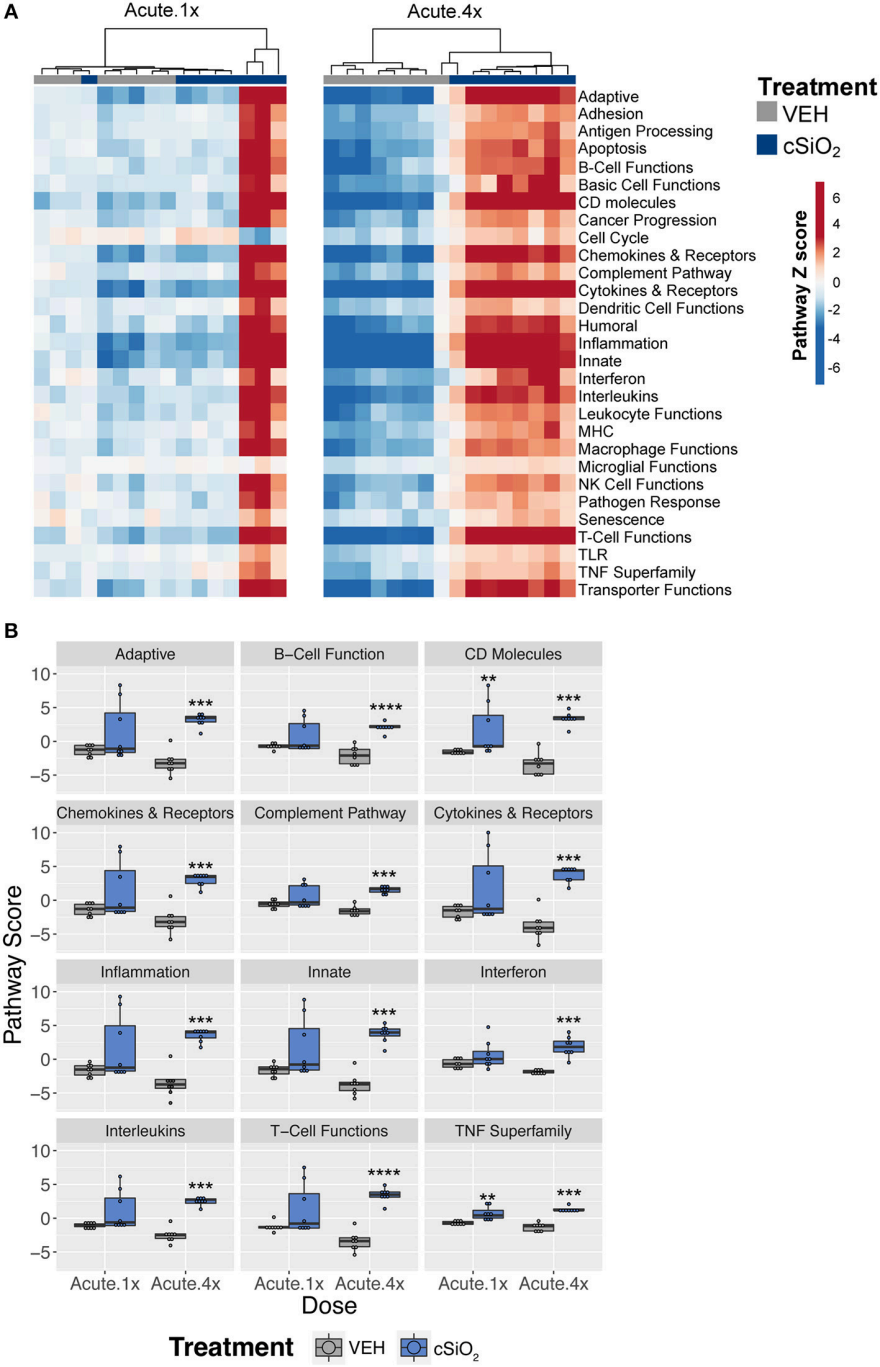
Lung tissue gene expression pathway *Z* scores for mice that received either a single or four weekly instillations of cSiO_2_. **(A)** Heatmap depicts individual pathway *Z* scores of each immune pathway for lung tissues obtained from vehicle (VEH) or cSiO_2_-treated mice following a single (Acute.1x) or four weekly (Acute.4x) instillations. **(B)** Pathway *Z* scores are presented as Tukey box-plots (*n* = 8) for select immune pathways of interest. ^**^p < 0.01; ^***^p < 0.001, and ^****^p < 0.0001 compared to dosing-matched vehicle control as determined by the non-parametric Wilcoxon test.

To ascertain how different exposures to cSiO_2_ influenced the overall gene expression, *Z* scores were calculated using expression values from all genes assigned a given pathway for each mouse in a treatment group, and the data were expressed as heat maps (Figure 3A). Again, strong responders in mice treated with one dose of cSiO_2_ clustered closely and markedly reflected the gene response of mice instilled with multiple doses of cSiO_2_. Importantly, immune pathways consistently activated in cSiO_2_-treated mice were largely parallel with pathways known to influence the development of autoimmunity; these responses were absent in mice treated with vehicle alone (Figure 3B).

**Figure 4:**
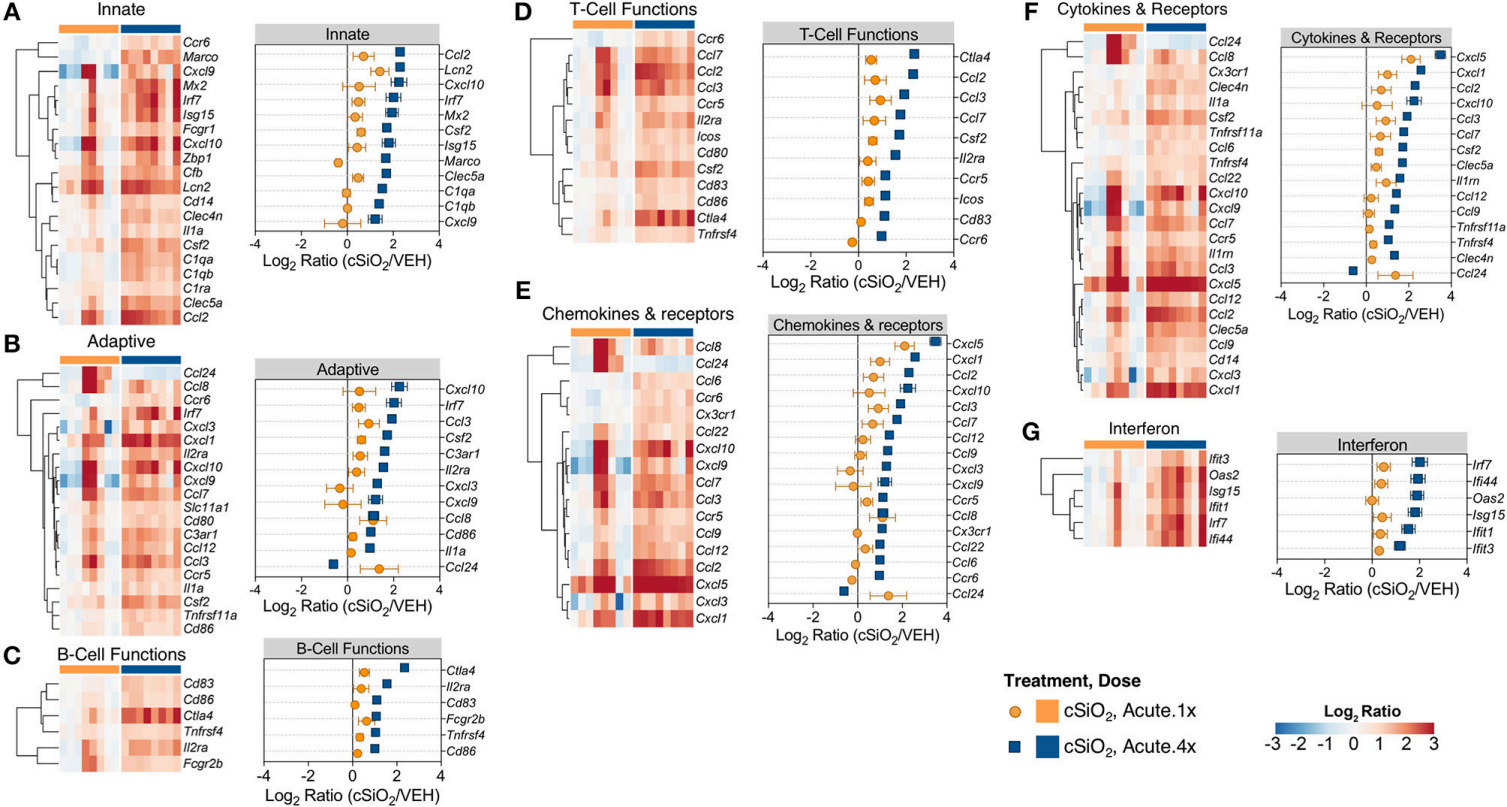
Comparison of significant differentially expressed genes associated with immune response in lung tissues of mice that received either a single or four weekly instillations of cSiO_2_. Gene expression data for **(A)** innate, **(B)** adaptive, **(C)** B-cell functions, **(D)** T-cell functions, **(E)** chemokines & receptors, **(F)** cytokines & receptors, and **(G)** interferon pathways were calculated as the log_2_ ratio of cSiO_2_ with respect to dosing-matched vehicle (VEH) controls for mice that received a single (Acute.1x) or four weekly (Acute.4x) cSiO_2_ instillations. For each pathway of interest, a heatmap with unsupervised clustering (Euclidian distance method) by gene is shown for all genes identified as significantly differentially expressed (BH *q* < 0.05, log_2_ ratio >1 or < −1) for either dosing protocol. Additionally, the mean log_2_ ratio values ± SEM for selected genes of interest are also shown for each panel. Note that some genes are associated with multiple immune response pathways, and thus, these genes appear in multiple panels.

Based on previous studies by our group (15), we chose to elaborate on gene expression profiles with heat maps of the innate immune pathway (Figure 4A), the adaptive immune pathway (Figure 4B), B-cell functions (Figure 4C), T-cell functions (Figure 4D), chemokines and receptors (Figure 4E), cytokines and receptors (Figure 4F), and interferon (Figure 4G). When expression of representative genes of each of these pathways were plotted (Figure 4, dot plots to right of each heat map), upregulation of a number of genes in Acute.4x group were consistent with the Acute.1x high responders. Among the overlapping genes were the chemokines *Cxcl1, Cxcl5, Ccl2, Ccl3*, and *Ccl7* suggesting that leukocyte recruitment was a critical primary response 1 d after cSiO_2_ instillation. Expression of these genes were considerably ramped up after short-term repeated exposure to the particle. Interestingly, a number of genes associated with netosis (26) were also upregulated by cSiO_2_ including *C3ar, Ccl2, Ccl3, Cd14, Cd83, Cxcl, Ifit3, Il1a, Irf7, and Slc11a1* (Figure 4) as well as *Mmp9 and Rsad2* (Supplementary Table 2).

**Figure 5:**
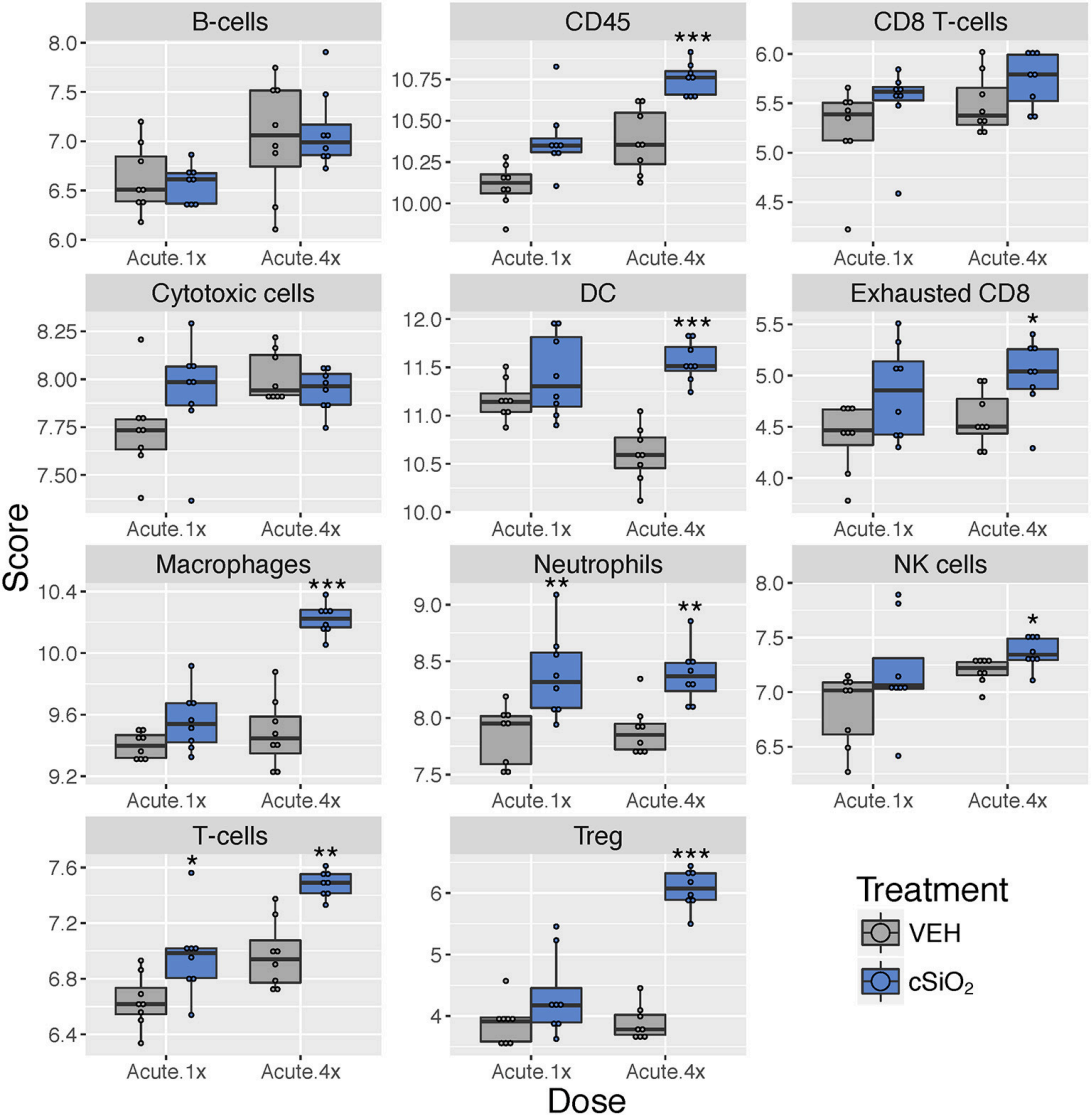
Profiling of immune cell types in lung tissues obtained from mice that received either a single or four weekly instillations of cSiO_2_ compared to non-exposed counterparts. Data shown are the log_2_ cell type scores for mice for mice that received a single (Acute.1x) or four weekly (Acute.4x) instillations of cSiO_2_ or saline vehicle (VEH). Scores may be compared within a cell type by dosing frequency to infer differences in abundance, but comparisons across cell types are not appropriate for this method of quantitation. ^*^p < 0.05; ^**^p < 0.01; and ^***^p < 0.001 compared to dosing-matched vehicle control as determined by the non-parametric Wilcoxon test.

Using marker genes expressed stably and specifically in immune cell types, we estimated relative abundance in sample groups measured as the average log-scale expression of their characteristic genes. Congruent with chemokine effects, several immune cell populations were changed in lung tissue of mice following a single or multiple exposures to cSiO_2_ (Figure 5). Notably, leukocytes, neutrophils, and T-cells were significantly increased following a single exposure to cSiO_2_, indicating a rapid immune response with recruitment of circulating lymphocytes. After multiple exposures, mRNA signatures were suggestive of elevations in cells associated with the innate (macrophages, neutrophils, NK cells) and adaptive immune responses (dendritic cells, B cells, and various classes of T-cells including exhausted CD8 T-cells and Treg cells). Overall, the acute response data following single and short-term repeated exposures were suggestive of a nascent autoimmune response.

### Short-Term Repeated cSiO_2_ Exposure Induces Chronic Changes in Lung mRNA Signatures Consistent With a Vigorous Autoimmune Response

**Figure 6:**
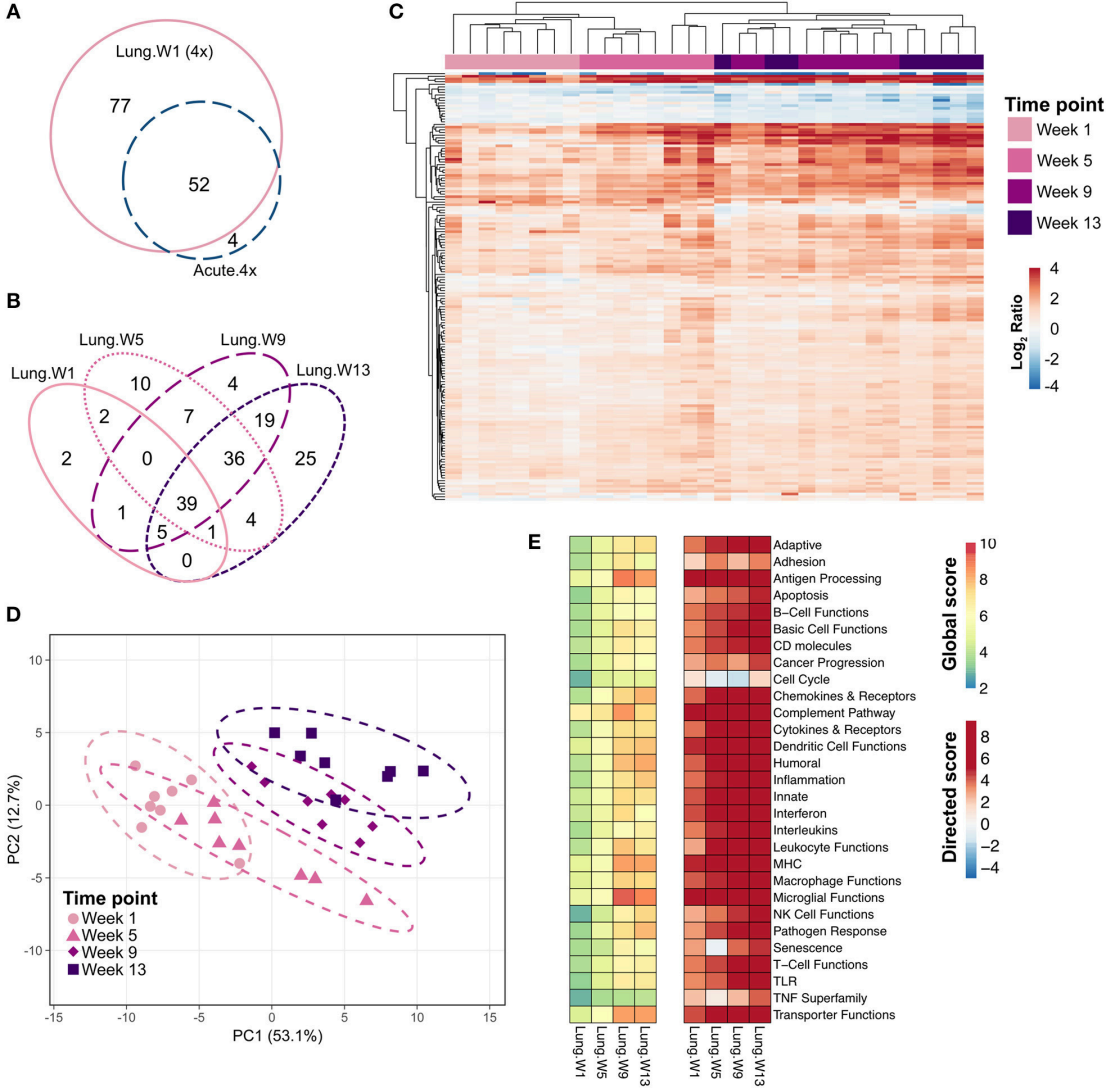
Chronic transcriptional response of immune-associated genes in lung tissues of mice 1, 5, 9, or 13 weeks post cSiO_2_ instillation. Mice received four repeated weekly doses of cSiO_2_ or saline vehicle (VEH) via intranasal instillation, and gene expression was determined in lung tissues obtained 1, 5, 9, or 13 weeks post instillation. **(A)** Venn diagram depicting the number of genes identified as differentially expressed in lung tissue of mice 1 day (Acute.4x) or 1 week (Lung.W1) post cSiO_2_ instillation compared to dosing-matched vehicle controls. **(B)** Venn diagram depicting the number of genes identified as differentially expressed in lung tissues over time as compared to time-matched, vehicle-exposed mice. **(C)** Principal components analysis of differentially expressed genes (BH *q* < 0.05, log_2_ ratio >1 or < −1) in lung tissues of mice exposed to cSiO_2_ at 1, 5, 9, or 13 weeks post instillation compared to time-matched vehicle controls. PC1 and PC2 are shown with 95% confidence interval bands (dashed ellipses). **(D)** Unsupervised, bidirectional hierarchical cluster analysis of lung transcriptome data at weeks 1, 5, 9, or 13 using the Euclidean distance method with average linkage. All genes differentially expressed at any of the time points were included in the heatmap, which is colored by the log_2_ ratio calculated with respect to average expression in time-matched vehicle controls. **(E)** Heatmaps depicting either global or directed significance scores for immune-related pathways (see Materials and Methods for details on calculation of significance scores).

Many of the same genes that were induced in the lung 24 h after the fourth installation of cSiO_2_ were also induced 1 week after the fourth exposure (Figure 6A). The number of differentially expressed genes in the lung increased over time: 77 genes at 1 week, 92 genes at 5 weeks, 110 genes at 9 weeks, and 108 genes at 13 weeks (Figure 6B); again, most of these genes were upregulated with a few repressed (Supplementary Tables 3–6).

PCA plots (Figure 6C) and hierarchical clustering heat maps (Figure 6D) revealed some variance in gene expression profiles over time as evidenced by less distinct clustering by time point, with most variance in individual mice observed at 9 and 13 weeks post exposure. Also, though a core set of 39 genes were commonly differentially regulated by cSiO_2_ at all time points (Figure 6B), both PCA and HCC analyses suggest a shift in overall gene expression profiles over time such that the lung transcriptome during early disease was distinct from that at later disease stages. Global and directed significance scores highlight the dramatic involvement of several immune pathways that appear to be well-established by 5 weeks post final exposure to cSiO_2_ (Figure 6E). Corresponding to 16 weeks of age, this time point may represent a critical transitional step for the establishment of diverse networks of immune pathways. Network visualization of differentially expressed genes in lungs over the course of disease development aligns well with the NanoString pathway analyses, as the resulting networks showed clear groupings of transcripts associated with innate and adaptive immunity, chemokine, cytokines interferon, and complement (Supplementary Figures 7–10).

**Figure 7:**
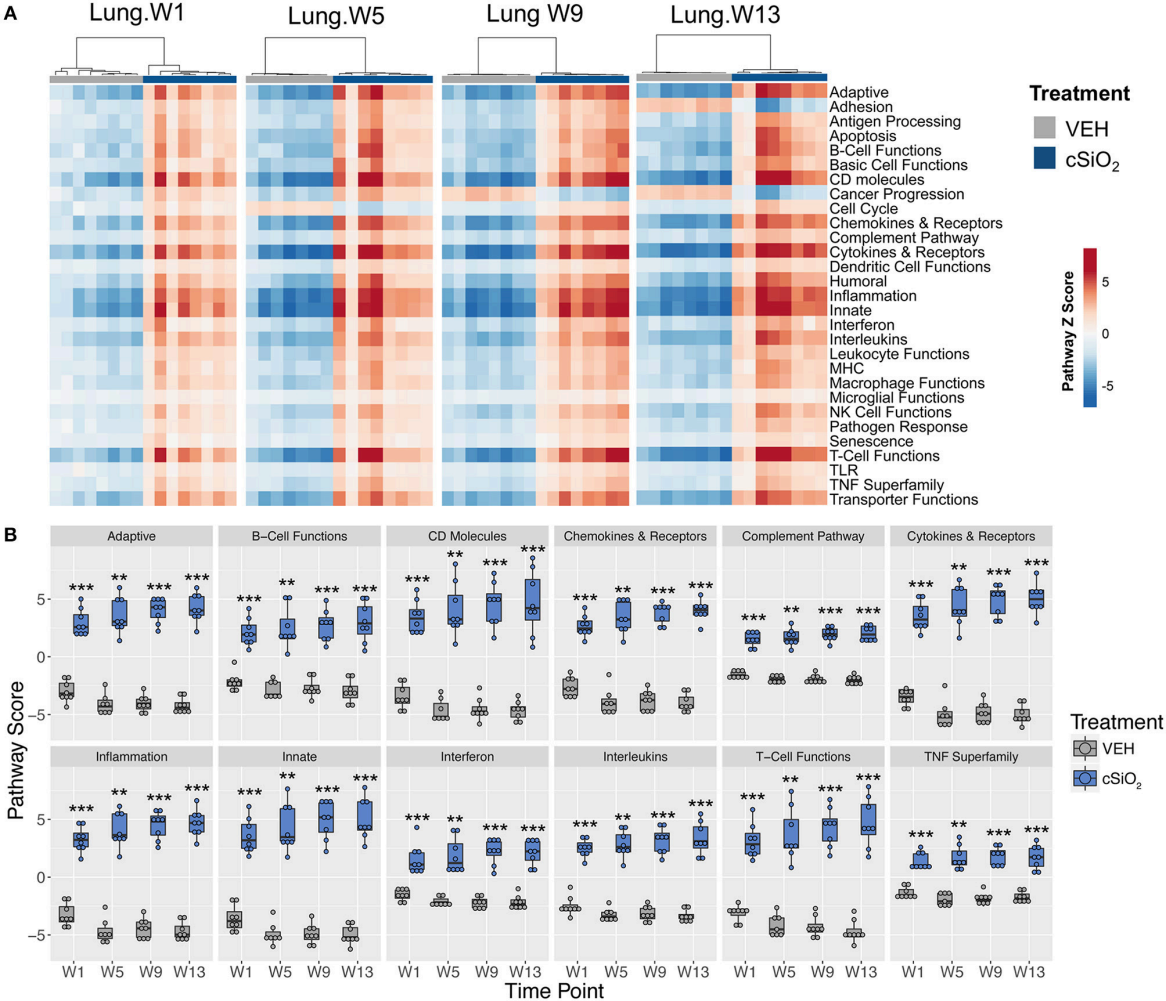
Lung tissue gene expression pathway *Z* scores at 1, 5, 9, or 13 weeks post cSiO_2_ instillation. **(A)** Heatmap depicts individual pathway *Z* scores of each immune pathway for lung tissues obtained from mice 1, 5, 9, or 13 weeks post instillation with cSiO_2_ (four weekly doses) or time-matched vehicle (VEH) controls. **(B)** Pathway *Z* scores are presented as Tukey box-plots (*n* = 7–8) for select immune pathways of interest. ^**^p < 0.01; and ^***^p < 0.001 compared to time-matched vehicle control as determined by the non-parametric Wilcoxon test.

Immune pathways in the lungs of individual mice treated with either cSiO_2_ or VEH clearly segregated into distinct clusters at each time point (Figure 7A). Notably, some pathways were responsive early on and then maintained consistent expression throughout the course of AD development (e.g., interferon, complement pathway, and TNF superfamily) whereas other pathways were modestly induced at early time points but markedly increased with progression of cSiO_2_-induced autoimmunity (e.g., adaptive immunity, B-cell functions and T-cell functions) (Figure 7B).

**Figure 8:**
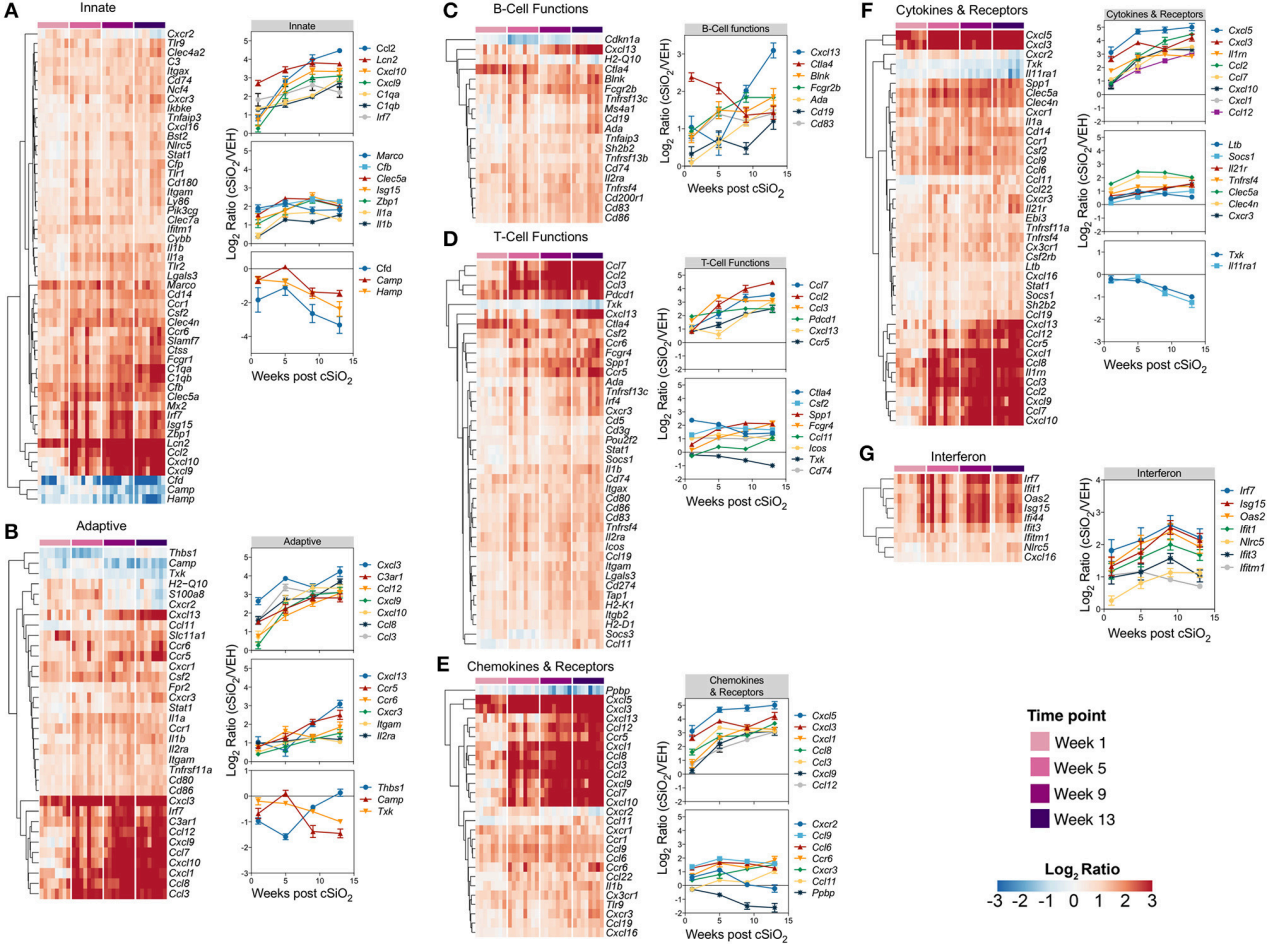
Time course of significant differentially expressed genes associated with immune response in lung tissues of mice 1, 5, 9, or 13 weeks post instillation with cSiO_2_. Gene expression data for **(A)** innate, **(B)** adaptive, **(C)** B-cell functions, **(D)** T-cell functions, **(E)** chemokines & receptors, **(F)** cytokines & receptors, and **(G)** interferon pathways were calculated as the log_2_ ratio of expression values for cSiO_2_-treated mice at 1, 5, 9, or 13 weeks PI with respect to time-matched vehicle controls. For each pathway of interest, a heatmap with unsupervised clustering (Euclidian distance method) by gene is shown for all genes identified as significantly differentially expressed (BH *q* < 0.05, log_2_ ratio >1 or < −1) at any one of the indicated time points. The mean log_2_ ratio values ± SEM for selected genes of interest are also shown for each panel. Note that some genes are associated with multiple immune response pathways, and thus, these genes appear in multiple panels.

Differentially expressed genes identified in immune pathways likely to be involved in the pathogenesis of cSiO_2_-triggered autoimmunity are depicted in heat maps (Figure 8). Expression levels for selected genes representing these immunological pathways are also shown as line plots as a function of time to the right of these heat maps. Consistent with immune pathways, at least three trends were evident for these individual genes. Some genes were upregulated at 1 week post exposure and continued to increase over time (e.g., *Ccl2, Cxcl10, Cxcl13, Cxcl5*). Other sets of genes were modestly elevated at week 1 post exposure and remained so over the course of the experiment (e.g., *Marco, Cfb, Ctla4, Spp1*). Lastly, expression of a small number of genes (e.g., *Camp, Hamp, Txk, Cfd*, and *Il11ra1*) decreased over the length of the study. In addition, sequential comparisons over time were made to identify changes in gene expression as the disease progresses in the cSiO_2_-exposed mice (Supplementary Figure 11). Upon comparing week 5 vs. week 1, week 9 vs. week 5, or week 13 vs. week 9, significant differences in relative expression >2-fold were identified for 17, 12, or 5 genes, respectively.

**Figure 9:**
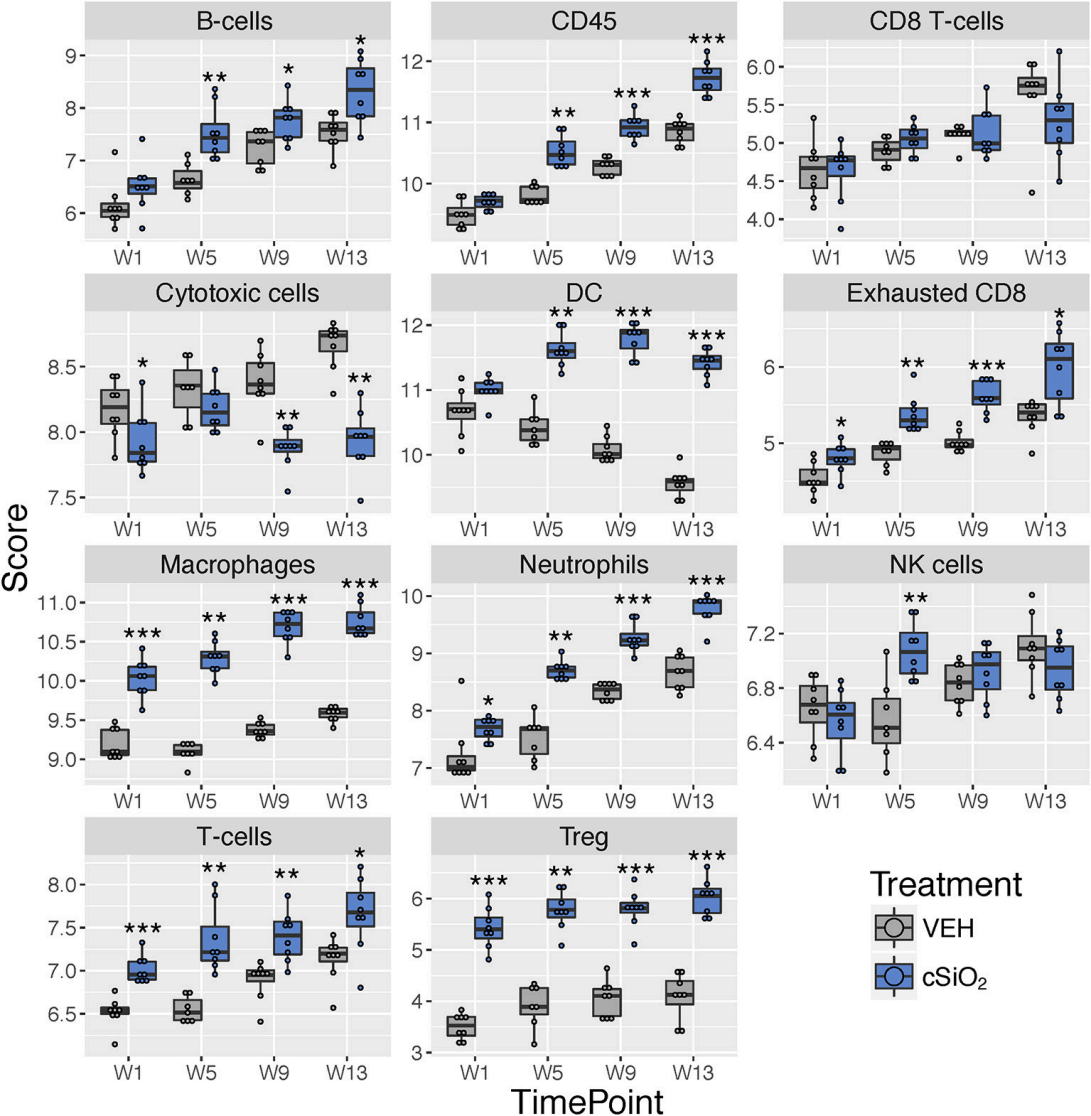
Profiling of selected immune cell types in lung tissues at 1, 5, 9, or 13 weeks post cSiO_2_ instillation. Data shown are the log_2_ cell type scores for lung tissues obtained 1, 5, 9, or 13 weeks following four weekly instillations with cSiO_2_ or saline vehicle (VEH). Scores may be compared within a cell type at each time point to infer differences in abundance, but comparisons across cell types are not appropriate for this method of quantitation. ^*^p < 0.05; ^**^p < 0.01; ^***^p < 0.001 compared to time-matched vehicle control as determined by the non-parametric Wilcoxon test.

Immune cell type profiling in the lung based on expression of cell-specific mRNAs was carried out using NanoString software (Figure 9). Consistent with differential counts of bronchiolar lavage fluid carried out in the parent study (15), both macrophages and neutrophils increased in lungs of cSiO_2_-exposed mice over time. Also, in accordance with our prior finding that cSiO_2_ induced pulmonary ectopic lymphoid neogenesis, B-cell, T-cell, and dendritic cell mRNA signatures were increased in lungs of cSiO_2_-treated mice. Intriguingly, Treg cells and exhausted CD8 T-cells were also increased in cSiO_2_-treated mice.

**Figure 10:**
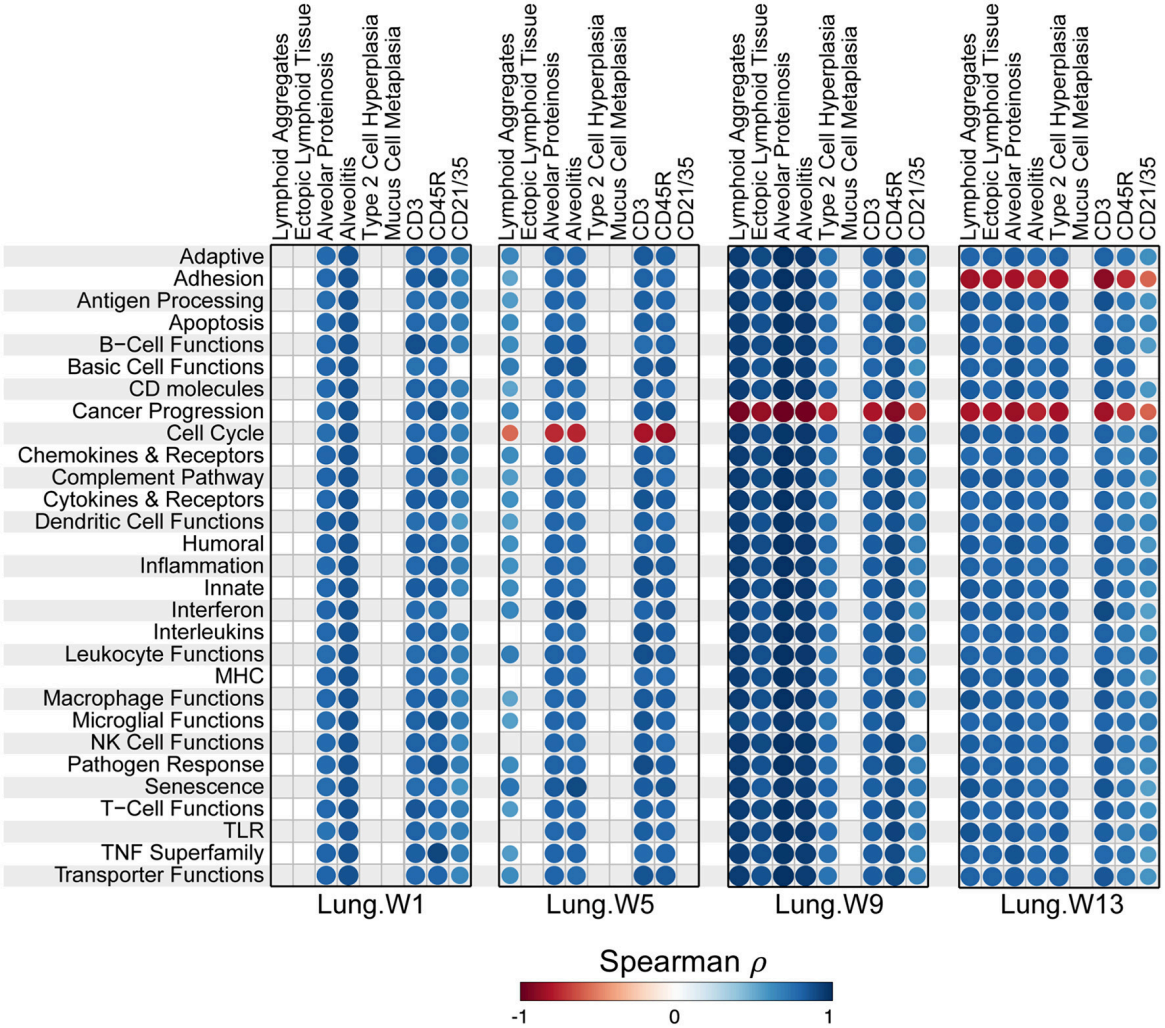
Correlation analyses of immune-associated pathways and phenotypic endpoints assessed in lung tissues of mice collected 1, 5, 9, or 13 weeks post instillation with cSiO_2_. Spearman ρ values were calculated using pathway *Z* scores and phenotype data (histopathology scores (lymphoid aggregates, ectopic lymphoid tissue, alveolar proteinosis, alveolitis, type 2 cell hyperplasia, and mucus cell metaplasia) or percent positive staining tissue (CD3, CD45R, and CD21/35). Significant correlation values (*p* < 0.05) are represented as circles colored by the correlation value (blue, positive; red, negative); non-significant correlations are indicated by blank cells.

Most pathways in individual lungs of cSiO_2_-exposed lupus-prone mice correlated with pathological features the same lung tissues reported in the parent study (15) in a time-dependent manner (Figure 10). Again, the time between weeks 5 and 9 was particularly a critical period for transition.

### Some, but Not All, cSiO_2_-Induced mRNA Signatures in Lung Correlate With Those in Spleen and Kidney

**Figure 11:**
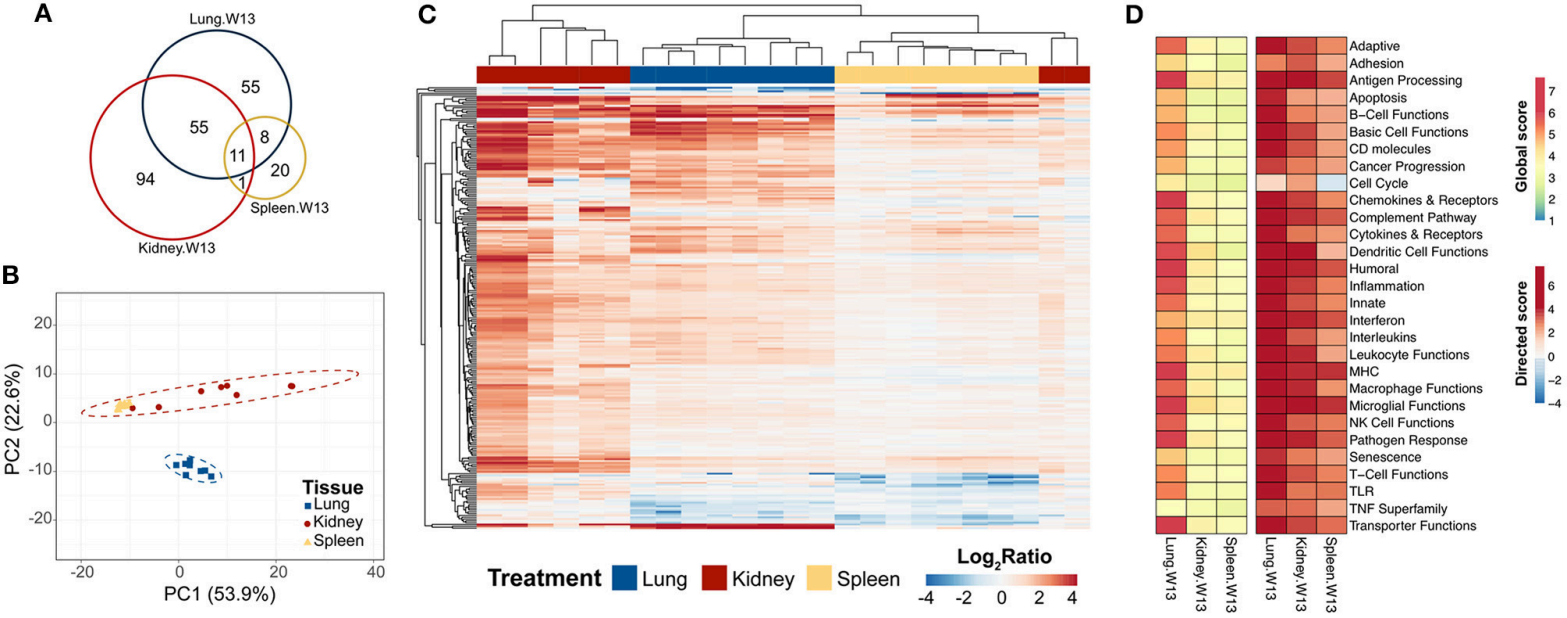
Comparison of chronic transcriptional response of immune-associated genes in lung, kidney and spleen tissues of mice 13 weeks post instillation with cSiO_2._ Mice received four repeated weekly doses of cSiO_2_ or saline vehicle (VEH) via intranasal instillation, and gene expression was determined in lung, kidney and spleen tissues obtained 13 weeks post instillation. **(A)** Venn diagram depicting the number of genes identified as differentially expressed in lung, kidney, and spleen tissues 13 days post installation as compared to time-matched, vehicle-exposed mice. **(B)** Principal components analysis of differentially expressed genes (BH *q* < 0.05, log_2_ ratio >1 or < −1) in lung, kidney, and spleen tissues of cSiO_2_-exposed mice compared to time-matched vehicle controls. PC1 and PC2 are shown with 95% confidence intervals (dashed ellipses). **(C)** Unsupervised, bidirectional hierarchical cluster analysis of lung, kidney, and spleen immune pathway transcriptome data using the Euclidean distance method with average linkage. All genes differentially expressed in any one of the tissue types were included in the heatmap, which is colored by the log_2_ ratio as calculated with respect to average expression in time-matched vehicle controls. **(D)** Heatmaps depicting either global or directed significance scores for immune-related pathways (see Materials and Methods for details on calculation of significance scores).

To learn how the transcriptional responses in the lungs related to other organs associated with the autoimmunity, we compared lung mRNA signatures at 13 weeks after the final instillation of cSiO_2_ to those in the spleen and kidney at the same time point. In spleen, 32 genes were altered and, strikingly, 168 were altered in the kidney (Figure 11A). Importantly, 55 genes were expressed both in the lung and kidney, and 11 genes were common to all tissues at 13 weeks post final exposure (Supplementary Tables 7, 8). PCA indicated the expression profile of the lung clearly segregated from the kidney and spleen, although gene expression profiles for spleen and kidney could not be differentiated by the first two principal components (Figure 11B). Hierarchical clustering clearly demonstrated a substantial immune response in the kidney occurring as a result of intranasal cSiO_2_ exposure, which paralleled closely with genes expressed in the lung and to a lesser extent, the spleen (Figure 11C). Significance scores in the kidney and spleen highlight that, as expected, the lung is the central affected organ at this time point (Figure 11D). However, all tissues have increased involvement of numerous immune pathways, with the kidney having a stronger response than the spleen at 13 weeks post final instillation of cSiO_2_.

**Figure 12:**
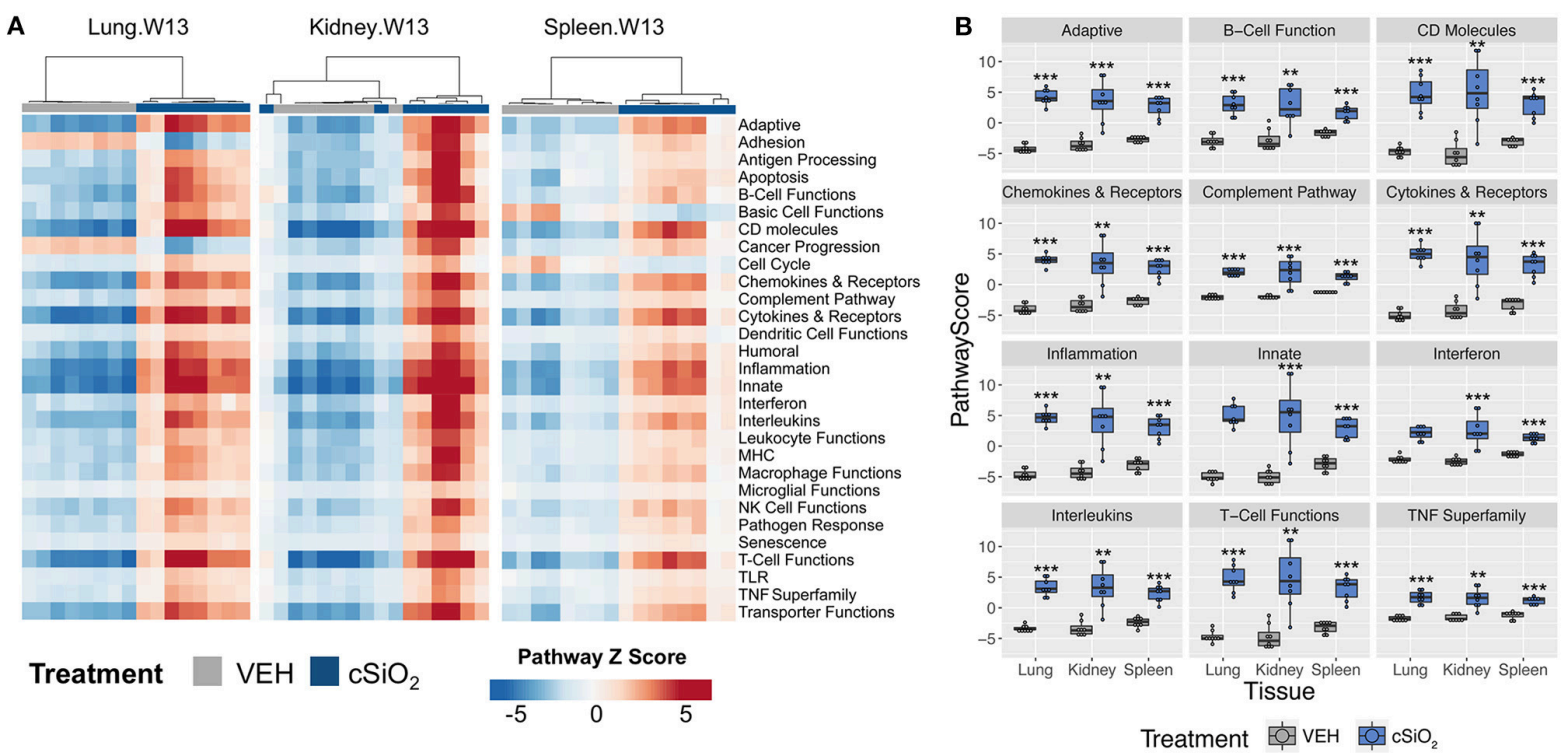
Gene expression pathway *Z* scores for lung, kidney or spleen tissues obtained 13 weeks post cSiO_2_ instillation. **(A)** Heatmap depicts individual pathway *Z* scores of each immune pathway for lung, kidney, or spleen tissues obtained from mice 13 weeks post instillation with cSiO_2_ (four weekly doses) or the corresponding tissue-matched vehicle (VEH) control. **(B)** Pathway *Z* scores are presented as Tukey box-plots (*n* = 8) for select immune pathways of interest. ^**^p < 0.01; and ^***^p < 0.001 compared to time-matched vehicle control as determined by the non-parametric Wilcoxon test.

Further analysis of the immune pathways affected by intranasal cSiO_2_ exposure in the spleen and kidney at 13 weeks reveal paralleled expression of a pathway within tissues of a given animal (Figure 12A). Like the lung, responses within the kidney were quite dramatic, whereas the involvement of the same pathways in the spleen were more modest. Despite the comparatively mild influence of cSiO_2_ on the spleen, pathway scores were significantly increased in the spleen, as well as the lung and kidney, relative to VEH-treated mice (Figure 12B). A similar pattern was evident for the two network visualizations of differentially expressed transcripts in kidney and spleen. For kidney, the network was highly interconnected with major groupings of genes associated with interferon, chemokines and cytokines, TNF signaling, MHC molecules, adhesion, and CD molecules (Supplementary Figure 12). In contrast, the network for cSiO_2_-responsive transcripts in spleen tissues was primarily limited to chemokines and cytokines, with small clusters of three genes associated with interferon or basic cell functions (Supplementary Figure 13).

**Figure 13:**
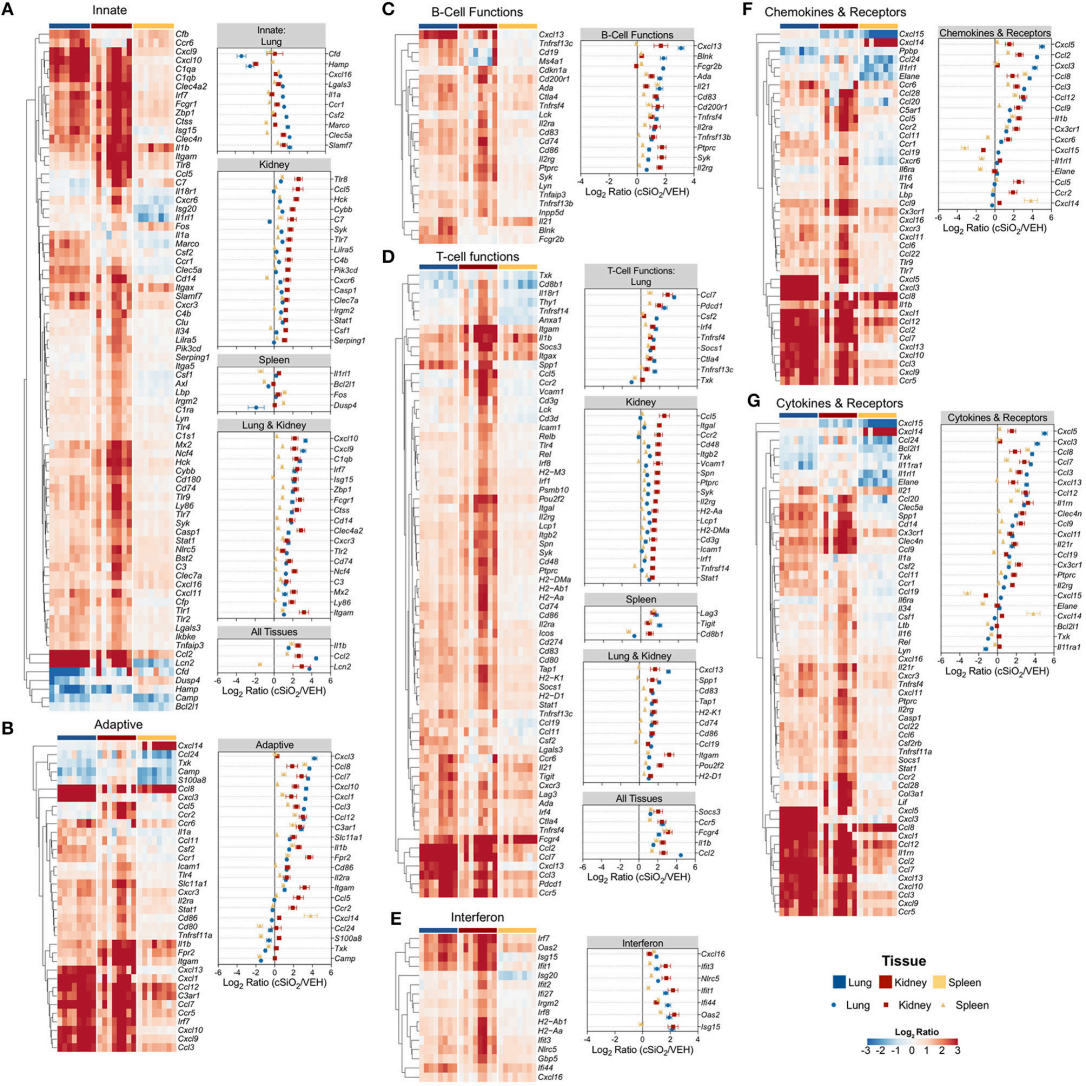
Tissue comparison of significant differentially expressed genes associated with immune response in lung, kidney or spleen of mice 13 weeks post instillation with cSiO_2_. Gene expression data for **(A)** innate, **(B)** adaptive, **(C)** B-cell functions, **(D)** T-cell functions, **(E)** chemokines & receptors, **(F)** cytokines & receptors, and **(G)** interferon pathways were calculated as the log_2_ ratio of expression values for cSiO_2_-treated mice with respect to tissue-matched vehicle controls. For each pathway of interest, a heatmap with unsupervised clustering (Euclidian distance method) is shown for all genes identified as significantly differentially expressed (BH *q* < 0.05, log_2_ ratio >1 or < −1) in any one of the indicated tissues. The mean log_2_ ratio values ± SEM for selected genes of interest are also shown for each panel. Note that some genes are associated with multiple immune response pathways, and thus, these genes appear in multiple panels.

Figure 13 depicts selected tissue-specific gene responses within each immune signaling pathway. It is notable that a large number of genes associated with innate and T cell function were upregulated only in the kidney, such as *Tlr8, Ccl5*, and *Cxcr6* (Figure 13A) or *Itgal, Ccr2*, and *Cd48* (Figure 13D), respectively. In addition, two additional families of mRNA transcripts related to adhesion molecules and collagen deposition were present in the kidneys but not identified in the lungs following cSiO_2_ exposure. The latter correlates with glomerulosclerosis.

**Figure 14:**
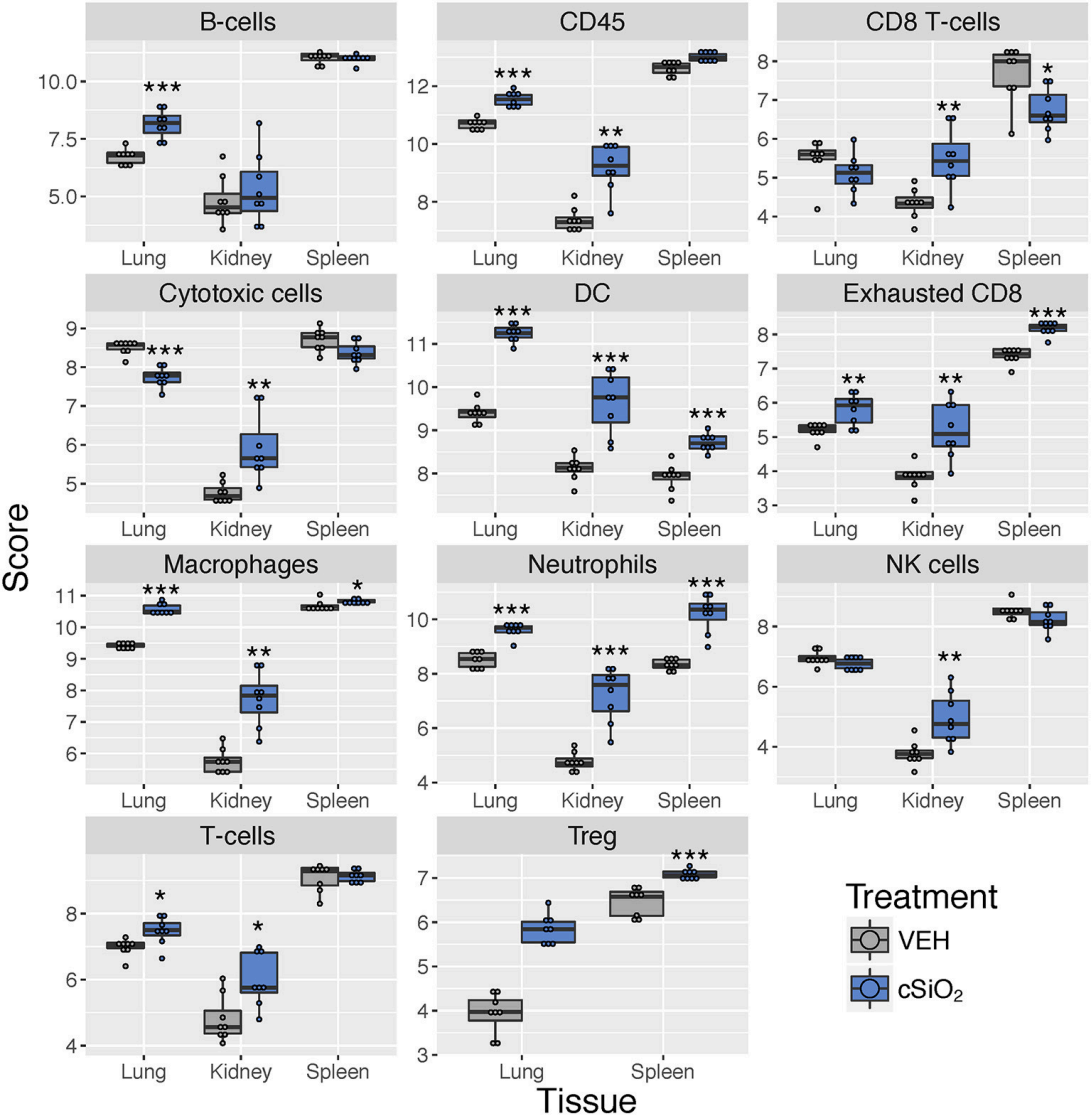
Cell type profiling in lung, kidney and spleen tissues in mice 13 weeks post instillation with cSiO_2_. Data shown are the log_2_ cell type scores for lung, kidney and spleen tissues obtained 13 weeks after four weekly instillations with cSiO_2_ or saline vehicle (VEH). Scores may be compared within a cell type for each tissue to infer differences in abundance, but comparisons across cell types are not appropriate for this method of quantitation. ^*^p < 0.05; ^**^p < 0.01; and ^***^p < 0.001 compared to dosing-matched vehicle control as determined by the non-parametric Wilcoxon test.

Immune cell profiling indicated the involvement of cells of the innate and adaptive immune system in the transcriptional response of the spleen and kidney (Figure 14). As observed in the lung, leukocytes, dendritic cells, macrophages, neutrophils, and exhausted CD8 T-cells had statistically significant increased abundance in the spleen and kidney at 13 weeks post instillation. Additionally, like the lung, T-cells were increased in the kidney. The spleen had fewer cell types that achieved statistical significance possibly due to this tissue having higher basal expression of these markers which masked subtler effects of cSiO_2_ in this tissue.

## Discussion

Identifying the critical genes associated with cSiO_2_-triggered autoimmunity is of great importance because this particle has been linked to human AD. Furthermore, this response may model those of other exogenous particles (e.g., asbestos, carbon nanotubes) as well as endogenous particles (e.g., monosodium urate, cholesterol) (27). Targeted multiplex gene expression analysis with the nCounter provided a focused strategy to test the hypothesis that upregulation of adaptive immune function genes in the lung precedes cSiO_2_-triggering in the NZBWF1 mouse. We found that cSiO_2_ indeed induced wide scale adaptive immune gene upregulation and furthermore, these responses were intricately linked the particle's capacity to elicit an early and persistent inflammatory stimulus in the lung that promoted expression of mRNAs indicative both innate and adaptive immune pathways. Several novel findings were made in this investigation. First, relevant to lupus, dramatic increases were observed in mRNAs associated with chemokine release, cytokine production, sustained IFN activity, complement activation, and adhesion molecule expression. Second, consistent with previously described histopathologic stages of cSiO_2_-triggered autoimmune pathogenesis (15), the time between 5 and 9 weeks PI was an important transition period for gene upregulation in the lung. Third, at experiment termination, cSiO_2_-induced changes in transcriptome signatures were equally robust in kidney but more modest in spleen. Finally, transcriptomic signatures in lung and kidney were indicative of expansion and/or activation of several leukocyte populations including neutrophils, macrophages, dendritic cells, B cells, and T cells that corresponded with accelerated autoimmune pathogenesis.

Both single and multiple cSiO_2_ exposure protocols were employed here to map time-dependent changes in transcriptome signatures in the lung following cSiO_2_ exposure. Regarding acute responses, mRNA signatures 24 h after a single dose of cSiO_2_ (i.e., Acute.1x) were variable with three out of eight mice being extremely high responders and the remainder being low responders. Remarkably, signatures among these high responders were highly consistent with the less variable responses observed 24 h after short term repeated dosing (Acute.4x group). Variability in the single dose acute study might be attributable to use of intranasal instillation as the delivery route for cSiO_2_. Particle suspensions deposited at the anterior nares can distribute into three sites in the mouse: (i) the upper respiratory tract, (ii) the lower respiratory tract, and (iii) the digestive system (28). Thus, variability might have resulted from slow and/or incomplete dissemination of the cSiO_2_ particles in the lower airways of some mice at 1 d following a single dose (29). Comparatively, cSiO_2_ dissemination might be more uniform after four dosages and longer time period than was achieved 24 h after a single installation.

Relative to short-term repeated exposures to cSiO_2_, transcriptome signatures in the lung broadly reflected both activation of innate and adaptive pathways. While similar genes associated with disease pathways were significantly upregulated 1 d and 1, 5, 9, and 13 weeks after the final cSiO_2_ instillation, both the numbers of genes and response intensity progressively increased. Notably, PCA analysis indicated that the most critical breakpoint in expression existed between weeks 5 and 9. This breakpoint corresponded to a massive expansion of ELS and appearance of follicular dendritic and plasma cells in the lung observed immunohistochemically at week 9 in the parent study, the source of the samples used here for the chronic study (15).

Once cSiO_2_ enters the lung, it is largely retained at this site with miniscule amounts translocating to the mediastinal lymph node and thymus (30, 31). Thus, rather than being a direct effect of the particle, cSiO_2_-driven gene responses observed in the kidney are more likely driven by autoantibodies arising in the lung. These autoantibodies likely enter into the systemic compartment in the unbound form or as immune complexes with autoantigens and subsequently deposit in distal tissues—most notably kidney. There they could elicit robust inflammatory responses resulting in glomerulonephritis. Thus, the transcriptome signatures observed in the kidney most likely reflect downstream effects of the ectopic lymphoid neogenesis in the lung.

Diverse chemokine genes were consistently upregulated in lung at all time points following cSiO_2_ exposure. Chemokines are released from injured cells to recruit resident and circulating leukocytes to sites of inflammation by way of concentration gradients. Both C-X-C and C-C chemokines were identified in lung, kidney, and to a lesser extent spleen, thus implicating neutrophils, macrophages, B-cells, and T-cells in the progression of cSiO_2_-triggered autoimmunity. These findings were highly consistent with immune cell profiles both identified using NanoString (Figures 5, 9, 14) and by differential staining of BALF and immunohistochemistry in the parent study (15). *Cxcl5* and *Cxcl3* were consistently among the most highly upregulated genes following cSiO_2_ treatment. These chemokines predominately recruit neutrophils to sites of inflammation via the receptor Cxcr2. Degranulation of recruited neutrophils may release proteolytic enzymes into the extracellular space, thus resulting in bystander tissue injury and promoting lung inflammation (32–34). In addition, neutrophils at sites of inflammation can die by NETosis, a form of regulated cell death that releases neutrophil extracellular traps (NETs) consisting of nuclear material (e.g., dsDNA) that could be a source of autoantigen (35).

Other chemokines identified in this study were consistent with monocyte recruitment into inflamed tissues. These include *Ccl2, Ccl7, Ccl8*, and *Ccl12* which code for monocyte chemoattractant protein 1 (MCP-1), MCP-2, MCP-3, and MCP-5, respectively. In prior work, we have reported that cSiO_2_ exposure promotes MCP-1 elevation in BALF and plasma (13). Importantly, increased MCP-1 in plasma is associated with increased disease severity in lupus patients (36, 37).

C-X-C motif chemokines that are chemoattractant for B and T cells were also found to be persistently upregulated by cSiO_2_ in lung, spleen, and kidney. Of particular interest here, *Cxcl13* was highly expressed in the lung and kidneys of cSiO_2_-treated mice. This chemokine, also known as B-lymphocyte chemoattractant (BLC), is preferentially produced by FDCs in B-cell follicles of lymphoid organs (38) and to some extent, by T follicular helper cells (39, 40) and Th17 cells (41). Relatedly, experimental anti-CXCL13 antibodies have shown promising results on murine models of autoimmune disease (42).

Chemokines that recruit and direct the position of activated T-cells, namely *Cxcl9, Cxcl10, Cxcl11*, and *Cxcl16*, and receptors for these chemokines, *Cxcr3* and *Cxcr6*, were also highly expressed in the lungs of cSiO_2_-exposed mice with similar effects being observed in the kidney. Notably, immune cell profiling revealed that both exhausted CD8 (lung, kidney, spleen) and regulatory T cells (lung, spleen) were upregulated in mice treated with cSiO_2_ (Figures 9, 14). These T cell phenotypes are associated with downregulation of the immune response and increased numbers have been associated with lupus and other autoimmune diseases (43, 44). Thus, their increased numbers observed here following cSiO_2_ exposure likely reflect compensatory mechanisms to limit autoimmune pathogenesis.

Genes activated by the Type I and type II IFN constitute the “IFN signature,” which is evident in 65% of patients with lupus and which correlates strongly with disease severity (45, 46). The panel of genes used to define the IFN signature varies by laboratory; however, we observed striking overlap in the IFN signature described by Li et al. (47). Type I IFNs (i.e., IFN-α and IFN-β) have been established to be pathogenic in lupus [reviewed in Crow (48)]. The main cellular source of Type I IFN is plasmacytoid dendritic cells, which are dependent on IRF4 activation of TLR7/9 (49, 50). Interestingly, NanoString immune cell profiling indicated that increased dendritic cells were evident in lungs, kidneys, and spleens of cSiO_2_-treated mice. Additionally, the aforementioned genes were upregulated in lung (*Irf4*) and kidney (*Tlr7/9*) following cSiO_2_ exposure. Both have been widely implicated in autoimmunity (50) and, of particular relevance here, in ELS during pristane-induced autoimmunity (51). Finally, exogenous administration of IFN-α to NZBWF1 mice accelerated disease onset (52, 53) and decreased the efficacy of pharmacological interventions (54). Type II IFN (i.e., IFN-γ) has also been implicated in lupus (55–57). Secreted predominately by NK and T-cells, IFN-γ heightened adaptive immunity by increased antigen presentation through enhanced expression of MHC II molecules (58) and immunoglobulin production (57). Exogenous administration of IFN-γ to lupus-prone NZBWF1 mice accelerated glomerulonephritis (59); whereas IFN-γ receptor deletion attenuated renal injury and autoantibody generation (52). Collectively, our findings are consistent with a possible pathogenic role for type I and/or type II IFN in cSiO_2_-triggered autoimmunity.

The IL-1 superfamily is a key component of the innate immune system and functions to rapidly initiate inflammatory responses following toxic stimuli via several mechanisms. Consistent with the known actions of cSiO_2_ and other particles [reviewed in Sayan and Mossman (60)], *Il1a, Il1b*, the decoy receptor *Il1r2*, and the receptor antagonist, *Il1rn*, were upregulated by exposure to cSiO_2_ in lungs of NZBWF1 mice. Dying cells release IL-1α, which serves as an alarmin to alert the immune system of an inflammatory stimulus. Cells dying by pyroptosis in an NLRP3 inflammasome-dependent manner release IL-1β by way of caspase-1 activation. Caspase-1, IL-1 β and interleukin 1 receptor associated kinase 3 (IRAK3) were upregulated in the kidneys of cSiO_2_-treated NZBWF1 mice in parallel with IL-1β in this study. Consistent with our findings, aberrant IL-1 signaling and inflammasome activation is suspected to contribute to the pathogenesis of ADs such as lupus [reviewed in Kahlenberg and Kaplan (61)].

Other genes for cytokines and cytokine receptors that potentially mediate formation and maintenance of lymphoid tissues were upregulated in lungs, spleen and kidney of cSiO_2_-treated mice. These included *Lta* (lymphotoxin-α), *Il21, Il21r, Il6, Tnfsf11* (RANKL), *Tnfrsf11a* (RANK or TRANCE). Lymphotoxin-α, otherwise known as TNF-β, is induced by IL-21 and is critical to formation of lymphoid tissues (62). Mice deficient in lymphotoxin-α or RANKL fail to properly develop lymph nodes and Peyer's patches (63, 64). IL-21/IL-21R blockade is associated with decreased IL-6 and autoantibody production and has shown positive effects in preclinical trials in murine models of lupus and rheumatoid arthritis (65, 66).

Upregulation of genes for adhesion molecules that facilitate cellular infiltration are commonly observed in renal biopsies from individuals with lupus nephritis (67–69). Interestingly, expression of genes coding for adhesion were elevated in lupus-prone mice. Specifically, a number of adhesion molecule genes upregulated in the kidney by cSiO_2_ exposure are expressed by infiltrating lymphocytes (*Itga4, Itgal*) and by endothelial cells (e.g., *Sele, Selplg, Vcam1, Icam1*). These might collectively promote cellular infiltration and facilitate enhanced development of glomerulonephritis in cSiO_2_-treated NZBWF1 mice. It should be noted that ELS were negatively associated with adhesion molecule expression at 13 weeks post instillation (15).

Interestingly, cell cycle gene expression was negatively associated with features of ELS, notably lymphoid aggregates containing CD3^+^ and CD45R^+^ cells, at 5 weeks post instillation (Figure 10). Ectopic lymphoid neogenesis might have suppressed active cell proliferation until additional stimuli trigger their re-activation. In addition, genes associated with cancer progression (e.g., *Vegfr2, Angpt2*, and *Kdr*) were suppressed at 9 and 13 weeks post instillation. This may reflect the canon of immune surveillance and disease development: loss of immune tolerance results in autoimmunity, whereas overt immune tolerance results in cancer progression. In this case, active loss of tolerance (as occurs in our model of cSiO_2_-triggered lupus) would effectively suppress pathways key to cancer progression.

cSiO_2_ instillation induced large changes in immune genes in the lung and kidney, whereas responses in the spleen were relatively modest. Lung and kidney typically do not contain many immune cells, and the majority of cells that do persist within these tissues during inflammation are activated. Conversely, the spleen from these mice contain many more non-activated cells compared to the other two tissues which may dilute out the expression of inflammatory genes. Hence, one limitation of comparing differences in cell-specific genes among the tissues is that differences may be skewed due to drastic alterations in the number of activated to non-activated cells within each tissue.

Another limitation of this investigation is that targeted multiplex approaches, such as NanoString, focus on a subset of genes and, thus, do not capture genome-wide changes in mRNA abundance (20). As a result, analytical approaches designed for discovery, such as gene ontology, have limited utility in the identification of novel enriched genes. However, since we focused on immune pathways, a targeted approach was appropriate, as this method improves statistical power by querying a substantially smaller gene set. In addition, we recognize that transcriptomic profiling in whole tissue homogenates restricts the interpretation of results because mRNA signatures cannot be selectively attributed to a given cell type. Nevertheless, the targets discovered herein can be employed as endpoints in future studies that perform mRNA analyses on isolated populations obtained by cell sorting, cell separation columns, or laser dissection of lesions of interest from formalin-fixed paraffin sections (18).

## Conclusion

Taken together, the early and dramatic impact of cSiO_2_ exposure on immune gene expression in the lower respiratory tract promoted the establishment of the lung as the central nexus for launching systemic autoimmunity. Signatures consistent with persistent recruitment of neutrophils, monocytes/macrophages, lymphocytes, and antigen-presenting cells, including dendritic cells, were identified herein, implying that both innate and adaptive immune systems contribute largely to the onset and progression of cSiO_2_-triggered autoimmunity. Future perspectives should include elucidation of the underlying modes of action of cSiO_2_ in alveolar macrophages and neutrophils, two early responders to particles in the lung, as well as identifying novel interventions against this occupational toxicant.

## Data Availability

The data output from nSolver analyses for this study can be found at https://doi.org/10.26078/9vtk-zg12. The raw data supporting the conclusions of this manuscript will be made available by the authors, without undue reservation, to any qualified researcher.

## Author Contributions

MB: study design, animal study coordination, cSiO_2_ exposures, necropsy, RNA analysis, data analyses, interpretation, manuscript preparation, and project funding; AB: data analyses, interpretation, statistical analysis, figure preparation, and manuscript preparation/submission; KG: animal study coordination, RNA analysis, data analyses, and manuscript preparation; AH: experimental design, data interpretation, manuscript writing, and project funding; JH: study design, lung, kidney histopathology, morphometry, data analyses, manuscript preparation, and project funding; JP: planning, coordination, oversight, manuscript preparation, submission, and project funding.

### Conflict of Interest Statement

The authors declare that the research was conducted in the absence of any commercial or financial relationships that could be construed as a potential conflict of interest.

## Supplementary Material

Additional Supplementary Files are available at https://doi.org/10.26078/9vtk-zg12, including the following:

Supplementary File 1 Customized probe annotation file for the Nanostring nCounter Mouse PanCancer Immune Profiling Panel.

Supplementary File 2 Microsoft Excel document with output from nSolver for differential expression analyses.

Supplementary File 3 Microsoft Excel document with output from nSolver for global and directed significance scores for immune pathways.

Supplementary File 4 Microsoft Excel document with output from nSolver for immune pathway *Z* scores for pairwise comparisons.

Supplementary File 5 Microsoft Excel document with output from nSolver for cell type profiling scores.

Supplementary File 6 Microsoft Excel document with protein networks obtained from STRING database and clusters predicted by the MCL algorithm.

## References

[1] Pons-Estel GJ Ugarte-Gil MF Alarcón GS. Epidemiology of systemic lupus erythematosus. Exp Rev Clin Immunol. (2017) 13:799–814. 10.1080/1744666X.2017.1327352 28471259

[2] Nacionales DC Weinstein JS Yan X-J Albesiano E Lee PY Kelly-Scumpia KM. B cell proliferation, somatic hypermutation, class switch recombination, and autoantibody production in ectopic lymphoid tissue in murine lupus. J Immunol. (2009) 182:4226–36. 10.4049/jimmunol.0800771 19299721 PMC3395367

[3] Weinstein JS Nacionales DC Lee PY Kelly-Scumpia KM Yan X-J Scumpia PO. Colocalization of antigen-specific B and T cells within ectopic lymphoid tissue following immunization with exogenous antigen. J Immunol. (2008) 181:3259–67. 10.4049/jimmunol.181.5.3259 18713997 PMC2769209

[4] Jones GW Jones SA. Ectopic lymphoid follicles: inducible centres for generating antigen-specific immune responses within tissues. Immunol. (2016) 147:141–51. 10.1111/imm.12554 26551738 PMC4717241

[5] Gulati G Brunner HI. Environmental triggers in systemic lupus erythematosus. Sem Arthritis Rheum. (2017) 47:710–7. 10.1016/j.semarthrit.2017.10.001 29169635

[6] Parks CG Miller FW Pollard KM Selmi C Germolec D Joyce K. Expert panel workshop consensus statement on the role of the environment in the development of autoimmune disease. Int J Mol Sci. (2014) 15:14269–97. 10.3390/ijms150814269 25196523 PMC4159850

[7] Anonymous Occupational exposure to respirable crystalline silica. Final rule. Fed Regist. (2016) 81:16285–890. Available online at: https://www.govinfo.gov/content/pkg/FR-2016-03-25/pdf/2016-04800.pdf 27017634

[8] Parks CG Cooper GS Nylander-French LA Sanderson WT Dement JM Cohen PL. Occupational exposure to crystalline silica and risk of systemic lupus erythematosus: a population-based, case–control study in the Southeastern United States. Arthritis Rheum. (2002) 46:1840–50. 10.1002/art.10368 12124868

[9] Vupputuri S Parks CG Nylander-French LA Owen-Smith A Hogan SL Sandler DP. Occupational silica exposure and chronic kidney disease. Ren Fail. (2012) 34:40–6. 10.3109/0886022X.2011.623496 22032652 PMC3266824

[10] Cooper GS Parks CG. Occupational and environmental exposures as risk factors for systemic lupus erythematosus. Curr Rheumatol Rep. (2004) 6:367–74. 10.1007/s11926-004-0011-6 15355749

[11] Schleiff PL. Surveillance for silicosis — Michigan and New Jersey, 2003–2011. MMWR Morb Mortal Wkly Rep. (2016) 63:73–8. 10.15585/mmwr.mm6355a7 27736836

[12] Makol A Reilly MJ Rosenman KD. Prevalence of connective tissue disease in silicosis (1985-2006)-a report from the state of Michigan surveillance system for silicosis. Am J Ind Med. (2011) 54:255–62. 10.1002/ajim.20917 20957678

[13] Bates MA Brandenberger C Langohr I Kumagai K Harkema JR Holian A. Silica triggers inflammation and ectopic lymphoid neogenesis in the lungs in parallel with accelerated onset of systemic autoimmunity and glomerulonephritis in the lupus-prone NZBWF1 mouse. PLoS ONE. (2015) 10:e0125481. 10.1371/journal.pone.0125481 25978333 PMC4433215

[14] Bates MA Brandenberger C Langohr II Kumagai K Lock AL Harkema JR. Silica-triggered autoimmunity in lupus-prone mice blocked by docosahexaenoic acid consumption. PLoS ONE. (2016) 11:e0160622. 10.1371/journal.pone.0160622 27513935 PMC4981380

[15] Bates MA Akbari P Gilley KN Wagner JG Li N Kopec AK. Dietary docosahexaenoic acid prevents silica-induced development of pulmonary ectopic germinal centers and glomerulonephritis in the lupus-prone NZBWF1 mouse. Front Immunol. (2018) 9:2002. 10.3389/fimmu.2018.02002 30258439 PMC6143671

[16] Mejia-Vilet JM Parikh SV Song H Fadda P Shapiro JP Ayoub I. Immune gene expression in kidney biopsies of lupus nephritis patients at diagnosis and at renal flare. Nephrol Dial Transplant. (2018). 10.1093/ndt/gfy125. [Epub ahead of print]. 29800348 PMC7967887

[17] Tsang HF Xue VW Koh SP Chiu YM Ng LP Wong SC. NanoString, a novel digital color-coded barcode technology: current and future applications in molecular diagnostics. Expert Rev Mol Diagn. (2017) 17:95–103. 10.1080/14737159.2017.1268533 27917695

[18] Kulkarni MM. Digital multiplexed gene expression analysis using the NanoString nCounter system. Cur Prot Mol Biol. (2011) 25:Unit25B.10. 10.1002/0471142727.mb25b10s94 21472696

[19] Fassbinder-Orth CA. Methods for quantifying gene expression in ecoimmunology: from qPCR to RNA-Seq. Int Comp Biol. (2014) 54:396–406. 10.1093/icb/icu023 24812328

[20] Prokopec SD Watson JD Waggott DM Smith AB Wu AH Okey AB. Systematic evaluation of medium-throughput mRNA abundance platforms. RNA. (2013) 19:51–62. 10.1261/rna.034710.112 23169800 PMC3527726

[21] Reis PP Waldron L Goswami RS Xu W Xuan Y Perez-Ordonez B. mRNA transcript quantification in archival samples using multiplexed, color-coded probes. BMC Biotech. (2011) 11:46. 10.1186/1472-6750-11-46 21549012 PMC3103428

[22] Hulsen T de Vlieg J Alkema W. BioVenn - a web application for the comparison and visualization of biological lists using area-proportional Venn diagrams. BMC Genomics. (2008) 9:488. 10.1186/1471-2164-9-488 18925949 PMC2584113

[23] Oliveros JC Venny. An Interactive Tool for Comparing Lists With Venn's diagrams. (2007-2015). Available online at: http://bioinfogp.cnb.csic.es/tools/venny/index.html.

[24] Metsalu T Vilo J. ClustVis: a web tool for visualizing clustering of multivariate data using Principal Component Analysis and heatmap. Nucleic Acids Res. (2015) 43:W566–70. 10.1093/nar/gkv468 25969447 PMC4489295

[25] Danaher P Warren S Dennis L D'Amico L White A Disis ML. Gene expression markers of tumor infiltrating leukocytes. J Immunther Cancer. (2017) 5:18. 10.1186/s40425-017-0215-8 28239471 PMC5319024

[26] Khan MA Palaniyar N. Transcriptional firing helps to drive NETosis. Sci Rep. (2017) 7:41749. 10.1038/srep41749 28176807 PMC5296899

[27] Nakayama M. Macrophage recognition of crystals and nanoparticles. Front Immunol. (2018) 9:103. 10.3389/fimmu.2018.00103 29434606 PMC5796913

[28] Warawa J. Evaluation of surrogate animal models of melioidosis. Front Microbiol. (2010) 1:141. 10.3389/fmicb.2010.00141 21772830 PMC3109346

[29] Lacher SE Johnson C Jessop F Holian A Migliaccio CT. Murine pulmonary inflammation model: a comparative study of anesthesia and instillation methods. Inhal Toxicol. (2010) 22:77–83. 10.3109/08958370902929969 20017595 PMC4068398

[30] Absher MP Hemenway DR Leslie KO Trombley L Vacek P. Intrathoracic distribution and transport of aerosolized silica in the rat. Exp Lung Res. (1992) 18:743–57. 10.3109/01902149209031705 1327732

[31] Vacek PM Hemenway DR Absher MP Goodwin GD. The translocation of inhaled silicon dioxide: an empirically derived compartmental model. Fund Appl Toxicol. (1991) 17:614–26. 10.1016/0272-0590(91)90211-L 1665463

[32] Korkmaz B Horwitz MS Jenne DE Gauthier F Neutrophil elastase, proteinase 3, and cathepsin G as therapeutic targets in human diseases. Pharmacol Rev. (2010) 62:726–59. 10.1124/pr.110.002733 21079042 PMC2993259

[33] Kruger P Saffarzadeh M Weber ANR Rieber N Radsak M Bernuth Hv. Neutrophils: between host defence, immune modulation, and tissue injury. PLoS Path. (2015) 11:e1004651. 10.1371/journal.ppat.1004651 25764063 PMC4357453

[34] Anderson BO Brown JM Harken AH. Mechanisms of neutrophil-mediated tissue injury. J Surg Res. (1991) 51:170–9. 10.1016/0022-4804(91)90090-9 1650866

[35] Yu Y Su K. Neutrophil extracellular traps and systemic lupus erythematosus. J Clin Cell Immunol. (2013) 4:139. 10.4172/2155-9899.1000139 24244889 PMC3826916

[36] Bauer JW Petri M Batliwalla FM Koeuth T Wilson J Slattery C. Interferon-regulated chemokines as biomarkers of systemic lupus erythematosus disease activity: a validation study. Arthritis Rheum. (2009) 60:3098–107. 10.1002/art.24803 19790071 PMC2842939

[37] El-Shehaby A Darweesh H El-Khatib M Momtaz M Marzouk S El-Shaarawy N. Correlations of urinary biomarkers, TNF-like weak inducer of apoptosis (TWEAK), osteoprotegerin (OPG), monocyte chemoattractant protein-1 (MCP-1), and IL-8 with lupus nephritis. J Clin Immunol. (2011) 31:848–56. 10.1007/s10875-011-9555-1 21691937

[38] Vermi W Lonardi S Bosisio D Uguccioni M Danelon G Pileri S. Identification of CXCL13 as a new marker for follicular dendritic cell sarcoma. J Pathol. (2008) 216:356–64. 10.1002/path.2420 18792075

[39] Chtanova T Tangye SG Newton R Frank N Hodge MR Rolph MS. T follicular helper cells express a distinctive transcriptional profile, reflecting their role as non-Th1/Th2 effector cells that provide help for B cells. J Immunol. (2004) 173:68–78. 10.4049/jimmunol.173.1.68 15210760

[40] Gu-Trantien C Migliori E Buisseret L de Wind A Brohée S Garaud S. CXCL13-producing TFH cells link immune suppression and adaptive memory in human breast cancer. JCI Insight. 2:91487. 10.1172/jci.insight.91487 28570278 PMC5453706

[41] Takagi R Higashi T Hashimoto K Nakano K Mizuno Y Okazaki Y. B cell chemoattractant CXCL13 is preferentially expressed by human Th17 cell clones. J Immunol. (2008) 181:186–9. 10.4049/jimmunol.181.1.186 18566383

[42] Klimatcheva E Pandina T Reilly C Torno S Bussler H Scrivens M. CXCL13 antibody for the treatment of autoimmune disorders. BMC Immunol. (2015) 16:6. 10.1186/s12865-015-0068-1 25879435 PMC4329654

[43] Ohl K Tenbrock K. Regulatory T cells in systemic lupus erythematosus. Eur J Immunol. (2015) 45:344–55. 10.1002/eji.201344280 25378177

[44] McKinney EF Lee JC Jayne DR Lyons PA Smith KG. T-cell exhaustion, co-stimulation and clinical outcome in autoimmunity and infection. Nature. (2015) 523:612–6. 10.1038/nature14468 26123020 PMC4623162

[45] Baechler EC Batliwalla FM Karypis G Gaffney PM Ortmann WA Espe KJ. Interferon-inducible gene expression signature in peripheral blood cells of patients with severe lupus. Proc Natl Acad Sci USA. (2003) 100:2610–5. 10.1073/pnas.0337679100 12604793 PMC151388

[46] Bezalel S Guri KM Elbirt D Asher I Sthoeger ZM. Type I interferon signature in systemic lupus erythematosus. Israel Med Assoc J. (2014) 16:246–9. Available online at: https://www.ima.org.il/FilesUpload/IMAJ/0/77/38682.pdf 24834763

[47] Li Q-Z Zhou J Lian Y Zhang B Branch VK Carr-Johnson F. Interferon signature gene expression is correlated with autoantibody profiles in patients with incomplete lupus syndromes. Clin Exp Immunol. (2010) 159:281–91. 10.1111/j.1365-2249.2009.04057.x 19968664 PMC2819494

[48] Crow MK. Type I interferon in the pathogenesis of lupus. J Immunol. (2014) 192:5459–68. 10.4049/jimmunol.1002795 24907379 PMC4083591

[49] Honda K Yanai H Negishi H Asagiri M Sato M Mizutani T. IRF-7 is the master regulator of type-I interferon-dependent immune responses. Nature. (2005) 434:772–7. 10.1038/nature03464 15800576

[50] Kim J-M Park S-H Kim H-Y Kwok S-K. A plasmacytoid dendritic cells-type I interferon axis is critically implicated in the pathogenesis of systemic lupus erythematosus. Int J Molec Sci. (2015) 16:14158–70. 10.3390/ijms160614158 26110387 PMC4490545

[51] Nacionales DC Kelly KM Lee PY Zhuang H Li Y Weinstein JS. Type I interferon production by tertiary lymphoid tissue developing in response to 2,6,10,14-tetramethyl-pentadecane (pristane). Am J Pathol. (2006) 168:1227–40. 10.2353/ajpath.2006.050125 16565497 PMC1606560

[52] Liu Z Bethunaickan R Huang W Lodhi U Solano I Madaio MP Interferon alpha accelerates murine SLE in a T cell dependent manner. Arthr Rheum. (2011) 63:219–29. 10.1002/art.30087 20954185 PMC3014995

[53] Mathian A Weinberg A Gallegos M Banchereau J Koutouzov S IFN-alpha induces early lethal lupus in preautoimmune (New Zealand Black x New Zealand White) F1 but not in BALB/c mice. J Immunol. (2005) 174:2499–506. 10.4049/jimmunol.174.5.2499 15728455

[54] Liu Z Bethunaickan R Huang W Ramanujam M Madaio MP Davidson A IFNα confers resistance of SLE nephritis to therapy in NZB/WF1 mice. J Immunol. (2011) 187:1506–13. 10.4049/jimmunol.1004142 21705616 PMC3140572

[55] Munroe ME Lu R Zhao YD Fife DA Robertson JM Guthridge JM. Altered type II interferon precedes autoantibody accrual and elevated type I interferon activity prior to systemic lupus erythematosus classification. Ann Rheum Dis. (2016) 75:2014–21. 10.1136/annrheumdis-2015-208140 27088255 PMC4959992

[56] Jackson SW Jacobs HM Arkatkar T Dam EM Scharping NE Kolhatkar NS. B cell IFN-γ receptor signaling promotes autoimmune germinal centers via cell-intrinsic induction of BCL-6. J Exp Med. (2016) 213:733–50. 10.1084/jem.20151724 27069113 PMC4854732

[57] Pollard KM Cauvi DM Toomey CB Morris KV Kono DH. Interferon-γ and systemic autoimmunity. Discovery Med. (2013) 16:123–31. 23998448 PMC3934799

[58] Schoenborn JR Wilson CB. Regulation of interferon-gamma during innate and adaptive immune responses. Adv Immunol. (2007) 96:41–101. 10.1016/S0065-2776(07)96002-2 17981204

[59] Jacob CO van der Meide PH McDevitt HO. *In vivo* treatment of (NZB X NZW)F1 lupus-like nephritis with monoclonal antibody to gamma interferon. J Exp Med. (1987) 166:798–803. 10.1084/jem.166.3.798 3114409 PMC2188698

[60] Sayan M Mossman BT. The NLRP3 inflammasome in pathogenic particle and fibre-associated lung inflammation and diseases. Part Fibre Toxicol. (2016) 13:15. 10.1186/s12989-016-0162-4 27650313 PMC5029018

[61] Kahlenberg JM Kaplan MJ. The inflammasome and lupus: another innate immune mechanism contributing to disease pathogenesis? Cur Opin Rheum. (2014) 26:475–81. 10.1097/BOR.0000000000000088 24992143 PMC4153426

[62] Jang E Cho S-H Park H Paik D-J Kim JM Youn J. A positive feedback loop of IL-21 signaling provoked by homeostatic CD4+CD25– T cell expansion is essential for the development of arthritis in autoimmune K/BxN mice. J Immunol. (2009) 182:4649–56. 10.4049/jimmunol.0804350 19342640

[63] Matsumoto M Fu YX Molina H Chaplin DD. Lymphotoxin-alpha-deficient and TNF receptor-I-deficient mice define developmental and functional characteristics of germinal centers. Immun Rev. (1997) 156:137–44. 10.1111/j.1600-065X.1997.tb00965.x 9176705

[64] Dougall WC Glaccum M Charrier K Rohrbach K Brasel K De Smedt T. RANK is essential for osteoclast and lymph node development. Genes Dev. (1999) 13:2412–24. 10.1101/gad.13.18.2412 10500098 PMC317030

[65] Herber D Brown TP Liang S Young DA Collins M Dunussi-Joannopoulos K. IL-21 has a pathogenic role in a lupus-prone mouse model and its blockade with IL-21R.Fc reduces disease progression. J Immunol. (2007) 178:3822–30. 10.4049/jimmunol.178.6.3822 17339481

[66] Young DA Hegen M Ma HLM Whitters MJ Albert LM Lowe L. Blockade of the interleukin-21/interleukin-21 receptor pathway ameliorates disease in animal models of rheumatoid arthritis. Arthr Rheum. (2007) 56:1152–63. 10.1002/art.22452 17393408

[67] Sabry A Sheashaa H El-Husseini A El-Dahshan K Abdel-Rahim M Elbasyouni SR. Intercellular adhesion molecules in systemic lupus erythematosus patients with lupus nephritis. Clin Rheumatol. (2007) 26:1819–23. 10.1007/s10067-007-0580-7 17340048

[68] Daniel L Sichez H Giorgi R Dussol B Figarella-Branger D Pellissier JF. Tubular lesions and tubular cell adhesion molecules for the prognosis of lupus nephritis. Kidney Int. (2001) 60:2215–21. 10.1046/j.1523-1755.2001.00055.x 11737595

[69] Hauser IA Riess R Hausknecht B Thüringer H Sterzel RB. Expression of cell adhesion molecules in primary renal disease and renal allograft rejection. Nephrol Dial Transplant. (1997) 12:1122–31. 10.1093/ndt/12.6.1122 9198039

