# Supplementary material for: Mapping of Dynamic Transcriptome Changes Associated With Silica-Triggered Autoimmune Pathogenesis in the Lupus-Prone NZBWF1 Mouse
Source: Front Immunol. 2019 Mar 29;10:632. doi: 10.3389/fimmu.2019.00632 (PMC6450439; doi:10.3389/fimmu.2019.00632)
Supplement: Supplementary File 1 — Customized probe annotation file for the Nanostring nCounter Mouse PanCancer Immune Profiling Panel. [file Data_Sheet_1.PDF]

Supplementary Material

Mapping of Dynamic Transcriptome Changes Associated with Silica-Triggered Autoimmune Pathogenesis in the Lupus-Prone NZBWF1 Mouse

Melissa A. Bates, Abby D. Benninghoff*, Kristen N. Gilley, Andrij Holian[4] ,
Jack R. Harkema, and James J. Pestka*

*** Correspondence:**
Abby D. Benninghoff, Department of Animal, Dairy and Veterinary Sciences, Utah State University, 4815 Old Main Hill, Logan, UT 84339, USA. E-Mail: abby.benninghoff@usu.edu

James J. Pestka, Department of Food Science and Human Nutrition, Michigan State University, East Lansing, MI 48824, USA. E-mail: pestka@msu.edu

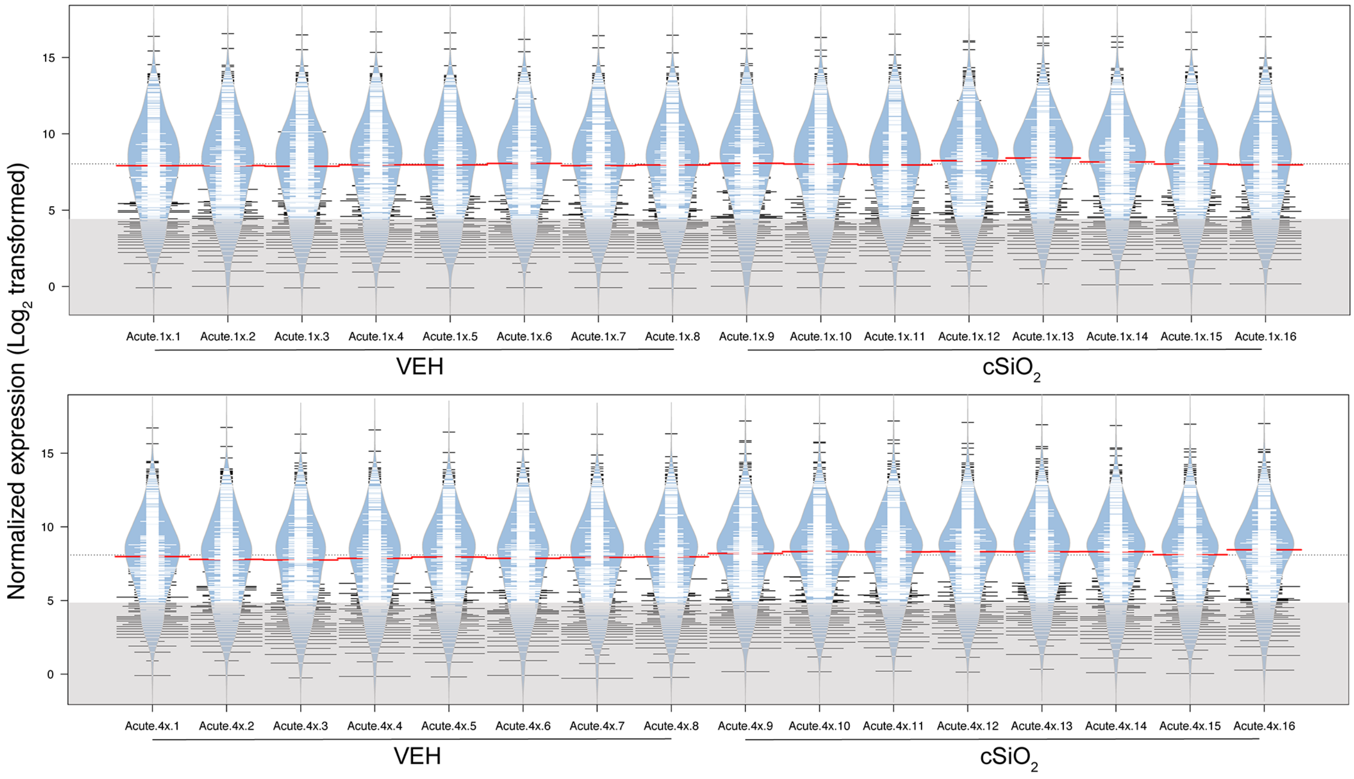

Supplementary figure 1. Beanplots depicting distribution of normalized expression of nanoString PanCancer Immune gene panel – acute response in lung (experiments 1 and 2). Samples are named by dosing regimen (Acute.1x, single instillation; Acute.4x, four weekly instillations) and sample numbers, for which 1-8 correspond to vehicle controls (VEH) and numbers 9-16 correspond to $cSiO_2$-exposed mice. Red lines show the medians, white/black lines represent individual data points (line width indicates number of genes at that expression level), and blue polygons represent the estimated density of the data. The shaded region indicates genes excluded because of low signal. Plots were generated using BoxPlotR (shiny.chemgrid.org/boxplotr/).

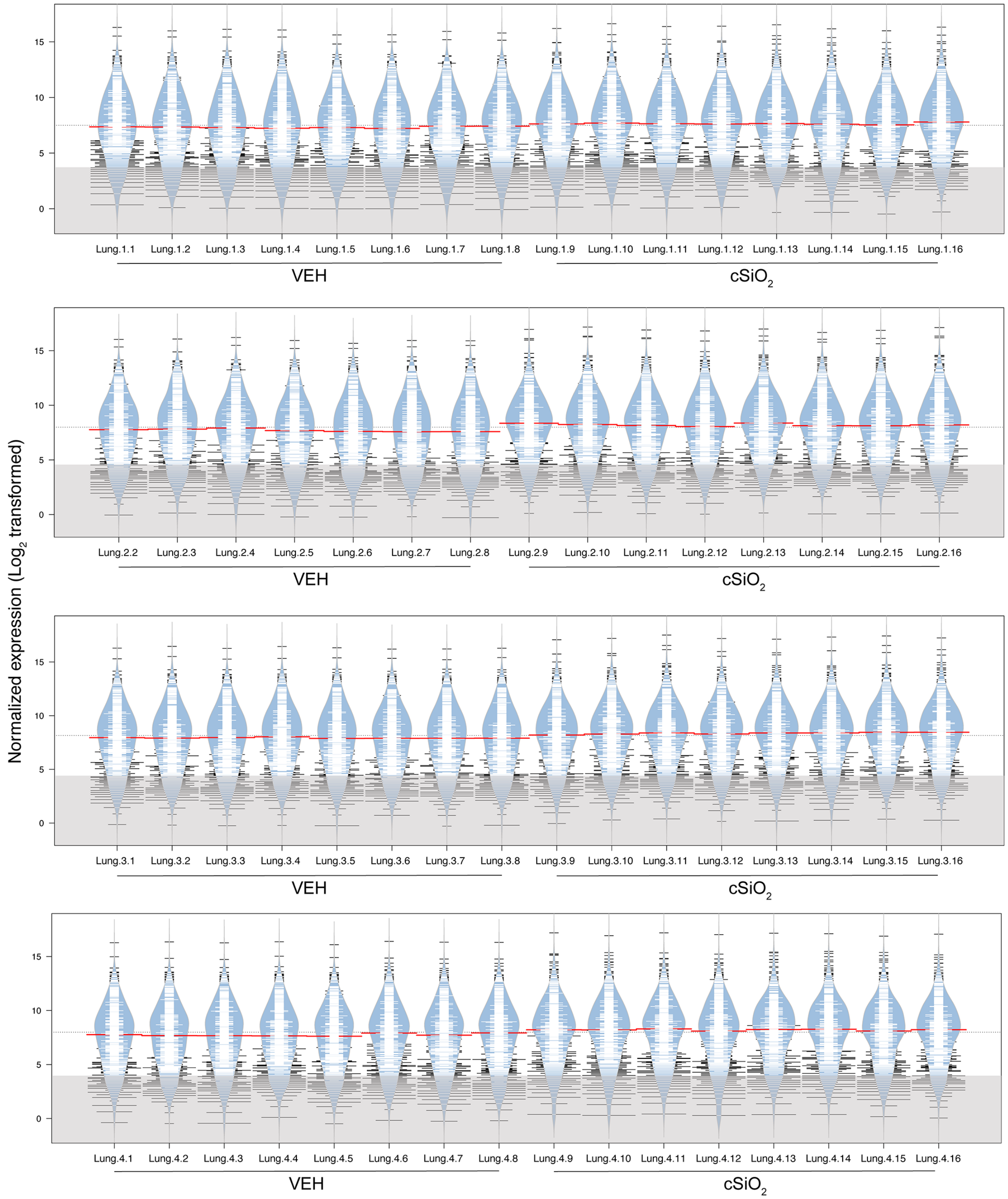

Supplementary figure 2. Beanplots depicting distribution of normalized expression of nanoString PanCancer Immune gene panel – chronic response in lung (experiment 3). Samples are named by "tissue.cohort.ID". Cohorts 1, 2, 3, and 4 correspond to samples obtained 1, 5, 9, or 13 weeks post instillation with $cSiO_2$. Sample ID numbers 1-8 correspond to vehicle controls (VEH), and numbers 9-16 correspond to cSiO2-exposed mice. Red lines show the medians, white/black lines represent individual data points (line width indicates number of genes at that expression level), and blue polygons represent the estimated density of the data. The shaded region indicates genes excluded because of low signal. Plots were generated using BoxPlotR (shiny.chemgrid.org/boxplotr/).

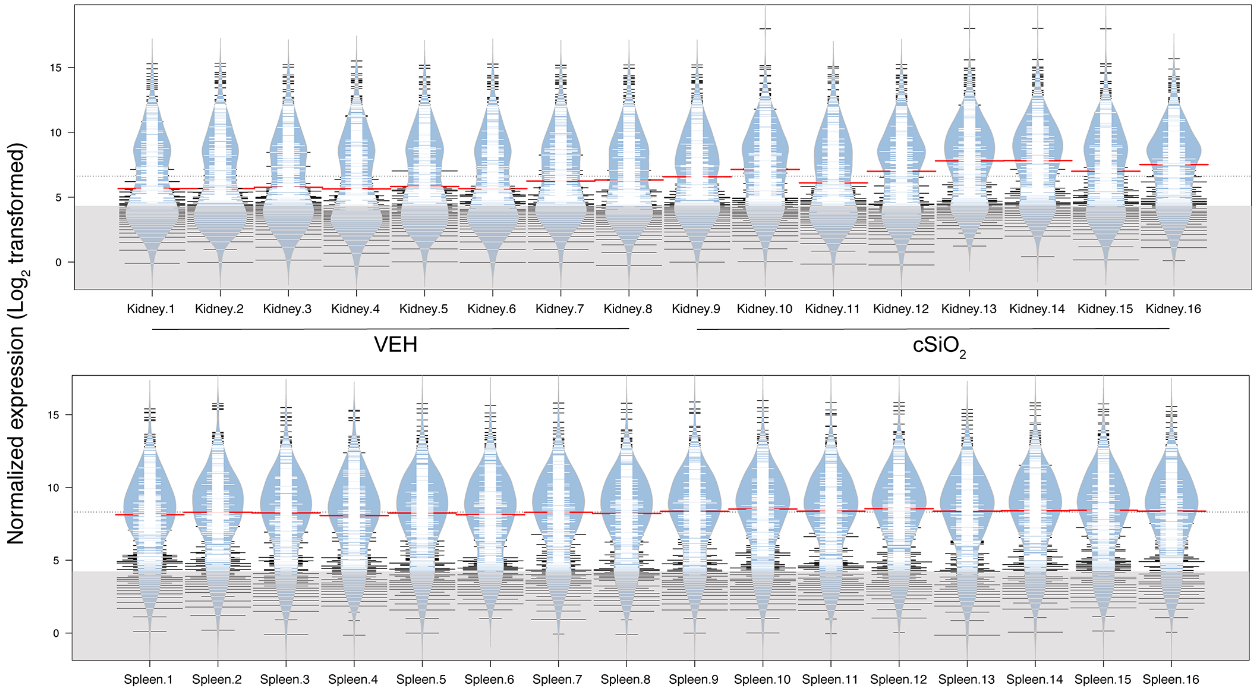

Supplementary figure 3. Beanplots depicting distribution of normalized expression of nanoString PanCancer Immune gene panel – chronic response in kidney and spleen. Samples are named by tissue (kidney or spleen) and sample numbers, for which 1-8 correspond to vehicle controls (VEH) and numbers 9-16 correspond to cSiO2-exposed mice. Red lines show the medians, white/black lines represent individual data points (line width indicates number of genes at that expression level), and blue polygons represent the estimated density of the data. The shaded region indicates genes excluded because of low signal. Plots were generated using BoxPlotR (shiny.chemgrid.org/boxplotr/).

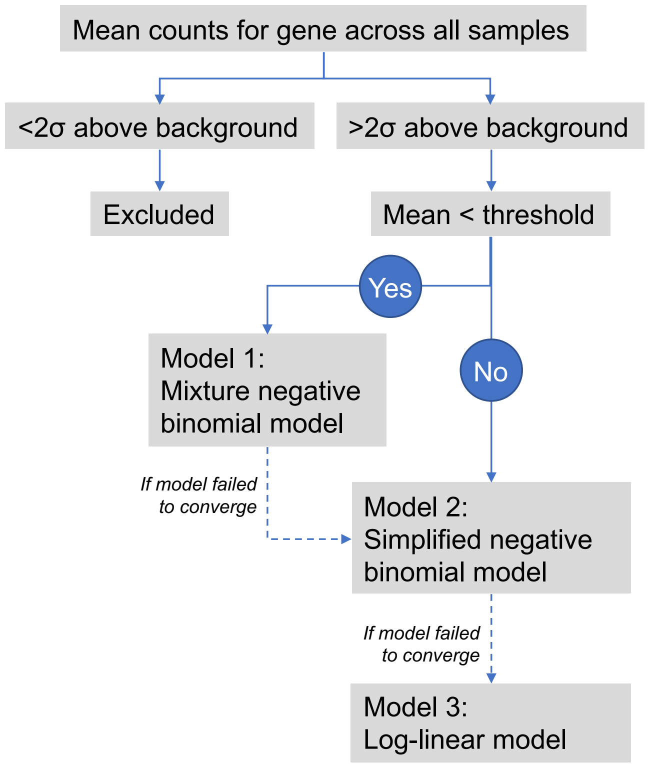

Supplementary figure 4. Process flow for differential gene expression analysis using nSolver. As outlined by the supplied user manual[1], the mean of the gene across all samples was compared against the threshold, which is set as 10 times the background signal. If the gene mean was below the threshold, the mixture negative binomial (*MLE* function in R, Wald test to calculate a *p* value) was applied; if model 1 did not converge, the simplified model in 2 (*glm.nb* function in R/Mass) was applied instead. If the gene mean was above the threshold, the mixture model in 1 was simplified to model 2. If model 2 did not converge, the log-linear model 3 was used (*lm* function in R). For each gene, the optimal model applied for each gene is provided in Supplementary File 2 (model 1, "Wald"; model 2, "lm.nb"; model 3, "loglinear").

[1] NanoString Technologies, Inc. (2018) nCounter Advanced Analysis 2.0 Plugin for nSolver Software User Manual, vers. Jan 2018 (MAN-10030-03). Accessed at www.nanostring.com

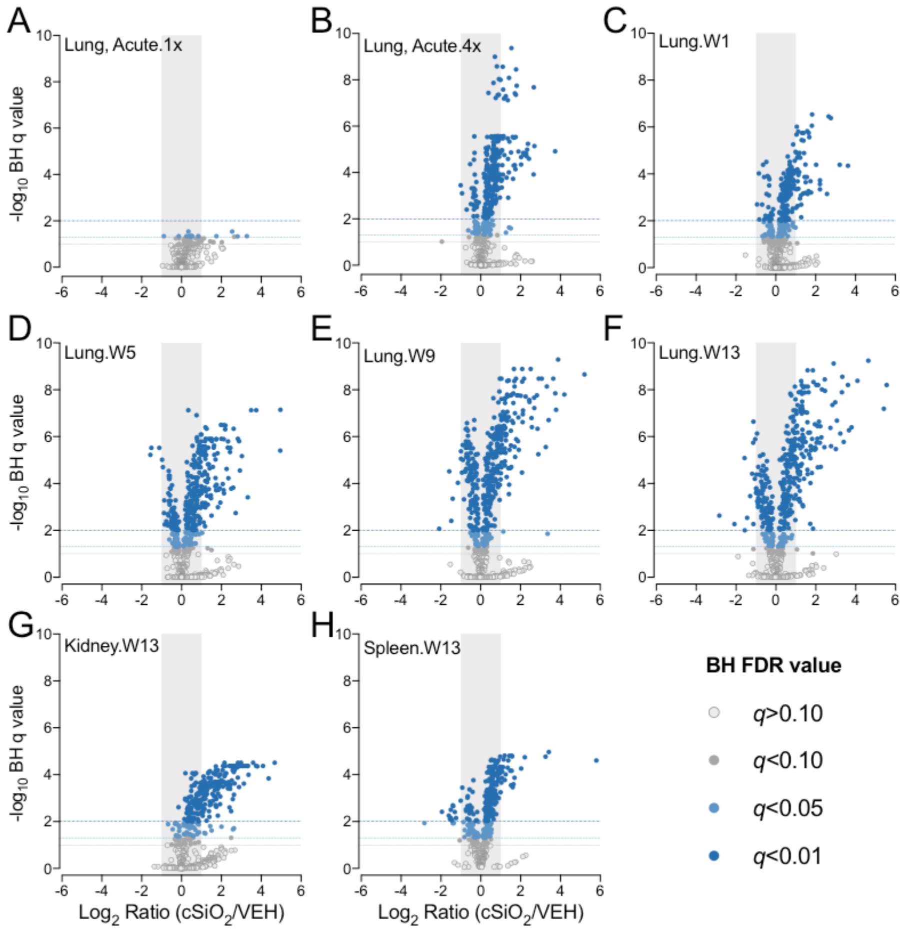

Supplementary figure 5. Volcano plots depicting immune-related mRNA expression as measured using the nanoString Mouse PanCancer Immune Panel. Values shown are the $\log_2$ ratios of $cSiO_2$ treatment with respect to vehicle (VEH) controls plotted against the $-\log_{10}$ Benjamini-Hochberg false discovery rate *q* value. A significant difference in gene expression was inferred for genes with BH *q* value <0.05 and $\log_2$ ratio >1 or <-1. Horizontal lines indicate *q* value cutoffs of 0.1, 0.05 and 0.01, and the vertical shaded region indicates expression values that do not meet the fold change cutoff for significance. (A) Acute.1x, lung tissues from mice 1 day post single cSiO2 instillation versus dosing-matched VEH control. (B) Acute.4x, lung tissues from mice 1 day post four weekly cSiO2 instillations versus dosing-matched VEH control; (C-F) Lung.W1, Lung.W5, Lung.W9, and Lung.W13, lung tissues from cSiO2-treated (four weekly instillations) at 1, 5, 9, or 13 weeks post final instillation compared to time-matched VEH controls; (G) Kindey.W13, kidney tissues from cSiO2-treated (four weekly instillations) at 13 weeks post final instillation compared to tissue-matched VEH controls; (H)Spleen.W13, spleen tissues from cSiO2-treated (four weekly instillations) at 13 weeks post final instillation compared to tissue-matched VEH controls.

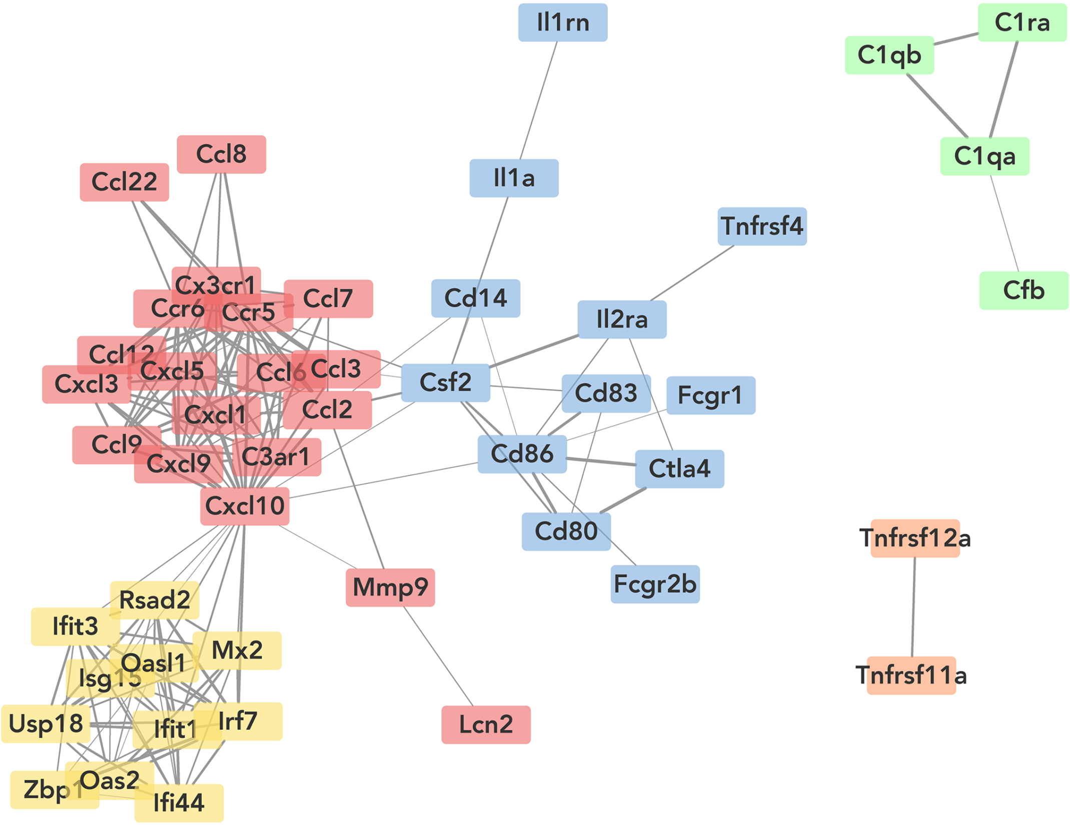

Supplementary figure 6. Network visualization of significant differentially expressed immune response genes in lung tissues of mice treated with $cSiO_2$ (four weekly doses) compared to dosing-matched vehicle controls 1 day post instillation (Acute.4x). Network interactions were modeled using the STRING database (string-db.org) with a minimum required interaction score ≥0.7, and clusters were identified using the Markov Cluster (MCL) algorithm with inflation parameter of 1.5. The network was visualized in Cytoscape, and edge widths reflect the combined interaction score (thicker edges indicate higher score). Note, a network for mice treated only once with $cSiO_2$ was not made, as only five significant genes in that treatment group were identified. Major groupings include transcripts associated with interferon signaling (yellow), cytokine and chemokines (red), and innate and adaptive immune response (blue). See Supplementary file 6 for protein-protein interactions and functional annotations obtained from the STRING database.

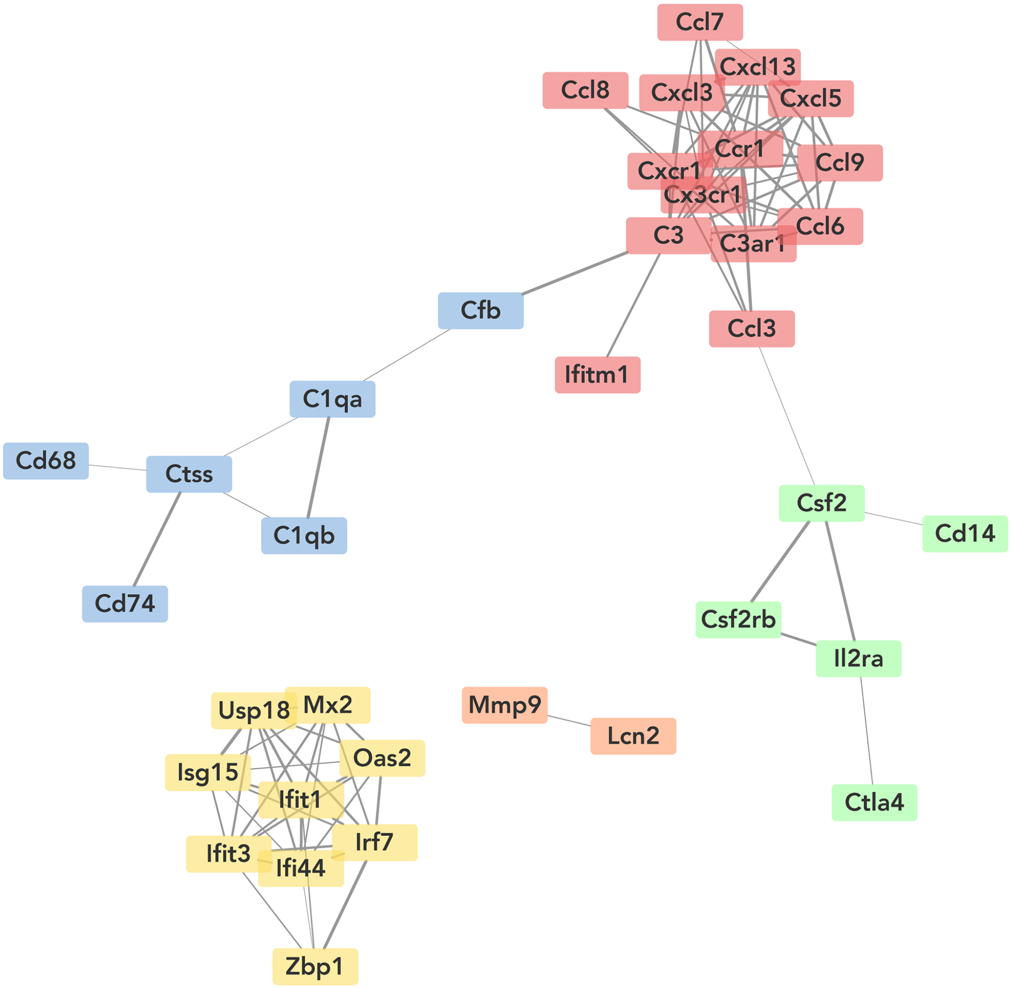

Supplementary figure 7. Network visualization of significant differentially expressed immune response genes in lung tissues of mice treated with $cSiO_2$ (four weekly doses) compared to time-matched vehicle controls 1 week post instillation (Lung.W1). Network interactions were modeled using the STRING database (string-db.org) with a minimum required interaction score ≥0.7, and clusters were identified using the Markov Cluster (MCL) algorithm with inflation parameter of 1.5. The network was visualized in Cytoscape, and edge widths reflect the combined interaction score (thicker edges indicate higher score). Major groupings include transcripts associated with interferon signaling (yellow), cytokine and chemokines (red), complement (green), and innate and adaptive immune response (blue). See Supplementary file 6 for protein-protein interactions and functional annotations obtained from the STRING database.

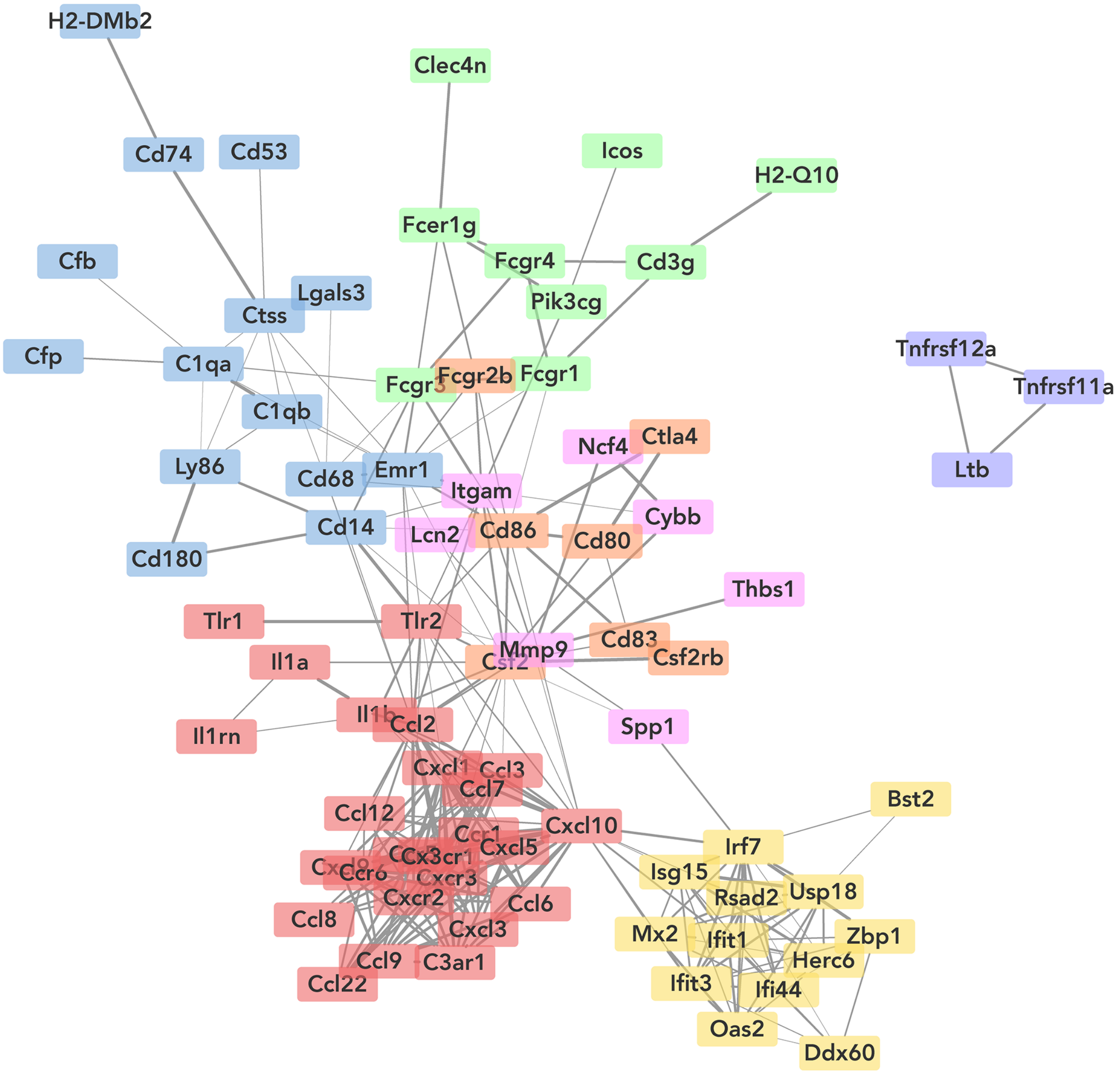

Supplementary figure 8. Network visualization of significant differentially expressed immune response genes in lung tissues of mice treated with cSiO_2 (four weekly doses) compared to time-matched vehicle controls 5 weeks post instillation (Lung.W5). Network interactions were modeled using the STRING database (string-db.org) with a minimum required interaction score ≥0.7, and clusters were identified using the Markov Cluster (MCL) algorithm with inflation parameter of 1.5. The network was visualized in Cytoscape, and edge widths reflect the combined interaction score (thicker edges indicate higher score). Major groupings include transcripts associated with interferon signaling (yellow), cytokine and chemokines (red), complement (green), and innate and adaptive immune response (blue & green). See Supplementary file 6 for protein-protein interactions and functional annotations obtained from the STRING database.

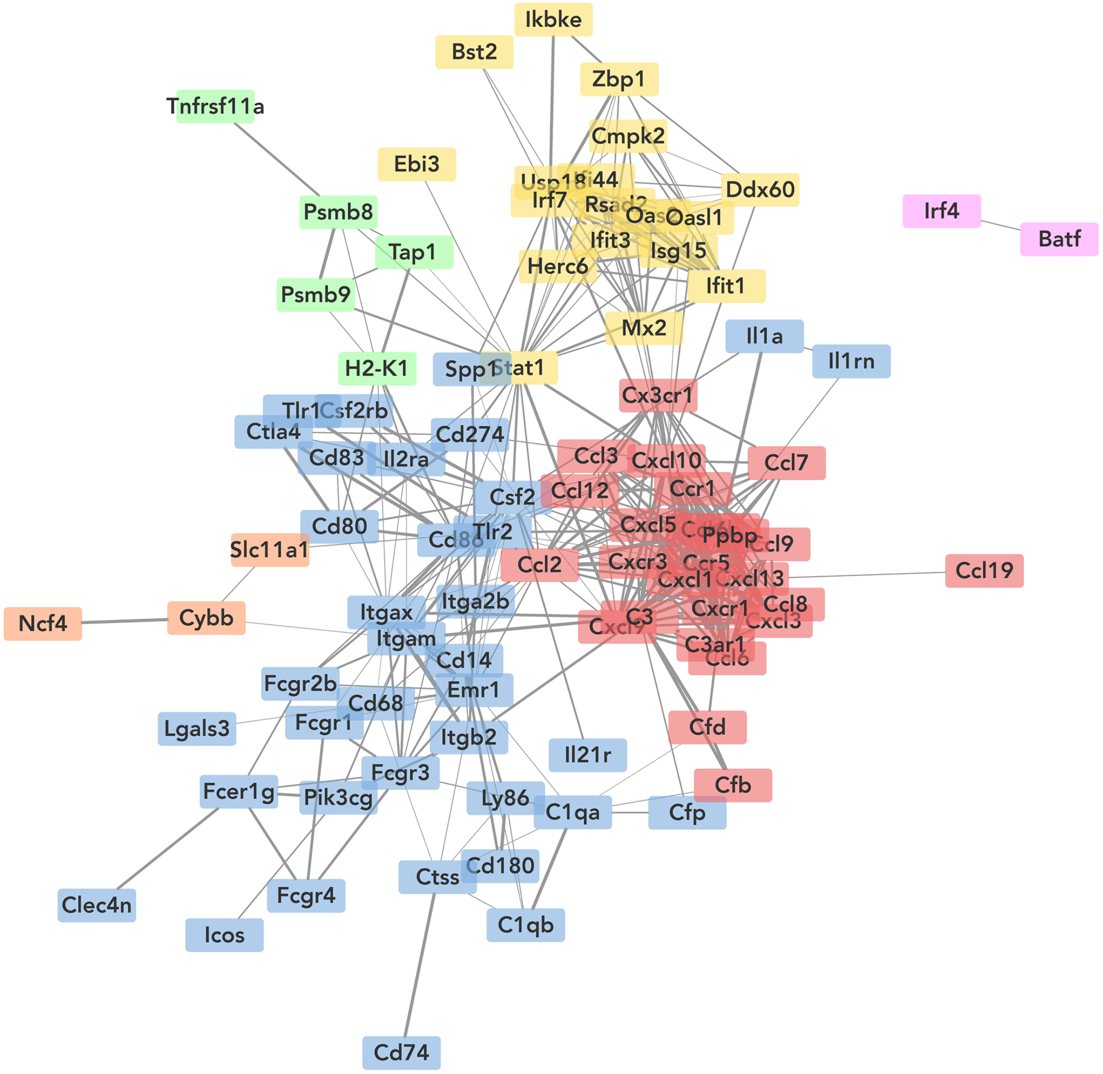

Supplementary figure 9. Network visualization of significant differentially expressed immune response genes in lung tissues of mice treated with $cSiO_2$ (four weekly doses) compared to time-matched vehicle controls 9 weeks post instillation (Lung.W9). Network interactions were modeled using the STRING database (string-db.org) with a minimum required interaction score ≥0.7, and clusters were identified using the Markov Cluster (MCL) algorithm with inflation parameter of 1.5. The network was visualized in Cytoscape, and edge widths reflect the combined interaction score (thicker edges indicate higher score). See Supplementary file 6 for protein-protein interactions and functional annotations obtained from the STRING database.

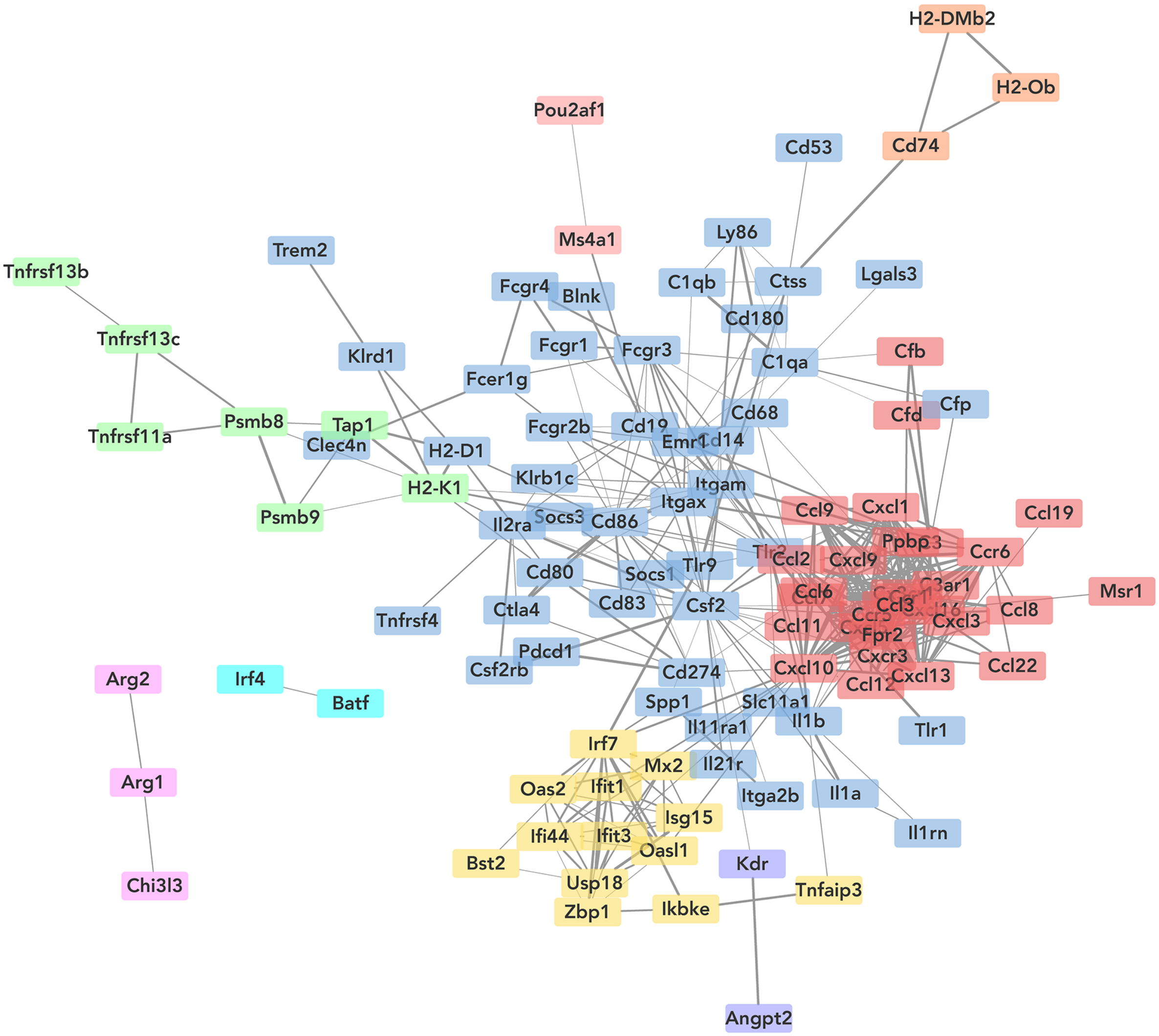

Supplementary figure 10. Network visualization of significant differentially expressed immune response genes in lung tissues of mice treated with $cSiO_2$ (four weekly doses) compared to time-matched vehicle controls 13 weeks post instillation (Lung.W13). Network interactions were modeled using the STRING database (string-db.org) with a minimum required interaction score ≥0.7, and clusters were identified using the Markov Cluster (MCL) algorithm with inflation parameter of 1.5. The network was visualized in Cytoscape, and edge widths reflect the combined interaction score (thicker edges indicate higher score). See Supplementary file 6 for protein-protein interactions and functional annotations obtained from the STRING database.

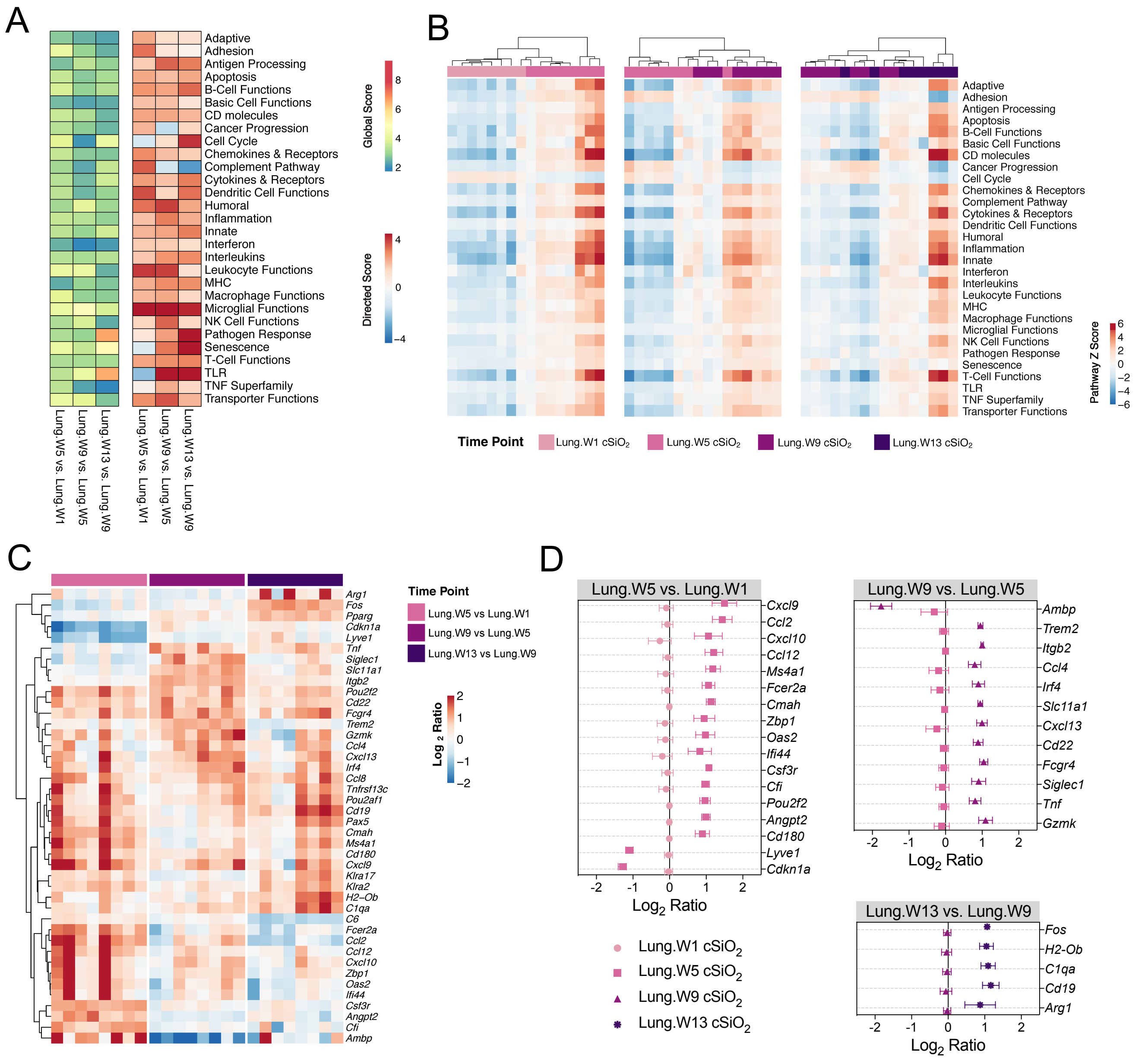

Supplementary figure 11. Comparison of transcriptional response by time point in lung tissues of $cSiO_2$-exposed mice. Mice received four repeated weekly doses of $cSiO_2$ via intranasal instillation, and gene expression was determined in lung tissues obtained at 1, 5, 9, or 13 weeks post instillation. (A) Heatmaps depicting either global or directed significance scores for immune-related pathways (see Materials and Methods for details on calculation of significance scores). (B) Heatmap depicting individual pathway Z scores of each immune pathway when comparing time points progressively: W1 vs W5, W9 vs W5, W13 vs W9. (C) Heatmap depicting gene expression for differentially expressed genes for any of the time point comparisons. Values shown are the log_2 ratio compared to the average expression for the previous time point, as indicated. (D) Selected differentially expressed genes for each time point comparison are shown as dot plots for the average log_2 ratio expression ±SEM for each comparison indicated.

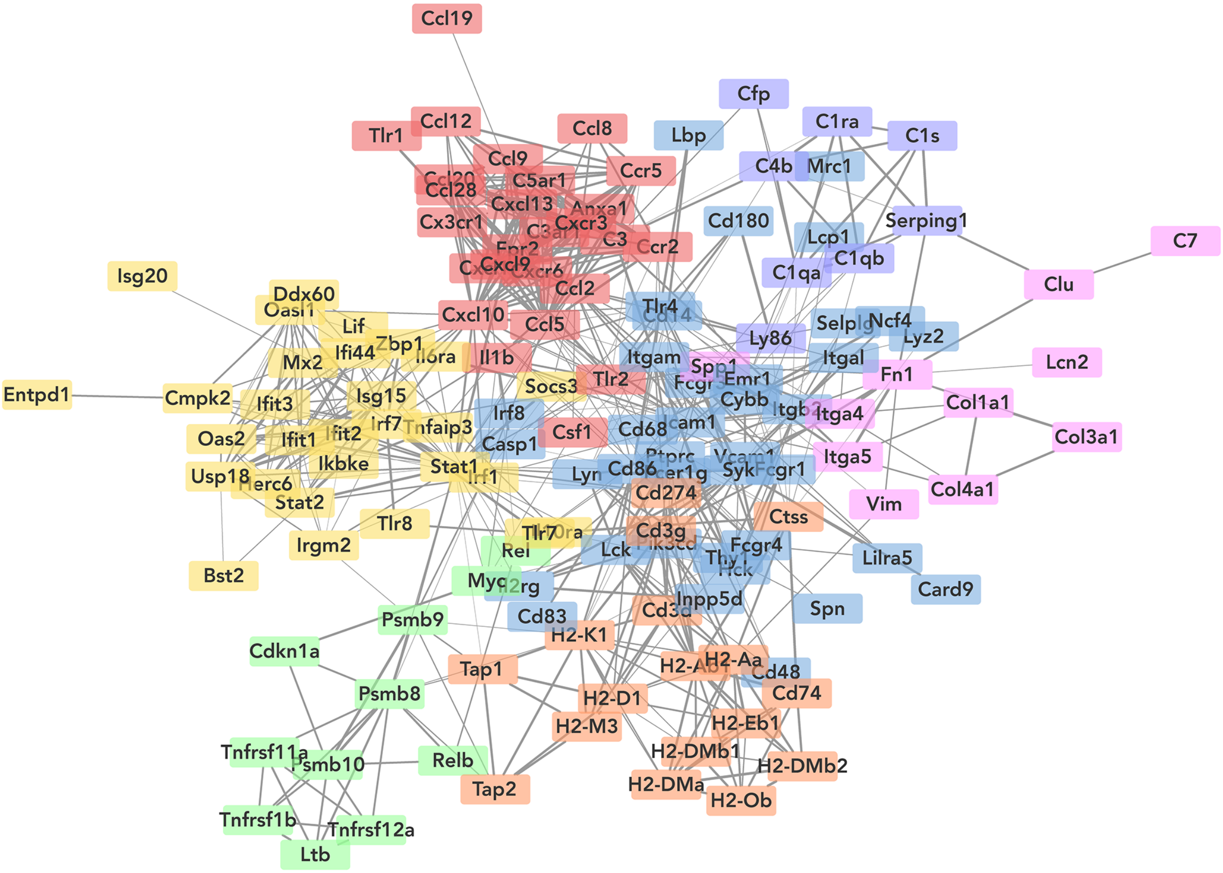

Supplementary figure 12. Network visualization of significant differentially expressed immune response genes in kidney tissues of mice treated with $cSiO_2$ (four weekly doses) compared to tissue-matched vehicle controls 13 weeks post instillation (Kidney.W13). Network interactions were modeled using the STRING database (string-db.org) with a minimum required interaction score ≥0.7, and clusters were identified using the Markov Cluster (MCL) algorithm with inflation parameter of 1.5. The network was visualized in Cytoscape, and edge widths reflect the combined interaction score (thicker edges indicate higher score). See Supplementary file 6 for protein-protein interactions and functional annotations obtained from the STRING database.

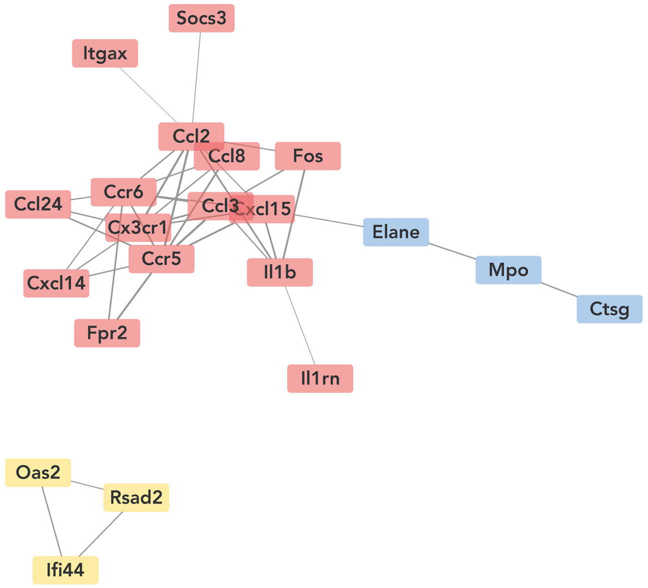

Supplementary figure 13. Network visualization significant differentially expressed immune response genes in spleen tissues of mice treated with $cSiO_2$ (four weekly doses) compared to tissue-matched vehicle controls 13 weeks post instillation (Spleen.W13). Network interactions were modeled using the STRING database (string-db.org) with a minimum required interaction score ≥0.7, and clusters were identified using the Markov Cluster (MCL) algorithm with inflation parameter of 1.5. The network was visualized in Cytoscape, and edge widths reflect the combined interaction score (thicker edges indicate higher score). See Supplementary file 6 for protein-protein interactions and functional annotations obtained from the STRING database.

Supplementary Table 1. Significant differentially expressed genes in lung tissues of mice following single $cSiO_2$ dose (experiment 1)

Gene symbol	Log_2 ratio $cSiO_2$/VEH	SEM	95% CI	FDR *q* value	Associated pathways
Ccl24	3.28	0.767	1.78, 4.79	0.0459	Adaptive, Chemokines & Receptors, Cytokines & Receptors, Inflammation
Arg1	2.82	0.674	1.50, 4.14	0.0465	Basic Cell Functions
Chil3	2.65	0.641	1.39, 3.91	0.0481	Basic Cell Functions
Cxcl5	2.54	0.466	1.62, 3.45	0.0288	Chemokines & Receptors, Cytokines & Receptors, Inflammation, Leukocyte Functions
Lcn2	1.76	0.391	0.995, 2.53	0.0459	Apoptosis, Innate, Transporter Functions

Note: Values shown are the calculated mean log_2 ratios, SEM and 95% confidence intervals of transcript counts for lung tissues obtained from $cSiO_2$-treated mice with respect to dosing-matched (single dose) vehicle treated mice 1 day post instillation. Statistical analysis was performed using nSolver software (nanoString) with the Benjamini-Hochberg method for control of false discoveries (α=0.05); FDR-corrected *q* values are shown. Pathway associations for each significant gene are also shown.

Supplementary Table 2. Significant differentially expressed genes in lung tissues of mice following four $cSiO_2$ doses (experiment 2).

Gene symbol	Log_2 ratio $cSiO_2$/VEH	SEM	95% CI	FDR *q* value	Associated pathways
Cxcl5	3.74	0.462	2.83, 4.64	1.22E-05	Chemokines & Receptors, Cytokines & Receptors, Inflammation, Leukocyte Functions
Cxcl1	2.70	0.314	2.08, 3.31	7.23E-06	Adaptive, Chemokines & Receptors, Cytokines & Receptors, Inflammation, Transporter Functions
Ctla4	2.67	0.236	2.20, 3.13	1.24E-06	B-Cell Functions, CD molecules, T-Cell Functions
Cxcl10	2.66	0.430	1.81, 3.50	1.20E-04	Adaptive, Chemokines & Receptors, Cytokines & Receptors, Inflammation, Innate, Leukocyte Functions
Oasl1	2.42	0.328	1.78, 3.06	2.54E-05	Basic Cell Functions
Ccl2	2.37	0.270	1.85, 2.90	5.77E-06	Chemokines & Receptors, Cytokines & Receptors, Humoral, Inflammation, Innate, Interleukins, Macrophage Functions, NK Cell Functions, Pathogen Response, T-Cell Functions
Lcn2	2.33	0.283	1.77, 2.88	1.04E-05	Apoptosis, Innate, Transporter Functions
Irf7	2.30	0.303	1.70, 2.89	2.01E-05	Adaptive, Innate, Interferon, Senescence
Mx2	2.28	0.291	1.71, 2.85	1.50E-05	Innate
Ifi44	2.18	0.282	1.63, 2.73	1.68E-05	Interferon
Oas2	2.14	0.272	1.61, 2.68	1.43E-05	Basic Cell Functions, Interferon
Isg15	2.03	0.271	1.50, 2.56	2.18E-05	Basic Cell Functions, Innate, Interferon
Ccl3	2.03	0.293	1.46, 2.60	4.48E-05	Adaptive, Chemokines & Receptors, Cytokines & Receptors, Humoral, Inflammation, NK Cell Functions, T-Cell Functions, Transporter Functions
Ccl7	1.90	0.236	1.44, 2.37	1.23E-05	Adaptive, Cancer Progression, Chemokines & Receptors, Cytokines & Receptors, Humoral, Inflammation, NK Cell Functions, T-Cell Functions
Csf2	1.81	0.158	1.50, 2.12	1.17E-06	Adaptive, Cytokines & Receptors, Innate, Interleukins, Macrophage Functions, T-Cell Functions, Transporter Functions
Clec5a	1.78	0.138	1.51, 2.05	4.69E-07	Apoptosis, Cytokines & Receptors, Innate
Trem2	1.77	0.166	1.44, 2.09	1.75E-06	Basic Cell Functions, Humoral
Marco	1.76	0.184	1.40, 2.13	2.91E-06	Innate, Transporter Functions
Ifit1	1.75	0.266	1.22, 2.27	7.17E-05	Interferon, Transporter Functions
Il2ra	1.72	0.160	1.40, 2.03	1.75E-06	Adaptive, B-Cell Functions, CD molecules, Inflammation, T-Cell Functions
Mmp9	1.71	0.249	1.22, 2.20	4.83E-05	Adhesion, Apoptosis, Cancer Progression

C3ar1	1.67	0.178	1.32, 2.02	3.12E-06	Adaptive, Cancer Progression, Complement Pathway, Inflammation, Macrophage Functions
Zbp1	1.65	0.241	1.17, 2.12	5.03E-05	Innate
Il1rn	1.62	0.279	1.08, 2.17	1.87E-04	Cytokines & Receptors, Inflammation, Interleukins
Cfb	1.60	0.168	1.28, 1.93	2.91E-06	Complement Pathway, Innate
C1qa	1.54	0.102	1.34, 1.74	2.82E-07	Complement Pathway, Innate
Ccl12	1.52	0.191	1.15, 1.90	1.37E-05	Adaptive, Chemokines & Receptors, Cytokines & Receptors, Humoral, Inflammation
Cxcl9	1.51	0.523	0.484, 2.54	2.55E-02	Adaptive, Chemokines & Receptors, Cytokines & Receptors, Inflammation, Innate
Msr1	1.44	0.161	1.13, 1.76	4.66E-06	CD molecules, Macrophage Functions, Transporter Functions
Cxcl3	1.42	0.484	0.468, 2.37	2.37E-02	Adaptive, Chemokines & Receptors, Cytokines & Receptors, Inflammation
C1qb	1.42	0.117	1.19, 1.65	8.30E-07	Complement Pathway, Innate
Usp18	1.41	0.223	0.974, 1.85	9.82E-05	Basic Cell Functions
Rsad2	1.38	0.298	0.798, 1.97	1.23E-03	Basic Cell Functions
Siglec1	1.37	0.186	1.00, 1.73	2.60E-05	Adhesion, CD molecules, Transporter Functions
Ccl9	1.36	0.134	1.10, 1.63	2.38E-06	Chemokines & Receptors, Cytokines & Receptors
Clec4n	1.35	0.146	1.07, 1.64	3.38E-06	Cytokines & Receptors, Innate
Ifit3	1.30	0.219	0.87, 1.73	1.66E-04	Interferon
Ccl8	1.27	0.482	0.325, 2.22	3.87E-02	Adaptive, Cancer Progression, Chemokines & Receptors, Cytokines & Receptors, Inflammation
Fcgr1	1.26	0.120	1.03, 1.50	1.85E-06	Antigen Processing, CD molecules, Innate, MHC, Transporter Functions
Ccr5	1.19	0.155	0.886, 1.49	1.76E-05	Adaptive, CD molecules, Chemokines & Receptors, Cytokines & Receptors, Dendritic Cell Functions, Macrophage Functions, T-Cell Functions, Transporter Functions
Icos	1.17	0.114	0.948, 1.39	2.14E-06	CD molecules, T-Cell Functions
Tnfrsf4	1.15	0.115	0.924, 1.37	2.69E-06	B-Cell Functions, CD molecules, Cytokines & Receptors, Inflammation, T-Cell Functions, TNF Superfamily
Tnfrsf11a	1.13	0.102	0.930, 1.33	1.42E-06	Adaptive, CD molecules, Cytokines & Receptors, Interleukins, TNF Superfamily
Cd83	1.12	0.115	0.894, 1.34	2.77E-06	B-Cell Functions, CD molecules, Dendritic Cell Functions, Humoral, Interleukins, T-Cell Functions
Slc11a1	1.12	0.183	0.766, 1.48	1.24E-04	Adaptive, Antigen Processing, Transporter Functions

Cd80	1.11	0.119	0.875, 1.34	3.37E-06	Adaptive, CD molecules, T-Cell Functions
Cx3cr1	1.11	0.084	0.949, 1.28	4.51E-07	Chemokines & Receptors, Cytokines & Receptors, Macrophage Functions, Microglial Functions
Cd14	1.09	0.159	0.779, 1.4	4.83E-05	CD molecules, Cytokines & Receptors, Inflammation, Innate, Pathogen Response, Transporter Functions
Fcgr2b	1.08	0.132	0.822, 1.34	1.10E-05	Antigen Processing, B-Cell Functions, CD molecules, Inflammation, Interleukins, MHC, Transporter Functions
Ccr6	1.07	0.186	0.710, 1.44	2.03E-04	Adaptive, CD molecules, Chemokines & Receptors, Humoral, Innate, T-Cell Functions
Il1a	1.06	0.201	0.668, 1.46	4.45E-04	Adaptive, Cytokines & Receptors, Inflammation, Innate, Interleukins
Cd86	1.05	0.128	0.796, 1.30	1.11E-05	Adaptive, B-Cell Functions, CD molecules, Dendritic Cell Functions, T-Cell Functions, TLR
Ccl22	1.04	0.183	0.685, 1.40	2.28E-04	Chemokines & Receptors, Cytokines & Receptors, Humoral, Inflammation
Ccl6	1.01	0.104	0.809, 1.21	2.77E-06	Chemokines & Receptors, Cytokines & Receptors
C1ra	1.00	0.084	0.839, 1.17	8.30E-07	Complement Pathway, Innate

Note: Values shown are the calculated mean $\log_2$ ratios, SEM and 95% confidence intervals of transcript counts for lung tissues of $cSiO_2$-treated mice with respect to dosing-matched (four weekly doses) vehicle-treated mice 1 day post instillation. Statistical analysis was performed using nSolver software (nanoString) with the Benjamini-Hochberg method for control of false discoveries (α=0.05); FDR-corrected *q* values are shown. Pathway associations for each significant gene are also shown.

Supplementary Table 3. Significant differentially expressed genes in lung tissues of mice at 1 week post $cSiO_2$ instillation (experiment 3).

Gene symbol	Log$_2$ ratio $cSiO_2$/VEH	SEM	95% CI	FDR *q* value	Associated pathways
Cxcl5	3.63	0.472	2.71, 4.56	4.43E-05	Chemokines & Receptors, Cytokines & Receptors, Inflammation, Leukocyte Functions
Cxcl3	3.21	0.413	2.40, 4.02	4.09E-05	Adaptive, Chemokines & Receptors, Cytokines & Receptors, Inflammation
Lcn2	2.77	0.204	2.37, 3.17	4.13E-07	Apoptosis, Innate, Transporter Functions
Ctla4	2.66	0.188	2.29, 3.03	3.52E-07	B-Cell Functions, CD molecules, T-Cell Functions
Chil3	2.58	0.498	1.61, 3.56	7.11E-04	Basic Cell Functions
Slc11a1	2.22	0.354	1.53, 2.92	1.85E-04	Adaptive, Antigen Processing, Transporter Functions
Irf7	2.22	0.372	1.49, 2.95	2.68E-04	Adaptive, Innate, Interferon, Senescence
Mmp9	2.22	0.401	1.43, 3.00	4.58E-04	Adhesion, Apoptosis, Cancer Progression
Marco	2.03	0.261	1.52, 2.54	4.09E-05	Innate, Transporter Functions
Cfb	1.83	0.120	1.59, 2.06	2.90E-07	Complement Pathway, Innate
Il1rn	1.82	0.145	1.53, 2.10	8.66E-07	Cytokines & Receptors, Inflammation, Interleukins
Ccl8	1.78	0.335	1.13, 2.44	6.13E-04	Adaptive, Cancer Progression, Chemokines & Receptors, Cytokines & Receptors, Inflammation
Ccl3	1.75	0.284	1.20, 2.31	2.03E-04	Adaptive, Chemokines & Receptors, Cytokines & Receptors, Humoral, Inflammation, NK Cell Functions, T-Cell Functions, Transporter Functions
Trem2	1.68	0.159	1.36, 1.99	2.71E-06	Basic Cell Functions, Humoral
Clec5a	1.65	0.148	1.36, 1.94	1.77E-06	Apoptosis, Cytokines & Receptors, Innate
Mx2	1.64	0.302	1.04, 2.23	5.43E-04	Innate
Isg15	1.60	0.308	1.00, 2.21	7.00E-04	Basic Cell Functions, Innate, Interferon
C3ar1	1.58	0.136	1.31, 1.84	1.52E-06	Adaptive, Cancer Progression, Complement Pathway, Inflammation, Macrophage Functions
Oas2	1.56	0.237	1.09, 2.02	1.34E-04	Basic Cell Functions, Interferon
Siglec1	1.50	0.247	1.02, 1.98	2.28E-04	Adhesion, CD molecules, Transporter Functions
Cxcr1	1.49	0.189	1.12, 1.86	4.00E-05	Adaptive, CD molecules, Chemokines & Receptors, Cytokines & Receptors, Dendritic Cell Functions, Inflammation, Interleukins

Ifi44	1.48	0.280	0.93, 2.03	6.52E-04	Interferon
Msr1	1.45	0.221	1.02, 1.88	1.34E-04	CD molecules, Macrophage Functions, Transporter Functions
Ccl9	1.42	0.159	1.11, 1.73	1.29E-05	Chemokines & Receptors, Cytokines & Receptors
C1qa	1.39	0.125	1.14, 1.63	1.77E-06	Complement Pathway, Innate
Csf2	1.39	0.137	1.12, 1.66	3.77E-06	Adaptive, Cytokines & Receptors, Innate, Interleukins, Macrophage Functions, T-Cell Functions, Transporter Functions
Ifit1	1.37	0.262	0.856, 1.88	6.92E-04	Interferon, Transporter Functions
C1qb	1.32	0.119	1.08, 1.55	1.77E-06	Complement Pathway, Innate
Ccl6	1.30	0.127	1.05, 1.55	3.54E-06	Chemokines & Receptors, Cytokines & Receptors
Ccl7	1.28	0.284	0.728, 1.84	2.04E-03	Adaptive, Cancer Progression, Chemokines & Receptors, Cytokines & Receptors, Humoral, Inflammation, NK Cell Functions, T-Cell Functions
Cxcl13	1.28	0.341	0.614, 1.95	7.13E-03	Adaptive, B-Cell Functions, Chemokines & Receptors, Cytokines & Receptors, Humoral, Inflammation, T-Cell Functions
Slc7a11	1.26	0.248	0.771, 1.74	8.37E-04	Transporter Functions
Clec4n	1.23	0.145	0.942, 1.51	2.02E-05	Cytokines & Receptors, Innate
Zbp1	1.22	0.250	0.732, 1.71	1.11E-03	Innate
Ctss	1.20	0.133	0.935, 1.46	1.27E-05	Innate
Cx3cr1	1.18	0.167	0.856, 1.51	8.30E-05	Chemokines & Receptors, Cytokines & Receptors, Macrophage Functions, Microglial Functions
Cd14	1.14	0.130	0.887, 1.40	1.52E-05	CD molecules, Cytokines & Receptors, Inflammation, Innate, Pathogen Response, Transporter Functions
Cd74	1.14	0.169	0.806, 1.47	1.11E-04	Antigen Processing, B-Cell Functions, CD molecules, Innate, MHC, T-Cell Functions
Ccr1	1.14	0.204	0.742, 1.54	4.26E-04	Adaptive, CD molecules, Chemokines & Receptors, Cytokines & Receptors, Dendritic Cell Functions, Inflammation, Innate, Leukocyte Functions, Transporter Functions
C3	1.13	0.0988	0.940, 1.33	1.55E-06	Cancer Progression, Complement Pathway, Inflammation, Innate, Transporter Functions
Fcgr1	1.10	0.178	0.750, 1.45	2.03E-04	Antigen Processing, CD molecules, Innate, MHC, Transporter Functions
Ifit3	1.10	0.248	0.615, 1.59	2.35E-03	Interferon
Il2ra	1.09	0.147	0.800, 1.38	6.40E-05	Adaptive, B-Cell Functions, CD molecules, Inflammation, T-Cell Functions
Csf2rb	1.09	0.155	0.791, 1.40	8.43E-05	CD molecules, Cancer Progression, Cytokines & Receptors

Cd68	1.08	0.127	0.829, 1.33	2.02E-05	CD molecules
Ifitm1	1.06	0.0871	0.892, 1.23	9.87E-07	CD molecules, Innate, Interferon
Slamf7	1.06	0.191	0.682, 1.43	4.57E-04	CD molecules, Innate, NK Cell Functions
Icos	1.05	0.0975	0.862, 1.24	2.11E-06	CD molecules, T-Cell Functions
Cd33	1.04	0.115	0.818, 1.27	1.22E-05	Adhesion, CD molecules
Usp18	1.03	0.238	0.564, 1.50	2.82E-03	Basic Cell Functions

Note: Values shown are the calculated mean $\log_2$ ratios, SEM and 95% confidence intervals of transcript counts for lung tissues of $cSiO_2$-treated mice with respect to time-matched vehicle-treated mice 1 week post instillation. Statistical analysis was performed using nSolver software (nanoString) with the Benjamini-Hochberg method for control of false discoveries (α=0.05); FDR-corrected q values are shown. Pathway associations for each significant gene are also shown.

Supplementary Table 4. Significant differentially expressed genes in lung tissues of mice at 5 weeks post $cSiO_2$ instillation (experiment 3).

Gene symbol	Log$_2$ ratio $cSiO_2$/VEH	SEM	95% CI	FDR *q* value	Associated pathways
Cxcl5	4.96	0.270	4.43, 5.49	7.12E-08	Chemokines & Receptors, Cytokines & Receptors, Inflammation, Leukocyte Functions
Cxcl3	4.95	0.515	3.94, 5.95	3.96E-06	Adaptive, Chemokines & Receptors, Cytokines & Receptors, Inflammation
Ccl3	3.75	0.228	3.30, 4.20	7.35E-08	Adaptive, Chemokines & Receptors, Cytokines & Receptors, Humoral, Inflammation, NK Cell Functions, T-Cell Functions, Transporter Functions
Lcn2	3.50	0.210	3.09, 3.91	7.35E-08	Apoptosis, Innate, Transporter Functions
Cxcl10	3.32	0.601	2.14, 4.50	3.93E-04	Adaptive, Chemokines & Receptors, Cytokines & Receptors, Inflammation, Innate, Leukocyte Functions
Ccl2	3.03	0.338	2.36, 3.69	6.73E-06	Chemokines & Receptors, Cytokines & Receptors, Humoral, Inflammation, Innate, Interleukins, Macrophage Functions, NK Cell Functions, Pathogen Response, T-Cell Functions
Ccl8	2.93	0.383	2.18, 3.68	2.81E-05	Adaptive, Cancer Progression, Chemokines & Receptors, Cytokines & Receptors, Inflammation
Il1rn	2.89	0.266	2.36, 3.41	1.56E-06	Cytokines & Receptors, Inflammation, Interleukins
Cxcl1	2.83	0.247	2.35, 3.32	1.24E-06	Adaptive, Chemokines & Receptors, Cytokines & Receptors, Inflammation, Transporter Functions
Cxcl9	2.72	0.607	1.53, 3.91	1.80E-03	Adaptive, Chemokines & Receptors, Cytokines & Receptors, Inflammation, Innate
Clec5a	2.62	0.199	2.23, 3.01	4.58E-07	Apoptosis, Cytokines & Receptors, Innate
Irf7	2.60	0.413	1.79, 3.41	1.50E-04	Adaptive, Innate, Interferon, Senescence
Ccl7	2.49	0.402	1.70, 3.28	1.66E-04	Adaptive, Cancer Progression, Chemokines & Receptors, Cytokines & Receptors, Humoral, Inflammation, NK Cell Functions, T-Cell Functions
Msr1	2.45	0.248	1.96, 2.94	3.05E-06	CD molecules, Macrophage Functions, Transporter Functions
C3ar1	2.43	0.260	1.92, 2.94	4.59E-06	Adaptive, Cancer Progression, Complement Pathway, Inflammation, Macrophage Functions
Ifi44	2.35	0.336	1.69, 3.01	5.91E-05	Interferon
Ctla4	2.33	0.217	1.91, 2.76	1.58E-06	B-Cell Functions, CD molecules, T-Cell Functions
Marco	2.32	0.232	1.86, 2.77	2.98E-06	Innate, Transporter Functions
Oas2	2.26	0.291	1.69, 2.83	2.38E-05	Basic Cell Functions, Interferon
Mmp9	2.20	0.160	1.89, 2.52	3.28E-07	Adhesion, Apoptosis, Cancer Progression

Cfb	2.19	0.170	1.85, 2.52	4.85E-07	Complement Pathway, Innate
Isg15	2.13	0.365	1.41, 2.84	2.61E-04	Basic Cell Functions, Innate, Interferon
Clec4n	2.12	0.153	1.82, 2.42	3.27E-07	Cytokines & Receptors, Innate
Trem2	2.12	0.189	1.75, 2.49	1.30E-06	Basic Cell Functions, Humoral
Ccl12	2.11	0.347	1.43, 2.78	1.92E-04	Adaptive, Chemokines & Receptors, Cytokines & Receptors, Humoral, Inflammation
Zbp1	2.07	0.315	1.45, 2.69	1.05E-04	Innate
Csf2	2.00	0.176	1.65, 2.34	1.24E-06	Adaptive, Cytokines & Receptors, Innate, Interleukins, Macrophage Functions, T-Cell Functions, Transporter Functions
Ccl9	1.98	0.140	1.71, 2.26	3.16E-07	Chemokines & Receptors, Cytokines & Receptors
Chil3	1.95	0.376	1.21, 2.69	6.48E-04	Basic Cell Functions
Ccr6	1.92	0.308	1.32, 2.52	1.57E-04	Adaptive, CD molecules, Chemokines & Receptors, Humoral, Innate, T-Cell Functions
Ifit1	1.85	0.303	1.25, 2.44	1.88E-04	Interferon, Transporter Functions
Mx2	1.83	0.377	1.09, 2.57	1.02E-03	Innate
Spp1	1.81	0.195	1.43, 2.19	5.09E-06	Adhesion, Apoptosis, Cytokines & Receptors, T-Cell Functions
Fcgr1	1.76	0.270	1.23, 2.29	1.13E-04	Antigen Processing, CD molecules, Innate, MHC, Transporter Functions
Il1a	1.75	0.156	1.45, 2.06	1.30E-06	Adaptive, Cytokines & Receptors, Inflammation, Innate, Interleukins
C1qa	1.75	0.156	1.44, 2.06	1.30E-06	Complement Pathway, Innate
Usp18	1.74	0.286	1.17, 2.30	1.94E-04	Basic Cell Functions
Ccl6	1.70	0.197	1.32, 2.09	9.26E-06	Chemokines & Receptors, Cytokines & Receptors
Clec7a	1.70	0.241	1.23, 2.17	5.69E-05	Inflammation, Innate, Leukocyte Functions, Transporter Functions
Blnk	1.69	0.264	1.17, 2.20	1.31E-04	B-Cell Functions, Humoral
S100a8	1.64	0.181	1.29, 2.00	5.98E-06	Adaptive, Basic Cell Functions, Inflammation
C1qb	1.63	0.157	1.32, 1.93	2.38E-06	Complement Pathway, Innate
Ccr1	1.61	0.162	1.30, 1.93	2.98E-06	Adaptive, CD molecules, Chemokines & Receptors, Cytokines & Receptors, Dendritic Cell Functions, Inflammation, Innate, Leukocyte Functions, Transporter Functions
Ctss	1.57	0.197	1.18, 1.95	1.95E-05	Innate

Cd14	1.52	0.139	1.25, 1.79	1.56E-06	CD molecules, Cytokines & Receptors, Inflammation, Innate, Pathogen Response, Transporter Functions
Fcgr2b	1.50	0.131	1.25, 1.76	1.24E-06	Antigen Processing, B-Cell Functions, CD molecules, Inflammation, Interleukins, MHC, Transporter Functions
Rsad2	1.50	0.346	0.820, 2.17	2.34E-03	Basic Cell Functions
Ccr5	1.48	0.272	0.952, 2.02	4.31E-04	Adaptive, CD molecules, Chemokines & Receptors, Cytokines & Receptors, Dendritic Cell Functions, Macrophage Functions, T-Cell Functions, Transporter Functions
Ccl22	1.47	0.258	0.962, 1.98	3.16E-04	Chemokines & Receptors, Cytokines & Receptors, Humoral, Inflammation
Cd83	1.44	0.129	1.18, 1.69	1.32E-06	B-Cell Functions, CD molecules, Dendritic Cell Functions, Humoral, Interleukins, T-Cell Functions
Icos	1.41	0.180	1.06, 1.77	2.23E-05	CD molecules, T-Cell Functions
Slamf7	1.41	0.252	0.919, 1.91	3.51E-04	CD molecules, Innate, NK Cell Functions
Tlr2	1.37	0.118	1.13, 1.60	1.24E-06	CD molecules, Inflammation, Innate, Leukocyte Functions, TLR
Arg2	1.37	0.168	1.04, 1.70	1.52E-05	Basic Cell Functions
H2-DMb2	1.37	0.222	0.931, 1.80	1.75E-04	Antigen Processing, MHC
Il1b	1.34	0.133	1.07, 1.60	2.98E-06	Adaptive, Chemokines & Receptors, Humoral, Inflammation, Innate, Interleukins, Macrophage Functions, Pathogen Response, T-Cell Functions, Transporter Functions
Fcgr3	1.34	0.193	0.964, 1.72	6.21E-05	Antigen Processing, CD molecules, MHC, Transporter Functions
Ly86	1.32	0.162	1.01, 1.64	1.52E-05	Humoral, Inflammation, Innate, Pathogen Response
Ifit3	1.32	0.262	0.804, 1.83	8.00E-04	Interferon
Cd80	1.28	0.175	0.935, 1.62	4.30E-05	Adaptive, CD molecules, T-Cell Functions
Emr1	1.28	0.191	0.907, 1.65	8.61E-05	Basic Cell Functions
Cd180	1.28	0.226	0.833, 1.72	3.33E-04	CD molecules, Inflammation, Innate
Cd68	1.26	0.096	1.07, 1.44	4.59E-07	CD molecules
Cd86	1.24	0.137	0.974, 1.51	6.08E-06	Adaptive, B-Cell Functions, CD molecules, Dendritic Cell Functions, T-Cell Functions, TLR
Ly9	1.24	0.205	0.833, 1.64	2.04E-04	Adhesion, CD molecules
Ncf4	1.21	0.106	1.00, 1.42	1.24E-06	Basic Cell Functions, Innate
Cd53	1.20	0.089	1.02, 1.37	3.97E-07	CD molecules

Pik3cg	1.20	0.138	0.927, 1.47	8.98E-06	Apoptosis, Inflammation, Innate
Cx3cr1	1.20	0.200	0.806, 1.59	2.08E-04	Chemokines & Receptors, Cytokines & Receptors, Macrophage Functions, Microglial Functions
Ddx60	1.20	0.248	0.715, 1.69	1.05E-03	Basic Cell Functions
Cd200r1	1.19	0.124	0.948, 1.44	3.96E-06	B-Cell Functions
Cfp	1.19	0.175	0.850, 1.54	7.66E-05	Cancer Progression, Complement Pathway, Innate
Clec4a2	1.19	0.189	0.821, 1.56	1.50E-04	Innate
Slamf6	1.19	0.230	0.734, 1.64	6.81E-04	CD molecules
Fcer1g	1.18	0.108	0.964, 1.39	1.56E-06	Antigen Processing, Interleukins, MHC, Transporter Functions
Ifitm1	1.18	0.116	0.955, 1.41	2.64E-06	CD molecules, Innate, Interferon
Irf4	1.18	0.321	0.548, 1.81	6.63E-03	Interleukins, T-Cell Functions, TLR
Cxcr2	1.17	0.155	0.863, 1.47	3.19E-05	Adaptive, CD molecules, Chemokines & Receptors, Cytokines & Receptors, Inflammation, Innate, Interleukins
Ebi3	1.16	0.123	0.915, 1.40	4.56E-06	Cytokines & Receptors, Humoral, Interleukins
Cybb	1.15	0.127	0.896, 1.39	6.37E-06	Innate, Transporter Functions
Ms4a1	1.15	0.236	0.691, 1.62	9.91E-04	B-Cell Functions, CD molecules, Humoral
Fcgr4	1.14	0.229	0.694, 1.59	8.54E-04	CD molecules, T-Cell Functions
Tnfrsf11a	1.12	0.152	0.823, 1.42	3.95E-05	Adaptive, CD molecules, Cytokines & Receptors, Interleukins, TNF Superfamily
H2-Q10	1.11	0.284	0.551, 1.67	4.58E-03	Adaptive, Antigen Processing, B-Cell Functions, MHC
Ltb	1.09	0.179	0.737, 1.44	1.94E-04	Cytokines & Receptors, Interleukins, TNF Superfamily
Cd74	1.07	0.105	0.861, 1.27	2.64E-06	Antigen Processing, B-Cell Functions, CD molecules, Innate, MHC, T-Cell Functions
Tlr1	1.07	0.209	0.661, 1.48	7.04E-04	CD molecules, Inflammation, Innate, Interleukins, Macrophage Functions, TLR
Herc6	1.06	0.206	0.659, 1.47	6.73E-04	Basic Cell Functions
Itgam	1.04	0.144	0.761, 1.32	4.50E-05	Adaptive, CD molecules, Innate, Leukocyte Functions, T-Cell Functions, Transporter Functions
Bst2	1.04	0.204	0.644, 1.44	7.10E-04	CD molecules, Humoral, Innate
Cxcr3	1.04	0.317	0.417, 1.66	1.29E-02	Adaptive, CD molecules, Chemokines & Receptors, Cytokines & Receptors, Innate, T-Cell Functions

Lgals3	1.02	0.108	0.812, 1.23	4.25E-06	Innate, T-Cell Functions
Csf2rb	1.01	0.174	0.669, 1.35	2.66E-04	CD molecules, Cancer Progression, Cytokines & Receptors
Cd84	1.00	0.082	0.841, 1.16	8.07E-07	Adhesion, CD molecules
Cd5	1.00	0.157	0.695, 1.31	1.33E-04	Apoptosis, CD molecules, T-Cell Functions
Cd3g	1.00	0.164	0.680, 1.32	1.84E-04	Apoptosis, CD molecules, T-Cell Functions, Transporter Functions
Tnfrsf12a	-1.12	0.113	-1.34, -0.897	3.05E-06	Adhesion, Apoptosis, CD molecules, TNF Superfamily
Cdkn1a	-1.51	0.152	-1.80, -1.21	3.05E-06	B-Cell Functions, Cancer Progression, Cell Cycle, Senescence
Thbs1	-1.56	0.172	-1.90, -1.22	5.98E-06	Adaptive, Cell Cycle, Inflammation, Macrophage Functions

Note: Values shown are the calculated mean $\log_2$ ratios, SEM and 95% confidence intervals of transcript counts for lung tissues of $cSiO_2$-treated mice with respect to time-matched vehicle-treated mice 5 weeks post instillation. Statistical analysis was performed using nSolver software (nanoString) with the Benjamini-Hochberg method for control of false discoveries (α=0.05); FDR-corrected q values are shown. Pathway associations for each significant gene are also shown.

Supplementary Table 5. Significant differentially expressed genes in lung tissues of mice at 9 weeks post $cSiO_2$ instillation (experiment 3).

Gene symbol	Log_2 ratio $cSiO_2$/VEH	SEM	95% CI	FDR *q* value	Associated pathways
Cxcl5	5.22	0.284	4.66, 5.77	3.34E-09	Chemokines & Receptors, Cytokines & Receptors, Inflammation, Leukocyte Functions
Ccl2	4.20	0.283	3.64, 4.75	1.64E-08	Chemokines & Receptors, Cytokines & Receptors, Humoral, Inflammation, Innate, Interleukins, Macrophage Functions, NK Cell Functions, Pathogen Response, T-Cell Functions
Lcn2	3.89	0.186	3.53, 4.26	1.28E-09	Apoptosis, Innate, Transporter Functions
Ccl7	3.79	0.301	3.20, 4.38	7.59E-08	Adaptive, Cancer Progression, Chemokines & Receptors, Cytokines & Receptors, Humoral, Inflammation, NK Cell Functions, T-Cell Functions
Cxcl10	3.75	0.248	3.26, 4.23	1.42E-08	Adaptive, Chemokines & Receptors, Cytokines & Receptors, Inflammation, Innate, Leukocyte Functions
Cxcl1	3.65	0.211	3.23, 4.06	3.99E-09	Adaptive, Chemokines & Receptors, Cytokines & Receptors, Inflammation, Transporter Functions
Oasl1	3.41	0.299	2.82, 4.00	1.97E-07	Basic Cell Functions
Cxcl3	3.36	1.090	1.22, 5.50	1.45E-02	Adaptive, Chemokines & Receptors, Cytokines & Receptors, Inflammation
Ccl3	3.32	0.206	2.92, 3.72	7.29E-09	Adaptive, Chemokines & Receptors, Cytokines & Receptors, Humoral, Inflammation, NK Cell Functions, T-Cell Functions, Transporter Functions
Cxcl9	3.27	0.379	2.53, 4.01	2.78E-06	Adaptive, Chemokines & Receptors, Cytokines & Receptors, Inflammation, Innate
Il1rn	3.07	0.168	2.74, 3.40	3.34E-09	Cytokines & Receptors, Inflammation, Interleukins
C3ar1	2.92	0.166	2.59, 3.24	3.34E-09	Adaptive, Cancer Progression, Complement Pathway, Inflammation, Macrophage Functions
Ccl8	2.90	0.363	2.18, 3.61	5.82E-06	Adaptive, Cancer Progression, Chemokines & Receptors, Cytokines & Receptors, Inflammation
Irf7	2.86	0.273	2.32, 3.39	4.54E-07	Adaptive, Innate, Interferon, Senescence
Msr1	2.83	0.194	2.45, 3.21	1.72E-08	CD molecules, Macrophage Functions, Transporter Functions
Isg15	2.70	0.213	2.28, 3.11	7.12E-08	Basic Cell Functions, Innate, Interferon
Ccl12	2.69	0.191	2.31, 3.06	2.29E-08	Adaptive, Chemokines & Receptors, Cytokines & Receptors, Humoral, Inflammation
Trem2	2.60	0.107	2.39, 2.81	5.11E-10	Basic Cell Functions, Humoral
Oas2	2.58	0.242	2.11, 3.06	3.87E-07	Basic Cell Functions, Interferon

Ifi44	2.55	0.222	2.12, 2.99	1.86E-07	Interferon
Clec5a	2.51	0.130	2.26, 2.77	2.19E-09	Apoptosis, Cytokines & Receptors, Innate
Zbp1	2.45	0.182	2.10, 2.81	3.49E-08	Innate
Cfb	2.44	0.180	2.09, 2.8	3.31E-08	Complement Pathway, Innate
Ccr5	2.26	0.159	1.95, 2.57	2.21E-08	Adaptive, CD molecules, Chemokines & Receptors, Cytokines & Receptors, Dendritic Cell Functions, Macrophage Functions, T-Cell Functions, Transporter Functions
Spp1	2.18	0.123	1.94, 2.42	3.34E-09	Adhesion, Apoptosis, Cytokines & Receptors, T-Cell Functions
Fcgr1	2.13	0.172	1.80, 2.47	8.74E-08	Antigen Processing, CD molecules, Innate, MHC, Transporter Functions
Ifit1	2.12	0.175	1.77, 2.46	1.18E-07	Interferon, Transporter Functions
Mx2	2.11	0.190	1.74, 2.48	2.59E-07	Innate
Clec4n	2.09	0.102	1.89, 2.28	1.28E-09	Cytokines & Receptors, Innate
C1qa	2.09	0.129	1.84, 2.34	7.29E-09	Complement Pathway, Innate
Cxcl13	2.08	0.315	1.47, 2.70	3.38E-05	Adaptive, B-Cell Functions, Chemokines & Receptors, Cytokines & Receptors, Humoral, Inflammation, T-Cell Functions
C1qb	2.02	0.140	1.75, 2.30	1.89E-08	Complement Pathway, Innate
Marco	1.92	0.229	1.47, 2.36	3.65E-06	Innate, Transporter Functions
Csf2	1.90	0.162	1.58, 2.22	1.52E-07	Adaptive, Cytokines & Receptors, Innate, Interleukins, Macrophage Functions, T-Cell Functions, Transporter Functions
Fcgr2b	1.89	0.129	1.63, 2.14	1.72E-08	Antigen Processing, B-Cell Functions, CD molecules, Inflammation, Interleukins, MHC, Transporter Functions
Siglec1	1.88	0.206	1.48, 2.28	1.60E-06	Adhesion, CD molecules, Transporter Functions
Il1a	1.83	0.126	1.58, 2.07	1.79E-08	Adaptive, Cytokines & Receptors, Inflammation, Innate, Interleukins
Slamf7	1.79	0.193	1.42, 2.17	1.33E-06	CD molecules, Innate, NK Cell Functions
Ccl9	1.78	0.100	1.58, 1.97	3.34E-09	Chemokines & Receptors, Cytokines & Receptors
Cxcr1	1.74	0.224	1.30, 2.18	7.34E-06	Adaptive, CD molecules, Chemokines & Receptors, Cytokines & Receptors, Dendritic Cell Functions, Inflammation, Interleukins
Cd14	1.73	0.142	1.45, 2.01	1.09E-07	CD molecules, Cytokines & Receptors, Inflammation, Innate, Pathogen Response, Transporter Functions
Slc11a1	1.70	0.110	1.48, 1.91	1.17E-08	Adaptive, Antigen Processing, Transporter Functions
Usp18	1.70	0.196	1.32, 2.09	2.67E-06	Basic Cell Functions

Ctss	1.69	0.100	1.50, 1.89	4.42E-09	Innate
Cd68	1.67	0.081	1.51, 1.82	1.28E-09	CD molecules
Ccl6	1.64	0.110	1.42, 1.85	1.58E-08	Chemokines & Receptors, Cytokines & Receptors
Cd74	1.00	0.085	0.836, 1.17	1.46E-07	Antigen Processing, B-Cell Functions, CD molecules, Innate, MHC, T-Cell Functions
Fcgr4	1.64	0.123	1.40, 1.88	3.63E-08	CD molecules, T-Cell Functions
Ifit3	1.63	0.148	1.34, 1.92	2.64E-07	Interferon
Arg2	1.58	0.141	1.30, 1.86	2.39E-07	Basic Cell Functions
Tlr2	1.56	0.106	1.35, 1.77	1.72E-08	CD molecules, Inflammation, Innate, Leukocyte Functions, TLR
Ctla4	1.54	0.220	1.11, 1.97	2.03E-05	B-Cell Functions, CD molecules, T-Cell Functions
Ccr1	1.52	0.149	1.23, 1.81	5.69E-07	Adaptive, CD molecules, Chemokines & Receptors, Cytokines & Receptors, Dendritic Cell Functions, Inflammation, Innate, Leukocyte Functions, Transporter Functions
Blnk	1.52	0.174	1.18, 1.86	2.53E-06	B-Cell Functions, Humoral
Plau	1.51	0.103	1.31, 1.71	1.72E-08	Adhesion, Senescence
Irf4	1.48	0.214	1.06, 1.90	2.21E-05	Interleukins, T-Cell Functions, TLR
Il2ra	1.45	0.173	1.11, 1.79	3.65E-06	Adaptive, B-Cell Functions, CD molecules, Inflammation, T-Cell Functions
Rsad2	1.43	0.248	0.947, 1.92	1.15E-04	Basic Cell Functions
Ccr6	1.42	0.274	0.882, 1.95	3.07E-04	Adaptive, CD molecules, Chemokines & Receptors, Humoral, Innate, T-Cell Functions
Cd200r1	1.41	0.083	1.25, 1.57	4.25E-09	B-Cell Functions
Cx3cr1	1.41	0.120	1.18, 1.65	1.48E-07	Chemokines & Receptors, Cytokines & Receptors, Macrophage Functions, Microglial Functions
Il21r	1.39	0.201	0.995, 1.78	2.23E-05	CD molecules, Cytokines & Receptors
Lgals3	1.36	0.095	1.17, 1.54	1.96E-08	Innate, T-Cell Functions
Fcer1g	1.34	0.079	1.18, 1.49	4.25E-09	Antigen Processing, Interleukins, MHC, Transporter Functions
Clec7a	1.34	0.108	1.13, 1.55	8.74E-08	Inflammation, Innate, Leukocyte Functions, Transporter Functions
Itgam	1.34	0.119	1.11, 1.57	2.14E-07	Adaptive, CD molecules, Innate, Leukocyte Functions, T-Cell Functions, Transporter Functions
Csf2rb	1.33	0.089	1.16, 1.51	1.58E-08	CD molecules, Cancer Progression, Cytokines & Receptors

Cxcr3	1.31	0.208	0.901, 1.71	5.27E-05	Adaptive, CD molecules, Chemokines & Receptors, Cytokines & Receptors, Innate, T-Cell Functions
Bst2	1.30	0.132	1.04, 1.56	8.07E-07	CD molecules, Humoral, Innate
Ada	1.27	0.139	1.00, 1.55	1.54E-06	Adhesion, B-Cell Functions, T-Cell Functions
Tnfrsf11a	1.26	0.109	1.05, 1.48	1.64E-07	Adaptive, CD molecules, Cytokines & Receptors, Interleukins, TNF Superfamily
Ly9	1.26	0.130	1.00, 1.51	9.40E-07	Adhesion, CD molecules
Cmpk2	1.00	0.102	0.80, 1.20	8.56E-07	Basic Cell Functions
Icos	1.25	0.154	0.949, 1.55	4.94E-06	CD molecules, T-Cell Functions
Cd83	1.24	0.118	1.01, 1.48	4.32E-07	B-Cell Functions, CD molecules, Dendritic Cell Functions, Humoral, Interleukins, T-Cell Functions
Emr1	1.23	0.088	1.06, 1.41	2.45E-08	Basic Cell Functions
Cd274	1.23	0.096	1.05, 1.42	5.96E-08	CD molecules, T-Cell Functions
Tap1	1.23	0.125	0.987, 1.48	7.71E-07	Antigen Processing, MHC, T-Cell Functions, Transporter Functions
Fcgr3	1.22	0.087	1.04, 1.39	2.45E-08	Antigen Processing, CD molecules, MHC, Transporter Functions
Clec4a2	1.22	0.101	1.02, 1.41	1.20E-07	Innate
Cd180	1.22	0.107	1.01, 1.43	1.93E-07	CD molecules, Inflammation, Innate
Il1b	1.22	0.144	0.936, 1.50	3.30E-06	Adaptive, Chemokines & Receptors, Humoral, Inflammation, Innate, Interleukins, Macrophage Functions, Pathogen Response, T-Cell Functions, Transporter Functions
Ddx60	1.22	0.164	0.897, 1.54	1.12E-05	Basic Cell Functions
Slamf6	1.22	0.171	0.884, 1.55	1.71E-05	CD molecules
Cd86	1.19	0.101	0.992, 1.39	1.52E-07	Adaptive, B-Cell Functions, CD molecules, Dendritic Cell Functions, T-Cell Functions, TLR
H2-K1	1.19	0.116	0.964, 1.42	5.46E-07	Antigen Processing, MHC, T-Cell Functions
Nlrc5	1.19	0.145	0.905, 1.47	4.57E-06	Innate, Interferon, MHC
Ncf4	1.18	0.109	0.965, 1.39	3.61E-07	Basic Cell Functions, Innate
Herc6	1.18	0.144	0.899, 1.46	4.57E-06	Basic Cell Functions
Chil3	1.14	0.362	0.432, 1.85	1.18E-02	Basic Cell Functions
Itgax	1.13	0.093	0.953, 1.32	1.00E-07	Adhesion, CD molecules, Innate, T-Cell Functions

Ikbke	1.07	0.122	0.836, 1.31	2.28E-06	Apoptosis, Innate
Cfp	1.07	0.146	0.781, 1.35	1.33E-05	Cancer Progression, Complement Pathway, Innate
Psmb9	1.06	0.105	0.851, 1.26	6.64E-07	Antigen Processing
Itgb2	1.05	0.059	0.937, 1.17	3.34E-09	CD molecules, Humoral, Inflammation, Leukocyte Functions, NK Cell Functions, T-Cell Functions, Transporter Functions
Cybb	1.05	0.076	0.90, 1.20	2.67E-08	Innate, Transporter Functions
C3	1.05	0.090	0.875, 1.23	1.52E-07	Cancer Progression, Complement Pathway, Inflammation, Innate, Transporter Functions
Psmb8	1.05	0.092	0.867, 1.23	1.97E-07	Antigen Processing
Ly86	1.05	0.099	0.859, 1.25	3.87E-07	Humoral, Inflammation, Innate, Pathogen Response
Sh2b2	1.05	0.093	0.867, 1.23	4.06E-07	B-Cell Functions, Cytokines & Receptors
Stat1	1.05	0.110	0.834, 1.26	1.02E-06	Adaptive, Cytokines & Receptors, Innate, T-Cell Functions
Cd80	1.05	0.152	0.753, 1.35	2.20E-05	Adaptive, CD molecules, T-Cell Functions
Pik3cg	1.04	0.113	0.820, 1.26	1.45E-06	Apoptosis, Inflammation, Innate
Ebi3	1.03	0.123	0.788, 1.27	3.65E-06	Cytokines & Receptors, Humoral, Interleukins
Batf	1.02	0.102	0.817, 1.22	7.09E-07	
Xaf1	1.02	0.125	0.774, 1.27	4.85E-06	Apoptosis
Tlr1	1.02	0.155	0.712, 1.32	3.68E-05	CD molecules, Inflammation, Innate, Interleukins, Macrophage Functions, TLR
Ccl19	1.01	0.164	0.689, 1.33	6.63E-05	Chemokines & Receptors, Cytokines & Receptors, Dendritic Cell Functions, Inflammation, Leukocyte Functions, T-Cell Functions
Itga2b	-1.14	0.192	-1.51, -0.759	9.37E-05	Adhesion, CD molecules, Leukocyte Functions
Ppbp	-1.40	0.282	-1.95, -0.843	4.54E-04	Chemokines & Receptors
Camp	-1.47	0.392	-2.24, -0.703	3.90E-03	Adaptive, Cancer Progression, Inflammation, Innate
Hamp	-1.57	0.236	-2.03, -1.11	3.19E-05	Basic Cell Functions, Innate
Cfd	-2.08	0.628	-3.31, -0.849	8.83E-03	Complement Pathway, Innate

Note: Values shown are the calculated mean $\log_2$ ratios, SEM and 95% confidence intervals of transcript counts for lung tissues of $cSiO_2$-treated mice with respect to time-matched vehicle-treated mice 9 weeks post instillation. Statistical analysis was performed using nSolver software (nanoString) with the Benjamini-Hochberg method for control of false discoveries (α=0.05); FDR-corrected q values are shown. Pathway associations for each significant gene are also shown.

Supplementary Table 6. Significant differentially expressed genes in lung tissues of mice at 13 weeks post $cSiO_2$ instillation (experiment 3).

Gene symbol	Log_2 ratio $cSiO_2$/VEH	SEM	95% CI	FDR *q* value	Associated pathways
Cxcl5	5.56	0.332	4.91, 6.21	6.37E-09	Chemokines & Receptors, Cytokines & Receptors, Inflammation, Leukocyte Functions
Cxcl3	5.42	0.420	4.59, 6.24	6.46E-08	Adaptive, Chemokines & Receptors, Cytokines & Receptors, Inflammation
Ccl2	4.64	0.203	4.24, 5.03	5.77E-10	Chemokines & Receptors, Cytokines & Receptors, Humoral, Inflammation, Innate, Interleukins, Macrophage Functions, NK Cell Functions, Pathogen Response, T-Cell Functions
Ccl7	4.09	0.229	3.64, 4.54	4.18E-09	Adaptive, Cancer Progression, Chemokines & Receptors, Cytokines & Receptors, Humoral, Inflammation, NK Cell Functions, T-Cell Functions
Ccl8	3.80	0.353	3.10, 4.49	3.98E-07	Adaptive, Cancer Progression, Chemokines & Receptors, Cytokines & Receptors, Inflammation
Lcn2	3.79	0.134	3.52, 4.05	6.32E-11	Apoptosis, Innate, Transporter Functions
Cxcl10	3.67	0.351	2.98, 4.36	5.37E-07	Adaptive, Chemokines & Receptors, Cytokines & Receptors, Inflammation, Innate, Leukocyte Functions
Cxcl1	3.62	0.216	3.19, 4.04	6.37E-09	Adaptive, Chemokines & Receptors, Cytokines & Receptors, Inflammation, Transporter Functions
Cxcl9	3.40	0.366	2.68, 4.11	1.69E-06	Adaptive, Chemokines & Receptors, Cytokines & Receptors, Inflammation, Innate
Ccl3	3.33	0.177	2.98, 3.68	2.83E-09	Adaptive, Chemokines & Receptors, Cytokines & Receptors, Humoral, Inflammation, NK Cell Functions, T-Cell Functions, Transporter Functions
Ccl12	3.31	0.218	2.88, 3.74	1.28E-08	Adaptive, Chemokines & Receptors, Cytokines & Receptors, Humoral, Inflammation
Cxcl13	3.23	0.320	2.60, 3.86	7.47E-07	Adaptive, B-Cell Functions, Chemokines & Receptors, Cytokines & Receptors, Humoral, Inflammation, T-Cell Functions
C1qa	3.06	0.266	2.54, 3.58	2.11E-07	Complement Pathway, Innate
Pdcd1	3.01	0.351	2.32, 3.70	3.40E-06	Apoptosis, CD molecules, Humoral, T-Cell Functions
C3ar1	2.98	0.218	2.56, 3.41	3.49E-08	Adaptive, Cancer Progression, Complement Pathway, Inflammation, Macrophage Functions
Il1rn	2.90	0.133	2.63, 3.16	7.57E-10	Cytokines & Receptors, Inflammation, Interleukins
C1qb	2.84	0.212	2.43, 3.26	4.50E-08	Complement Pathway, Innate
Oasl1	2.77	0.313	2.16, 3.38	2.56E-06	Basic Cell Functions

Ccr5	2.74	0.257	2.24, 3.25	4.31E-07	Adaptive, CD molecules, Chemokines & Receptors, Cytokines & Receptors, Dendritic Cell Functions, Macrophage Functions, T-Cell Functions, Transporter Functions
Trem2	2.46	0.160	2.15, 2.77	1.12E-08	Basic Cell Functions, Humoral
Msr1	2.45	0.134	2.18, 2.71	3.60E-09	CD molecules, Macrophage Functions, Transporter Functions
Irf7	2.43	0.277	1.88, 2.97	2.80E-06	Adaptive, Innate, Interferon, Senescence
Cfb	2.31	0.148	2.02, 2.60	9.84E-09	Complement Pathway, Innate
Isg15	2.28	0.233	1.82, 2.74	1.08E-06	Basic Cell Functions, Innate, Interferon
Pou2af1	2.25	0.313	1.63, 2.86	1.94E-05	Basic Cell Functions, Humoral
Fcgr4	2.23	0.205	1.83, 2.63	3.66E-07	CD molecules, T-Cell Functions
Slamf7	2.21	0.241	1.74, 2.69	1.83E-06	CD molecules, Innate, NK Cell Functions
Spp1	2.16	0.190	1.79, 2.53	2.28E-07	Adhesion, Apoptosis, Cytokines & Receptors, T-Cell Functions
Clec5a	2.12	0.127	1.87, 2.37	6.37E-09	Apoptosis, Cytokines & Receptors, Innate
Ccr6	2.10	0.294	1.52, 2.67	2.02E-05	Adaptive, CD molecules, Chemokines & Receptors, Humoral, Innate, T-Cell Functions
Zbp1	2.09	0.192	1.71, 2.47	3.56E-07	Innate
Fcgr1	2.08	0.216	1.66, 2.50	1.22E-06	Antigen Processing, CD molecules, Innate, MHC, Transporter Functions
Oas2	2.04	0.222	1.60, 2.47	1.81E-06	Basic Cell Functions, Interferon
Clec4n	2.03	0.123	1.79, 2.28	6.37E-09	Cytokines & Receptors, Innate
Blnk	2.00	0.232	1.55, 2.46	3.31E-06	B-Cell Functions, Humoral
Marco	1.97	0.226	1.52, 2.41	2.93E-06	Innate, Transporter Functions
Ctss	1.96	0.177	1.62, 2.31	2.97E-07	Innate
Ifi44	1.95	0.228	1.50, 2.39	3.62E-06	Interferon
Cd14	1.89	0.108	1.67, 2.10	4.68E-09	CD molecules, Cytokines & Receptors, Inflammation, Innate, Pathogen Response, Transporter Functions
Chil3	1.87	0.553	0.781, 2.95	8.45E-03	Basic Cell Functions
Fcgr2b	1.86	0.093	1.67, 2.04	1.49E-09	Antigen Processing, B-Cell Functions, CD molecules, Inflammation, Interleukins, MHC, Transporter Functions
Csf2	1.82	0.189	1.45, 2.19	1.27E-06	Adaptive, Cytokines & Receptors, Innate, Interleukins, Macrophage Functions, T-Cell Functions, Transporter Functions

Il21r	1.81	0.269	1.28, 2.34	3.55E-05	CD molecules, Cytokines & Receptors
Ifit1	1.77	0.201	1.38, 2.17	2.69E-06	Interferon, Transporter Functions
Ada	1.76	0.208	1.35, 2.17	3.84E-06	Adhesion, B-Cell Functions, T-Cell Functions
Siglec1	1.75	0.197	1.37, 2.14	2.51E-06	Adhesion, CD molecules, Transporter Functions
Irf4	1.74	0.222	1.31, 2.18	8.33E-06	Interleukins, T-Cell Functions, TLR
Tnfrsf13c	1.74	0.287	1.18, 2.30	9.52E-05	B-Cell Functions, CD molecules, T-Cell Functions, TNF Superfamily
Arg1	1.73	0.475	0.796, 2.66	5.21E-03	Basic Cell Functions
Slc11a1	1.70	0.166	1.38, 2.03	6.61E-07	Adaptive, Antigen Processing, Transporter Functions
Cxcr3	1.66	0.204	1.26, 2.06	5.76E-06	Adaptive, CD molecules, Chemokines & Receptors, Cytokines & Receptors, Innate, T-Cell Functions
Arg2	1.64	0.109	1.43, 1.85	1.28E-08	Basic Cell Functions
Clec4a2	1.62	0.159	1.31, 1.93	6.74E-07	Innate
Ccl9	1.60	0.099	1.41, 1.80	7.45E-09	Chemokines & Receptors, Cytokines & Receptors
Cd68	1.59	0.079	1.44, 1.75	1.47E-09	CD molecules
Slamf6	1.59	0.239	1.12, 2.06	3.95E-05	CD molecules
Il1b	1.58	0.140	1.31, 1.86	2.46E-07	Adaptive, Chemokines & Receptors, Humoral, Inflammation, Innate, Interleukins, Macrophage Functions, Pathogen Response, T-Cell Functions, Transporter Functions
Ctla4	1.58	0.226	1.13, 2.02	2.59E-05	B-Cell Functions, CD molecules, T-Cell Functions
Plau	1.56	0.126	1.31, 1.80	9.38E-08	Adhesion, Senescence
Usp18	1.52	0.180	1.17, 1.88	3.95E-06	Basic Cell Functions
Tnfrsf4	1.49	0.161	1.17, 1.80	1.69E-06	B-Cell Functions, CD molecules, Cytokines & Receptors, Inflammation, T-Cell Functions, TNF Superfamily
Ccr1	1.48	0.092	1.30, 1.67	7.47E-09	Adaptive, CD molecules, Chemokines & Receptors, Cytokines & Receptors, Dendritic Cell Functions, Inflammation, Innate, Leukocyte Functions, Transporter Functions
Cd200r1	1.45	0.097	1.26, 1.64	1.32E-08	B-Cell Functions
Cd83	1.44	0.116	1.21, 1.67	8.89E-08	B-Cell Functions, CD molecules, Dendritic Cell Functions, Humoral, Interleukins, T-Cell Functions
Il1a	1.41	0.145	1.12, 1.69	1.11E-06	Adaptive, Cytokines & Receptors, Inflammation, Innate, Interleukins

Tlr9	1.41	0.222	0.972, 1.84	6.32E-05	CD molecules, Chemokines & Receptors, Inflammation, Innate, Interleukins, TLR, Transporter Functions
Cfp	1.40	0.176	1.05, 1.74	7.21E-06	Cancer Progression, Complement Pathway, Innate
Tlr2	1.37	0.086	1.20, 1.54	7.82E-09	CD molecules, Inflammation, Innate, Leukocyte Functions, TLR
Tap1	1.36	0.146	1.07, 1.64	1.69E-06	Antigen Processing, MHC, T-Cell Functions, Transporter Functions
Mx2	1.36	0.293	0.782, 1.93	9.36E-04	Innate
Ikbke	1.34	0.134	1.08, 1.61	8.34E-07	Apoptosis, Innate
Cd19	1.34	0.285	0.782, 1.90	8.22E-04	B-Cell Functions, CD molecules
Emr1	1.33	0.103	1.13, 1.54	6.46E-08	Basic Cell Functions
H2-K1	1.33	0.126	1.08, 1.58	5.00E-07	Antigen Processing, MHC, T-Cell Functions
Cd74	1.33	0.142	1.05, 1.61	1.58E-06	Antigen Processing, B-Cell Functions, CD molecules, Innate, MHC, T-Cell Functions
Socs3	1.33	0.149	1.03, 1.62	2.56E-06	T-Cell Functions
Cd80	1.32	0.096	1.13, 1.51	3.15E-08	Adaptive, CD molecules, T-Cell Functions
Itgax	1.31	0.089	1.13, 1.48	1.51E-08	Adhesion, CD molecules, Innate, T-Cell Functions
Cd274	1.31	0.099	1.11, 1.50	4.95E-08	CD molecules, T-Cell Functions
Cd86	1.31	0.105	1.10, 1.52	8.75E-08	Adaptive, B-Cell Functions, CD molecules, Dendritic Cell Functions, T-Cell Functions, TLR
Fpr2	1.30	0.078	1.14, 1.45	6.37E-09	Adaptive, Basic Cell Functions, Inflammation
Ccl6	1.30	0.106	1.09, 1.51	1.04E-07	Chemokines & Receptors, Cytokines & Receptors
Il2ra	1.30	0.158	0.988, 1.61	5.51E-06	Adaptive, B-Cell Functions, CD molecules, Inflammation, T-Cell Functions
Ccl19	1.30	0.176	0.959, 1.65	1.46E-05	Chemokines & Receptors, Cytokines & Receptors, Dendritic Cell Functions, Inflammation, Leukocyte Functions, T-Cell Functions
Lgals3	1.27	0.072	1.13, 1.42	4.23E-09	Innate, T-Cell Functions
Csf2rb	1.26	0.083	1.10, 1.43	1.20E-08	CD molecules, Cancer Progression, Cytokines & Receptors
Ncf4	1.26	0.099	1.07, 1.46	6.72E-08	Basic Cell Functions, Innate
Cx3cr1	1.25	0.129	0.994, 1.50	1.22E-06	Chemokines & Receptors, Cytokines & Receptors, Macrophage Functions, Microglial Functions
Ly9	1.25	0.173	0.912, 1.59	1.78E-05	Adhesion, CD molecules
Fcer1g	1.23	0.095	1.05, 1.42	6.36E-08	Antigen Processing, Interleukins, MHC, Transporter Functions

Psmb8	1.23	0.142	0.949, 1.50	3.13E-06	Antigen Processing
C3	1.22	0.097	1.03, 1.41	8.12E-08	Cancer Progression, Complement Pathway, Inflammation, Innate, Transporter Functions
Ly86	1.21	0.177	0.859, 1.55	3.19E-05	Humoral, Inflammation, Innate, Pathogen Response
Ccl22	1.21	0.183	0.854, 1.57	4.17E-05	Chemokines & Receptors, Cytokines & Receptors, Humoral, Inflammation
Tlr1	1.21	0.248	0.728, 1.70	6.04E-04	CD molecules, Inflammation, Innate, Interleukins, Macrophage Functions, TLR
Ccl11	1.20	0.216	0.78, 1.63	2.04E-04	Adaptive, Cancer Progression, Chemokines & Receptors, Cytokines & Receptors, Inflammation, T-Cell Functions
Nlrc5	1.19	0.163	0.867, 1.50	1.66E-05	Innate, Interferon, MHC
H2-DMb2	1.18	0.188	0.809, 1.55	7.18E-05	Antigen Processing, MHC
H2-Ob	1.18	0.232	0.724, 1.63	4.54E-04	Antigen Processing, MHC
Batf	1.17	0.123	0.929, 1.41	1.33E-06	
Tnfrsf11a	1.15	0.076	1.00, 1.30	1.28E-08	Adaptive, CD molecules, Cytokines & Receptors, Interleukins, TNF Superfamily
Bst2	1.14	0.127	0.890, 1.39	2.35E-06	CD molecules, Humoral, Innate
Cd180	1.13	0.169	0.801, 1.46	3.69E-05	CD molecules, Inflammation, Innate
Psmb9	1.11	0.124	0.868, 1.35	2.28E-06	Antigen Processing
Ifit3	1.11	0.190	0.737, 1.48	1.36E-04	Interferon
Itgam	1.10	0.161	0.787, 1.42	3.10E-05	Adaptive, CD molecules, Innate, Leukocyte Functions, T-Cell Functions, Transporter Functions
Ms4a1	1.10	0.217	0.670, 1.52	4.72E-04	B-Cell Functions, CD molecules, Humoral
Fcgr3	1.09	0.082	0.931, 1.25	4.60E-08	Antigen Processing, CD molecules, MHC, Transporter Functions
Tnfrsf13b	1.09	0.154	0.785, 1.39	2.26E-05	B-Cell Functions, CD molecules, TNF Superfamily
Tnfaip3	1.08	0.105	0.87, 1.28	6.62E-07	Apoptosis, B-Cell Functions, Inflammation, Innate
Cd53	1.06	0.097	0.874, 1.25	3.35E-07	CD molecules
Socs1	1.06	0.148	0.771, 1.35	1.97E-05	Cytokines & Receptors, T-Cell Functions
Icos	1.05	0.164	0.732, 1.38	5.72E-05	CD molecules, T-Cell Functions
Pou2f2	1.04	0.133	0.783, 1.31	8.33E-06	Humoral, T-Cell Functions
Cxcl16	1.03	0.084	0.862, 1.19	1.06E-07	Chemokines & Receptors, Cytokines & Receptors, Innate, Interferon

H2-D1	1.01	0.093	0.827, 1.19	3.77E-07	Antigen Processing, MHC, T-Cell Functions
H2-Ea-ps	1.01	0.113	0.786, 1.23	2.51E-06	Antigen Processing
Klrd1	-1.04	0.109	-1.26, -0.828	1.29E-06	NK Cell Functions
Kdr	-1.06	0.167	-1.39, -0.733	6.27E-05	Adhesion, Apoptosis, Cancer Progression
Lrrn3	-1.13	0.100	-1.33, -0.939	2.28E-07	CD molecules
Itga2b	-1.13	0.312	-1.74, -0.516	5.39E-03	Adhesion, CD molecules, Leukocyte Functions
Txk	-1.14	0.124	-1.38, -0.897	1.83E-06	Adaptive, Cytokines & Receptors, Interleukins, T-Cell Functions
Angpt2	-1.19	0.177	-1.53, -0.838	3.82E-05	Adhesion, Cancer Progression
Il11ra1	-1.29	0.246	-1.77, -0.806	3.49E-04	Cytokines & Receptors, Interleukins, NK Cell Functions
Ppbp	-1.44	0.364	-2.16, -0.730	2.91E-03	Chemokines & Receptors
Camp	-1.56	0.475	-2.49, -0.625	1.01E-02	Adaptive, Cancer Progression, Inflammation, Innate
Klrb1c	-1.57	0.200	-1.97, -1.18	8.11E-06	NK Cell Functions
Hamp	-2.08	0.576	-3.21, -0.949	5.43E-03	Basic Cell Functions, Innate
Cfd	-2.84	0.694	-4.20, -1.48	2.34E-03	Complement Pathway, Innate

Note: Values shown are the calculated mean log_2 ratios, SEM and 95% confidence intervals of transcript counts for lung tissues of $cSiO_2$-treated mice with respect to time-matched vehicle-treated mice 13 weeks post instillation. Statistical analysis was performed using nSolver software (nanoString) with the Benjamini-Hochberg method for control of false discoveries (α=0.05); FDR-corrected q values are shown. Pathway associations for each significant gene are also shown.

Supplementary Table 7. Significant differentially expressed genes in kidney tissues of mice at 13 weeks post $cSiO_2$ instillation (experiment 3).

Gene symbol	Log$_2$ ratio $cSiO_2$/VEH	SEM	95% CI	FDR *q* value	Associated pathways
Fpr2	4.68	0.460	3.78, 5.58	3.11E-05	Adaptive, Basic Cell Functions, Inflammation
Lcn2	4.38	0.682	3.04, 5.71	1.45E-04	Apoptosis, Innate, Transporter Functions
Itgam	4.09	0.468	3.17, 5.00	4.21E-05	Adaptive, CD molecules, Innate, Leukocyte Functions, T-Cell Functions, Transporter Functions
Clec4a2	3.81	0.496	2.84, 4.79	4.42E-05	Innate
Ccl12	3.75	0.457	2.85, 4.64	4.21E-05	Adaptive, Chemokines & Receptors, Cytokines & Receptors, Humoral, Inflammation
Fcgr4	3.60	0.394	2.83, 4.37	3.11E-05	CD molecules, T-Cell Functions
Ccl5	3.36	0.500	2.38, 4.34	1.01E-04	Adaptive, Cancer Progression, Chemokines & Receptors, Dendritic Cell Functions, Inflammation, Innate, Macrophage Functions, NK Cell Functions, T-Cell Functions, Transporter Functions
Tlr8	3.33	0.426	2.50, 4.17	4.21E-05	CD molecules, Inflammation, Innate, Interleukins, Pathogen Response, TLR
Fcgr1	3.31	0.388	2.55, 4.07	4.21E-05	Antigen Processing, CD molecules, Innate, MHC, Transporter Functions
C3ar1	3.21	0.386	2.45, 3.97	4.21E-05	Adaptive, Cancer Progression, Complement Pathway, Inflammation, Macrophage Functions
Ccr5	3.13	0.530	2.09, 4.17	2.40E-04	Adaptive, CD molecules, Chemokines & Receptors, Cytokines & Receptors, Dendritic Cell Functions, Macrophage Functions, T-Cell Functions, Transporter Functions
Klra2	3.09	0.363	2.38, 3.80	4.21E-05	NK Cell Functions
Ccl2	3.05	0.371	2.33, 3.78	4.21E-05	Chemokines & Receptors, Cytokines & Receptors, Humoral, Inflammation, Innate, Interleukins, Macrophage Functions, NK Cell Functions, Pathogen Response, T-Cell Functions
Il1b	2.99	0.308	2.39, 3.59	3.11E-05	Adaptive, Chemokines & Receptors, Humoral, Inflammation, Innate, Interleukins, Macrophage Functions, Pathogen Response, T-Cell Functions, Transporter Functions
Ccl9	2.95	0.379	2.21, 3.69	4.21E-05	Chemokines & Receptors, Cytokines & Receptors
Irf7	2.89	0.371	2.17, 3.62	4.21E-05	Adaptive, Innate, Interferon, Senescence
Col1a1	2.89	0.519	1.88, 3.91	3.35E-04	Cancer Progression, Transporter Functions
Hck	2.84	0.308	2.23, 3.44	3.11E-05	Inflammation, Innate
Oasl1	2.83	0.434	1.97, 3.68	1.32E-04	Basic Cell Functions

Ctss	2.80	0.370	2.07, 3.52	4.84E-05	Innate
C1qb	2.73	0.347	2.05, 3.41	4.21E-05	Complement Pathway, Innate
Oas2	2.71	0.329	2.07, 3.36	4.21E-05	Basic Cell Functions, Interferon
Zbp1	2.68	0.338	2.02, 3.35	4.21E-05	Innate
Fcgr3	2.67	0.362	1.96, 3.38	6.24E-05	Antigen Processing, CD molecules, MHC, Transporter Functions
Cxcl9	2.67	0.450	1.79, 3.55	2.34E-04	Adaptive, Chemokines & Receptors, Cytokines & Receptors, Inflammation, Innate
Pou2f2	2.66	0.346	1.98, 3.34	4.42E-05	Humoral, T-Cell Functions
C1qa	2.65	0.338	1.99, 3.31	4.21E-05	Complement Pathway, Innate
Ccl28	2.64	0.524	1.61, 3.67	7.19E-04	Chemokines & Receptors, Cytokines & Receptors
Lif	2.64	0.870	0.939, 4.35	1.94E-02	Cytokines & Receptors
Cx3cr1	2.63	0.334	1.98, 3.28	4.21E-05	Chemokines & Receptors, Cytokines & Receptors, Macrophage Functions, Microglial Functions
Emr1	2.63	0.373	1.90, 3.37	7.69E-05	Basic Cell Functions
Isg15	2.61	0.358	1.91, 3.31	6.46E-05	Basic Cell Functions, Innate, Interferon
Mx2	2.60	0.356	1.90, 3.30	6.46E-05	Innate
Ccl8	2.59	0.880	0.867, 4.31	2.15E-02	Adaptive, Cancer Progression, Chemokines & Receptors, Cytokines & Receptors, Inflammation
Ifit1	2.57	0.339	1.91, 3.24	4.84E-05	Interferon, Transporter Functions
Cxcl10	2.57	0.356	1.88, 3.27	6.46E-05	Adaptive, Chemokines & Receptors, Cytokines & Receptors, Inflammation, Innate, Leukocyte Functions
Cxcl1	2.57	0.539	1.51, 3.63	1.11E-03	Adaptive, Chemokines & Receptors, Cytokines & Receptors, Inflammation, Transporter Functions
Fcer1g	2.52	0.320	1.89, 3.15	4.21E-05	Antigen Processing, Interleukins, MHC, Transporter Functions
Mrc1	2.52	0.403	1.73, 3.31	1.76E-04	CD molecules
H2-DMb2	2.49	0.623	1.27, 3.71	3.64E-03	Antigen Processing, MHC
Socs3	2.49	0.997	0.541, 4.45	4.89E-02	T-Cell Functions
Ncf4	2.46	0.294	1.88, 3.04	4.21E-05	Basic Cell Functions, Innate
C7	2.46	0.424	1.63, 3.29	2.69E-04	Complement Pathway, Innate
Col3a1	2.44	0.451	1.56, 3.33	4.05E-04	Adhesion, Cancer Progression, Cytokines & Receptors

C5ar1	2.41	0.296	1.83, 2.99	4.21E-05	Chemokines & Receptors, Complement Pathway, Humoral
Vcam1	2.41	0.429	1.57, 3.25	3.24E-04	Adhesion, CD molecules, Leukocyte Functions, T-Cell Functions
Cxcl13	2.41	0.570	1.29, 3.53	2.46E-03	Adaptive, B-Cell Functions, Chemokines & Receptors, Cytokines & Receptors, Humoral, Inflammation, T-Cell Functions
Cd84	2.40	0.414	1.59, 3.21	2.69E-04	Adhesion, CD molecules
Ccr2	2.36	0.387	1.61, 3.12	2.06E-04	Adaptive, CD molecules, Cancer Progression, Chemokines & Receptors, Cytokines & Receptors, Dendritic Cell Functions, Humoral, Inflammation, Interleukins, Macrophage Functions, T-Cell Functions
Klra17	2.33	0.428	1.49, 3.17	3.89E-04	NK Cell Functions
Runx1	2.25	0.387	1.49, 3.00	2.64E-04	
Cd48	2.24	0.323	1.6, 2.87	8.56E-05	CD molecules, T-Cell Functions
Cd14	2.24	0.383	1.49, 2.99	2.58E-04	CD molecules, Cytokines & Receptors, Inflammation, Innate, Pathogen Response, Transporter Functions
Ly86	2.21	0.324	1.58, 2.84	9.16E-05	Humoral, Inflammation, Innate, Pathogen Response
Cd68	2.20	0.278	1.66, 2.75	4.21E-05	CD molecules
Cybb	2.17	0.268	1.64, 2.69	4.21E-05	Innate, Transporter Functions
Itgal	2.16	0.233	1.71, 2.62	3.11E-05	Adhesion, CD molecules, Leukocyte Functions, T-Cell Functions
Il10ra	2.16	0.328	1.52, 2.81	1.17E-04	CD molecules
Lyz2	2.15	0.350	1.46, 2.84	2.05E-04	Transporter Functions
Itgb2	2.09	0.266	1.57, 2.61	4.21E-05	CD molecules, Humoral, Inflammation, Leukocyte Functions, NK Cell Functions, T-Cell Functions, Transporter Functions
Nlrc5	2.08	0.369	1.35, 2.80	3.22E-04	Innate, Interferon, MHC
Spn	2.07	0.285	1.52, 2.63	6.46E-05	Adhesion, Apoptosis, CD molecules, T-Cell Functions
Tlr7	2.06	0.336	1.40, 2.72	2.06E-04	Chemokines & Receptors, Inflammation, Innate, Interleukins, Microglial Functions, Pathogen Response, TLR
Ddx60	2.02	0.322	1.39, 2.65	1.76E-04	Basic Cell Functions
Syk	2.01	0.321	1.38, 2.64	1.76E-04	B-Cell Functions, Innate, Interleukins, Leukocyte Functions, Macrophage Functions, T-Cell Functions, Transporter Functions
Ccl20	2.01	0.642	0.753, 3.27	1.64E-02	Chemokines & Receptors, Cytokines & Receptors, Inflammation
Selplg	2.00	0.328	1.36, 2.65	2.06E-04	Adhesion, CD molecules, Leukocyte Functions
Ptprc	1.96	0.289	1.40, 2.53	9.57E-05	B-Cell Functions, CD molecules, Cytokines & Receptors, T-Cell Functions

Ifit3	1.96	0.325	1.32, 2.59	2.18E-04	Interferon
Cxcr6	1.95	0.395	1.17, 2.72	8.51E-04	CD molecules, Chemokines & Receptors, Innate
Spp1	1.94	0.437	1.08, 2.79	1.82E-03	Adhesion, Apoptosis, Cytokines & Receptors, T-Cell Functions
Cd74	1.93	0.339	1.27, 2.60	2.93E-04	Antigen Processing, B-Cell Functions, CD molecules, Innate, MHC, T-Cell Functions
Tap1	1.88	0.285	1.32, 2.44	1.17E-04	Antigen Processing, MHC, T-Cell Functions, Transporter Functions
Usp18	1.88	0.304	1.29, 2.48	1.95E-04	Basic Cell Functions
Psmb8	1.85	0.310	1.25, 2.46	2.21E-04	Antigen Processing
H2-Eb1	1.85	0.328	1.21, 2.49	3.19E-04	Antigen Processing, Interleukins, MHC
C4b	1.85	0.337	1.19, 2.51	3.62E-04	Complement Pathway, Humoral, Inflammation, Innate
Cxcl11	1.84	0.358	1.14, 2.54	6.34E-04	Chemokines & Receptors, Cytokines & Receptors, Inflammation, Innate
Il2rg	1.83	0.253	1.34, 2.33	6.46E-05	B-Cell Functions, CD molecules, Cytokines & Receptors, Interleukins, T-Cell Functions
Cd3g	1.83	0.488	0.871, 2.79	5.62E-03	Apoptosis, CD molecules, T-Cell Functions, Transporter Functions
H2-Aa	1.82	0.335	1.16, 2.48	4.00E-04	Antigen Processing, Interferon, MHC, T-Cell Functions
H2-Ab1	1.80	0.323	1.17, 2.44	3.32E-04	Antigen Processing, Interferon, MHC, T-Cell Functions
H2-K1	1.78	0.306	1.18, 2.38	2.64E-04	Antigen Processing, MHC, T-Cell Functions
Lilra5	1.76	0.247	1.27, 2.24	7.52E-05	CD molecules, Innate
Pik3cd	1.76	0.292	1.18, 2.33	2.18E-04	Inflammation, Innate
C3	1.76	0.414	0.953, 2.57	2.34E-03	Cancer Progression, Complement Pathway, Inflammation, Innate, Transporter Functions
H2-Ea-ps	1.75	0.317	1.13, 2.37	3.48E-04	Antigen Processing
Cxcr3	1.75	0.369	1.03, 2.48	1.11E-03	Adaptive, CD molecules, Chemokines & Receptors, Cytokines & Receptors, Innate, T-Cell Functions
Gbp5	1.74	0.286	1.18, 2.30	2.06E-04	Interferon
Tnfrsf1b	1.73	0.225	1.29, 2.17	4.42E-05	CD molecules, Inflammation, TNF Superfamily
Itga4	1.73	0.238	1.26, 2.20	6.46E-05	Adhesion, CD molecules, Leukocyte Functions
Tnfrsf12a	1.73	0.403	0.938, 2.52	2.24E-03	Adhesion, Apoptosis, CD molecules, TNF Superfamily
Clec7a	1.72	0.350	1.03, 2.40	8.85E-04	Inflammation, Innate, Leukocyte Functions, Transporter Functions

Cd180	1.71	0.306	1.11, 2.31	3.32E-04	CD molecules, Inflammation, Innate
Fn1	1.71	0.391	0.946, 2.48	1.96E-03	Adhesion, Apoptosis
Bst2	1.70	0.245	1.22, 2.18	8.56E-05	CD molecules, Humoral, Innate
H2-DMa	1.70	0.306	1.10, 2.29	3.43E-04	Antigen Processing, MHC, T-Cell Functions
Xaf1	1.69	0.250	1.20, 2.18	9.98E-05	Apoptosis
Psmb9	1.69	0.273	1.15, 2.22	1.99E-04	Antigen Processing
Amica1	1.66	0.312	1.05, 2.27	4.74E-04	Adhesion, Transporter Functions
Cd86	1.65	0.290	1.08, 2.22	2.99E-04	Adaptive, B-Cell Functions, CD molecules, Dendritic Cell Functions, T-Cell Functions, TLR
Casp1	1.64	0.269	1.11, 2.16	2.06E-04	Cytokines & Receptors, Innate, Interleukins, Microglial Functions
H2-DMb1	1.62	0.327	0.984, 2.26	7.96E-04	Antigen Processing, MHC
Lcp1	1.60	0.231	1.15, 2.05	8.56E-05	T-Cell Functions, Transporter Functions
Cdkn1a	1.58	0.339	0.913, 2.24	1.29E-03	B-Cell Functions, Cancer Progression, Cell Cycle, Senescence
Cfp	1.57	0.273	1.03, 2.10	2.82E-04	Cancer Progression, Complement Pathway, Innate
Herc6	1.55	0.304	0.956, 2.15	6.55E-04	Basic Cell Functions
Tnfrsf14	1.51	0.249	1.03, 2.00	2.06E-04	CD molecules, T-Cell Functions, TNF Superfamily
Inpp5d	1.51	0.267	0.985, 2.03	3.13E-04	Apoptosis, B-Cell Functions, Interleukins
Ltb	1.51	0.525	0.485, 2.54	2.40E-02	Cytokines & Receptors, Interleukins, TNF Superfamily
Vim	1.49	0.259	0.980, 1.99	2.82E-04	Cancer Progression
Icam1	1.48	0.229	1.03, 1.93	1.39E-04	Adaptive, Antigen Processing, CD molecules, Leukocyte Functions, T-Cell Functions, Transporter Functions
H2-Ob	1.48	0.334	0.825, 2.14	1.82E-03	Antigen Processing, MHC
Lck	1.44	0.460	0.535, 2.34	1.57E-02	Apoptosis, B-Cell Functions, T-Cell Functions
Cd83	1.43	0.200	1.04, 1.83	7.10E-05	B-Cell Functions, CD molecules, Dendritic Cell Functions, Humoral, Interleukins, T-Cell Functions
Irf1	1.43	0.236	0.970, 1.89	2.06E-04	Interleukins, T-Cell Functions
Ifit2	1.42	0.309	0.816, 2.03	1.41E-03	Interferon
Anxa1	1.42	0.327	0.784, 2.06	2.01E-03	Cell Cycle, Inflammation, T-Cell Functions, Transporter Functions
H2-M3	1.41	0.254	0.911, 1.91	3.43E-04	Antigen Processing, MHC, NK Cell Functions, T-Cell Functions

Clu	1.40	0.382	0.655, 2.15	6.32E-03	Apoptosis, Cancer Progression, Innate
Thy1	1.38	0.347	0.705, 2.06	3.64E-03	Adhesion, CD molecules, T-Cell Functions
Irgm2	1.37	0.193	0.987, 1.74	7.69E-05	Innate, Interferon
Stat1	1.35	0.209	0.946, 1.76	1.35E-04	Adaptive, Cytokines & Receptors, Innate, T-Cell Functions
Csf1	1.34	0.195	0.962, 1.73	8.63E-05	Cytokines & Receptors, Inflammation, Innate, Macrophage Functions, Transporter Functions
H2-D1	1.32	0.224	0.882, 1.76	2.44E-04	Antigen Processing, MHC, T-Cell Functions
Ikbke	1.28	0.240	0.807, 1.75	4.69E-04	Apoptosis, Innate
Serping1	1.27	0.248	0.781, 1.75	6.45E-04	Complement Pathway, Innate
Relb	1.26	0.249	0.772, 1.75	7.01E-04	Antigen Processing, Dendritic Cell Functions, T-Cell Functions
Il34	1.26	0.364	0.546, 1.97	9.07E-03	Cytokines & Receptors, Inflammation, Innate, Macrophage Functions
Psmb10	1.25	0.223	0.813, 1.69	3.27E-04	Humoral, T-Cell Functions
Entpd1	1.22	0.205	0.814, 1.62	2.35E-04	CD molecules
Myc	1.21	0.253	0.717, 1.71	1.06E-03	Apoptosis, Cell Cycle, Senescence
Ada	1.20	0.213	0.783, 1.62	3.17E-04	Adhesion, B-Cell Functions, T-Cell Functions
Cd3d	1.20	0.439	0.336, 2.06	3.22E-02	CD molecules, T-Cell Functions
Stat2	1.19	0.195	0.805, 1.57	2.06E-04	Basic Cell Functions
Tlr4	1.18	0.223	0.739, 1.61	5.01E-04	Adaptive, CD molecules, Chemokines & Receptors, Inflammation, Innate, Interleukins, Pathogen Response, T-Cell Functions, TLR
Tnfrsf11a	1.18	0.268	0.660, 1.71	1.83E-03	Adaptive, CD molecules, Cytokines & Receptors, Interleukins, TNF Superfamily
Tlr2	1.16	0.281	0.607, 1.71	2.89E-03	CD molecules, Inflammation, Innate, Leukocyte Functions, TLR
Axl	1.15	0.210	0.742, 1.57	3.63E-04	Inflammation, Innate, NK Cell Functions, Transporter Functions
Il18r1	1.15	0.418	0.334, 1.97	3.03E-02	CD molecules, Innate, T-Cell Functions
Tnfaip3	1.14	0.194	0.761, 1.52	2.47E-04	Apoptosis, B-Cell Functions, Inflammation, Innate
Irf8	1.13	0.179	0.777, 1.48	1.73E-04	Interferon, Interleukins, T-Cell Functions
Itga5	1.13	0.226	0.686, 1.57	7.68E-04	Adhesion, CD molecules, Innate, Leukocyte Functions
Rel	1.11	0.168	0.778, 1.44	1.18E-04	Cytokines & Receptors, Interleukins, T-Cell Functions

Il16	1.11	0.268	0.581, 1.63	2.85E-03	Chemokines & Receptors, Cytokines & Receptors, Interleukins, Leukocyte Functions
Ifi44	1.10	0.188	0.734, 1.47	2.53E-04	Interferon
C1ra	1.10	0.210	0.692, 1.52	5.20E-04	Complement Pathway, Innate
Isg20	1.09	0.276	0.548, 1.63	3.94E-03	Basic Cell Functions, Innate, Interferon
C1s1	1.08	0.226	0.640, 1.53	1.06E-03	Complement Pathway, Innate
Tlr1	1.07	0.226	0.625, 1.51	1.17E-03	CD molecules, Inflammation, Innate, Interleukins, Macrophage Functions, TLR
Rrad	1.07	0.286	0.508, 1.63	5.70E-03	Basic Cell Functions
Tap2	1.06	0.222	0.629, 1.50	1.06E-03	Antigen Processing, MHC, Transporter Functions
Cd274	1.05	0.176	0.709, 1.40	2.21E-04	CD molecules, T-Cell Functions
Ifi27	1.04	0.169	0.703, 1.37	2.06E-04	Interferon, NK Cell Functions
Ccl19	1.04	0.262	0.529, 1.56	3.70E-03	Chemokines & Receptors, Cytokines & Receptors, Dendritic Cell Functions, Inflammation, Leukocyte Functions, T-Cell Functions
Il6ra	1.03	0.174	0.694, 1.37	2.29E-04	CD molecules, Chemokines & Receptors, Cytokines & Receptors, Interleukins
Lbp	1.03	0.244	0.556, 1.51	2.43E-03	Chemokines & Receptors, Inflammation, Innate, Leukocyte Functions, Macrophage Functions, Transporter Functions
Cmpk2	1.02	0.178	0.671, 1.37	2.82E-04	Basic Cell Functions
Lyn	1.02	0.178	0.667, 1.37	2.93E-04	B-Cell Functions, Cytokines & Receptors, Dendritic Cell Functions, Inflammation, Innate, Transporter Functions
Sbno2	1.01	0.196	0.627, 1.40	6.14E-04	Inflammation, Macrophage Functions
Col4a1	1.01	0.233	0.558, 1.47	2.03E-03	Cancer Progression

Note: Values shown are the calculated mean $\log_2$ ratios, SEM and 95% confidence intervals of transcript counts for kidney tissues of $cSiO_2$-treated mice with respect to time-matched vehicle-treated mice 13 weeks post instillation. Statistical analysis was performed using nSolver software (nanoString) with the Benjamini-Hochberg method for control of false discoveries (α=0.05); FDR-corrected q values are shown. Pathway associations for each significant gene are also shown.

Supplementary Table 8. Significant differentially expressed genes in spleen tissues of mice at 13 weeks post $cSiO_2$ instillation (experiment 3).

Gene symbol	Log_2 ratio $cSiO_2$/VEH	SEM	95% CI	FDR *q* value	Associated pathways
Cxcl14	5.80	0.664	4.50, 7.10	2.50E-05	Adaptive, Chemokines & Receptors, Cytokines & Receptors, Inflammation
Ccl8	3.41	0.297	2.83, 3.99	1.09E-05	Adaptive, Cancer Progression, Chemokines & Receptors, Cytokines & Receptors, Inflammation
Fcgr4	3.25	0.343	2.58, 3.92	1.71E-05	CD molecules, T-Cell Functions
Klra2	2.21	0.237	1.74, 2.67	1.81E-05	NK Cell Functions
Il1b	2.03	0.266	1.51, 2.56	5.46E-05	Adaptive, Chemokines & Receptors, Humoral, Inflammation, Innate, Interleukins, Macrophage Functions, Pathogen Response, T-Cell Functions, Transporter Functions
Il21	1.69	0.256	1.19, 2.19	1.35E-04	B-Cell Functions, Cytokines & Receptors, Interleukins, NK Cell Functions, T-Cell Functions
Msr1	1.64	0.269	1.11, 2.16	2.58E-04	CD molecules, Macrophage Functions, Transporter Functions
Itgax	1.60	0.274	1.07, 2.14	3.44E-04	Adhesion, CD molecules, Innate, T-Cell Functions
Il1rn	1.57	0.161	1.25, 1.88	1.68E-05	Cytokines & Receptors, Inflammation, Interleukins
Ccl3	1.55	0.152	1.25, 1.85	1.55E-05	Adaptive, Chemokines & Receptors, Cytokines & Receptors, Humoral, Inflammation, NK Cell Functions, T-Cell Functions, Transporter Functions
Ccl2	1.55	0.210	1.14, 1.96	6.84E-05	Chemokines & Receptors, Cytokines & Receptors, Humoral, Inflammation, Innate, Interleukins, Macrophage Functions, NK Cell Functions, Pathogen Response, T-Cell Functions
Fpr2	1.52	0.253	1.02, 2.01	2.84E-04	Adaptive, Basic Cell Functions, Inflammation
Socs3	1.48	0.202	1.09, 1.88	6.86E-05	T-Cell Functions
Lag3	1.43	0.144	1.15, 1.72	1.59E-05	CD molecules, Interleukins, MHC, NK Cell Functions, T-Cell Functions
Oas2	1.38	0.161	1.06, 1.69	2.50E-05	Basic Cell Functions, Interferon
Cx3cr1	1.35	0.175	1.01, 1.70	5.06E-05	Chemokines & Receptors, Cytokines & Receptors, Macrophage Functions, Microglial Functions
Ccr5	1.25	0.182	0.894, 1.61	9.76E-05	Adaptive, CD molecules, Chemokines & Receptors, Cytokines & Receptors, Dendritic Cell Functions, Macrophage Functions, T-Cell Functions, Transporter Functions
Fos	1.21	0.189	0.84, 1.58	1.70E-04	Inflammation, Innate, Pathogen Response

Ifi44	1.20	0.115	0.972, 1.42	1.55E-05	Interferon
Cfb	1.15	0.127	0.903, 1.40	2.25E-05	Complement Pathway, Innate
Tigit	1.12	0.144	0.838, 1.40	4.79E-05	Interleukins, T-Cell Functions
Dusp4	1.08	0.149	0.786, 1.37	7.43E-05	Basic Cell Functions, Innate
Batf	1.06	0.162	0.746, 1.38	1.39E-04	
Ccr6	1.04	0.168	0.710, 1.37	2.18E-04	Adaptive, CD molecules, Chemokines & Receptors, Humoral, Innate, T-Cell Functions
Xbp1	1.02	0.129	0.773, 1.28	4.67E-05	
Bcl2l1	-1.01	0.207	-1.41, -0.601	1.30E-03	Apoptosis, Cytokines & Receptors, Innate
Cd8b1	-1.24	0.333	-1.89, -0.591	7.12E-03	CD molecules, T-Cell Functions
Il1rl1	-1.30	0.316	-1.92, -0.677	3.91E-03	Chemokines & Receptors, Cytokines & Receptors, Inflammation, Innate, Interleukins, Macrophage Functions, Transporter Functions
Mpo	-1.35	0.394	-2.12, -0.578	1.16E-02	Basic Cell Functions
Tal1	-1.38	0.306	-1.98, -0.780	2.20E-03	Cell Cycle
Ccl24	-1.40	0.306	-2.00, -0.797	2.03E-03	Adaptive, Chemokines & Receptors, Cytokines & Receptors, Inflammation
S100a8	-1.40	0.410	-2.20, -0.595	1.18E-02	Adaptive, Basic Cell Functions, Inflammation
Lcn2	-1.44	0.401	-2.23, -0.658	8.73E-03	Apoptosis, Innate, Transporter Functions
Elane	-1.47	0.407	-2.27, -0.673	8.68E-03	Chemokines & Receptors, Cytokines & Receptors, Inflammation, Interleukins, Leukocyte Functions
Tfrc	-1.50	0.352	-2.19, -0.809	3.15E-03	CD molecules, Transporter Functions
Rsad2	-1.50	0.401	-2.29, -0.713	7.13E-03	Basic Cell Functions
Camp	-1.54	0.416	-2.35, -0.721	7.66E-03	Adaptive, Cancer Progression, Inflammation, Innate
Ctsg	-1.60	0.410	-2.40, -0.793	5.56E-03	Basic Cell Functions
Icam4	-1.95	0.473	-2.88, -1.02	3.82E-03	Adhesion, CD molecules
Cxcl15	-2.83	0.815	-4.43, -1.23	1.17E-02	Cell Cycle, Chemokines & Receptors, Cytokines & Receptors, Inflammation, Interleukins

Note: Values shown are the calculated mean log_2 ratios, SEM and 95% confidence intervals of transcript counts for spleen tissues of $cSiO_2$-treated mice with respect to time-matched vehicle-treated mice 13 weeks post instillation. Statistical analysis was performed using nSolver software (nanoString) with the Benjamini-Hochberg method for control of false discoveries (α=0.05); FDR-corrected q values are shown. Pathway associations for each significant gene are also shown.
